# Expamers: a new technology to control T cell activation

**DOI:** 10.1038/s41598-020-74595-8

**Published:** 2020-10-20

**Authors:** Mateusz P. Poltorak, Patricia Graef, Claudia Tschulik, Michaela Wagner, Vlad Cletiu, Stefan Dreher, Bojana Borjan, Simon P. Fraessle, Manuel Effenberger, Martin Turk, Dirk H. Busch, Juergen Plitzko, David G. Kugler, Seamus Ragan, Thomas Schmidt, Christian Stemberger, Lothar Germeroth

**Affiliations:** 1 Juno Therapeutics GmbH, a Bristol-Myers Squibb Company, Grillparzerstr. 10, 81675 Munich, Germany; 2 Institute for Medical Microbiology Immunology and Hygiene, Technical University of Munich, Munich, Germany; 3 Max Planck Institute of Biochemistry, Am Klopferspitz 18, 82152 Martinsried, Germany; 4 Juno Therapeutics Inc., a Bristol-Myers Squibb Company, 400 Dexter Avenue North, Suite 1200, Seattle, WA 98109 USA

**Keywords:** Lymphocyte activation, Cancer immunotherapy

## Abstract

T cell activation is a cornerstone in manufacturing of T cell-based therapies, and precise control over T cell activation is important in the development of the next generation T-cell based therapeutics. This need cannot be fulfilled by currently available methods for T cell stimulation, in particular not in a time dependent manner. Here, we describe a modular activation reagent called Expamers, which addresses these limitations. Expamers are versatile stimuli that are intended for research and clinical use. They are readily soluble and can be rapidly bound and removed from the cell surface, allowing nearly instantaneous initiation and termination of activation signal, respectively. Hence, Expamers enable precise regulation of T cell stimulation duration and provide promise of control over T cell profiles in future products. Expamers can be easily adopted to different T cell production formats and have the potential to increase efficacy of T cell immunotherapeutics.

## Introduction

Over decades, numerous methods to stimulate T cells in vitro have been established. Most of them take advantage of T cell biology, primarily targeting the engagement of the T cell receptor (TCR) that initiates sufficient intracellular signal transduction and drives productive activation, proliferation, and differentiation^1^. In turn, activation of T cells serves a plethora of purposes in basic research as well as in clinical settings^2–4^. Research on activated T cells helped to understand in detail biological phenomena such as, initiation of immune responses, intracellular signaling, thymocyte development, and T cell memory formation, as well as T cell dysfunction or exhaustion. Stimulation not only allowed prolonged culture of immune cells including selection and expansion of single cell clones but also enabled improved efficiencies of genetic modification methodologies^5,6^. Therefore, T cell activation is also a step during manufacturing of genetically engineered T cells, permitting efficient editing as well as non-clonal expansion to clinically meaningful doses^7^ and selection of an appropriate T cell stimulation reagent to induce adequate T cell responses is of great importance.

Multiple reagents have been developed to activate T cells, from less specific such as PHA mitogen, to more directed like anti-CD3 monoclonal antibodies, to GMP-compliant clinical grade reagents such as antibody-coated microbeads. Typical polyclonal stimuli (that can activate a heterogeneous primary T cell population) are the ones based on at least bi-valent anti-CD3 and anti-CD28 antibodies. Multi-valent binding is necessary, because ligation of the TCR alone (defined as signal 1) will not induce full T cell activation but will rather result in a non-responsive state. Therefore, in addition to the TCR, co-stimulatory receptors—most notably CD28—have to deliver supporting signals (called signal 2). CD28-mediated co-stimulation synergizes with TCR signals promoting survival, clonal expansion, and differentiation^8,9^. In addition to TCR- and CD28-mediated signaling (signal 1 and 2), cytokines such as IL-2 (signal 3) facilitate later stages of T cell stimulation. Hence, it is important to note that activation strength can be also modulated by various culture parameters such as medium composition, cytokine milieu, culture method, and donor cells.

A mix of soluble anti-CD3 and anti-CD28 antibodies can only trigger a short-lived activation that does not lead to productive responses as they are not able to induce proper formation of immunological synapses and fail to provide focal signals^10,11^. Thus, in most of the cases a modulation of the surface interaction becomes necessary^12^. Therefore, in research-related and clinical applications at least one of the aforementioned antibodies is surface-bound. Surface-bound antibodies are available in many varieties with the most commonly used being bead- or plate-based solid supports but also covering some other forms of spatial binding organization like feeder cells or more recently lipid bilayers^13,14^.

All of these polyclonal stimuli exploit the principle that cross-linking and clustering of adequate number of TCR complexes creates a favorable intracellular microenvironment for kinases to phosphorylate a sufficient number of molecules to overcome the activation threshold of several signaling pathways ultimately leading to T cell activation^15–17^. Multiple anti-CD3 antibodies can simultaneously interact with several CD3 subunits of adjacent TCR complexes bringing them into close proximity. A sufficient number of clustered TCR complexes creates a zone on the T cell surface (micro-synapse) that excludes phosphatases and favors kinases^18^. This shift in enzymatic balance triggers phosphorylation of molecules involved in TCR-mediated signaling and subsequently, downstream signal propagation^16,19^. It is a process that closely mimics the natural T cell activation, which depends on aggregation of TCRs upon the recognition of cognate antigen peptide and subsequent immunological synapse formation^1^. A similar principle for cell activation is employed with the use of artificial receptors, such as chimeric antigen receptors (CARs), as well as for antigen-specific T cell expansion, where anti-CD3 antibody is replaced with an MHC I molecule loaded with a cognate peptide^20^. For most productive responses cross-linking of either CD28 and/or 41BB is used in addition to TCR/CD3^21,22^.

This simple but effective basis for T cell stimulation can generate an array of T cell responses from bringing T cells into the state of anergy to differentiating them into distinct subsets. For example, naïve and memory T cells do have defined activation thresholds that can be exploited for preferential expansion during stimulation^23–25^. Importantly, strong signals in CD8^+^ T cells lead to apoptosis, whereas low activation levels will result in proliferation^11^. Comparably, CD4^+^ T cell polarization can be also controlled by signal intensity^26,27^. In addition, signal duration can also modulate T cell functionality as induction of transient signals corresponds with unresponsiveness or apoptosis, in contrast to sustained signaling, which results in activation and proliferation^10,11,28^. Finally, it has been postulated that the magnitude of activation signal is even important for T cell maturation^29^.

To elicit control over these variables, we developed, on basis of the recently described Streptamer principle, an activation reagent called Expamers that overcomes some of the shortcomings of other T cell stimuli^30^. Expamers are polymeric, soluble protein complexes designed to activate human primary T cells without the use of solid support while still providing extended contact surface areas. For generation of Expamer reagents, low-affinity monovalent anti-CD3 and anti-CD28 antibody Fab fragments—carrying the Twin-Strep-tag affinity tag—are used to functionalize chemically polymerized Strep-Tactin (mutein of streptavidin) multimer backbones. These complexes deliver the activation signal by multivalent binding of the anti-CD3 and anti-CD28 Fab ligands to the target TCR and CD28 co-stimulatory receptors initiating a cellular response similar to other antibody-based methods. Solubility of the Expamer reagents permits almost instantaneous distribution of the activation signal in cell culture and hence a very precise temporal control of onset activation. Moreover by exploiting the reversibility of Twin-Strep-tag:Strep-Tactin interaction, not only the signal initiation but also signal termination can be instantly and precisely regulated with addition of non-toxic D-biotin^31–33^. Noteworthy, upon D-biotin addition the henceforth monovalently-interacting, affinity-reduced Fab fragments will rapidly dissociate from T cell surface leaving the cells within second to minutes reagent free. These combined properties position Expamers as a unique type of T cell stimulus that enables control of T cell activation duration. Additionally, Expamers are modular reagents where Strep-Tactin multimer backbone can bind any Strep-tagged molecule of choice enabling the flexible stimulation of variety of different T cell sources (e.g.: CAR T cells, antigen-specific T cells). Finally, the stoichiometry and density of Expamer Fab ligands can be easily adjusted to further fine tune the activation signal.

The primary purpose of Expamers is to activate and expand a population of T cells during in vitro cultivation and to facilitate viral transduction for CAR T cell manufacturing.

## Results

### Molecular structure, composition and function of Expamers

Next-generation T cell manufacturing processes may benefit from modular activation reagents that offer additional levels of control in comparison to current state-of-the art stimuli. Therefore, we designed a readily soluble and solely proteinaceous T cell activation technology—Expamers, which can be used in closed manufacturing equipment and can be rapidly bound to and removed from the target cell surface, allowing instantaneous initiation and termination of activation signals, respectively.

**Figure 1:**
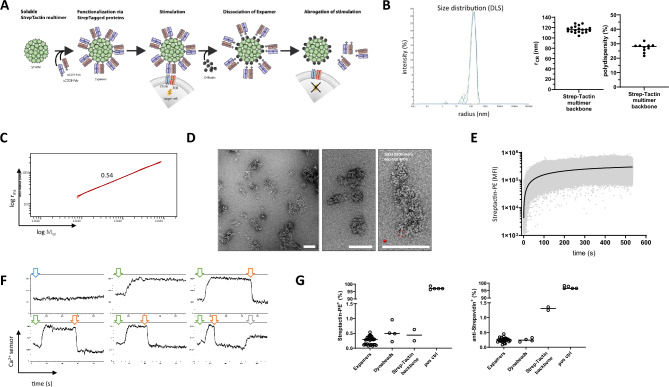
Biophysical properties of Expamers. (**A**) Schematic visualization of Expamers mode of action. Expamers spontaneously assemble form single components (anti-CD3 and anti-CD28 Fab fragments as well as Strep-Tactin multimer backbone). Assembled Expamers interact with target T cells by binding to and cross-linking TCR and CD28 surface receptors. Subsequently, Expamers dissociate upon addition of D-biotin. Dissociation results in removing of Expamers from the T cell surface and termination of the activation signal. (**B**) Histogram overlay of radius measurements of three different Strep-Tactin multimer backbone lots (green, yellow, blue). Graphs depict average hydrodynamic radii from cumulative fits of 19 individual Strep-Tactin multimer backbones as well as polydispersity measurements for different manufacturing lots. (**C**) The confirmation plot plotting the variation of the molar mass (an average molar mass of 1.29 × 10^8^ Da corresponds to an average amount of approx. 2400 Strep-Tactin tetramers) against the variation of the radius of gyration (92 nm in average). The slope of 0.54 indicates the conformation of a random coil. (**D**) Electron microscopy images of negatively stained Strep-Tactin multimer show a pleomorphic backbone with mesh sizes ranging from 30 to 300 nm depending on the crosslinking conditions. Individual Strep-Tactins can be observed on the surface of negatively stained multimer (zoom-in). A streptavidin (PDynabeads: 6J6J, red) filtered to 20 Å is inserted for reference. The scale-bars are 100 nm. (**E**) Graph displays the change of signal intensity over time of CD3^+^ cells (pre-gated on live, single cells) interacting with fluorescently labelled Strep-Tactin (Strep-Tactin-PE) pre-assembled with anti-CD3 and anti-CD28 Fab fragments. Within seconds all cells became Strep-Tactin-PE-positive underlying the speed of Expamers activation potential. Graph is a representative of three independent measurements. (**F**) Representative histograms of calcium flux measurements of five independent experiments as shown. Time point of Expamer addition is indicated by green arrows. Time point of D-biotin addition is indicated by red arrows. Ionomycin addition is indicated by a grey arrow and addition of Fab fragments only is indicated by a blue arrow. (**G**) Graphs display residual content of Expamer components; either Fab fragments detected using Strep-Tactin-PE (left panel) or Strep-Tactin multimer backbone detected using an anti-streptavidin antibody (right panel) 8 days after activation. ‘Expamers’ column represents T cells cultured in the presence of Expamers for 8 days. Dynabeads and Strep-Tactin multimer backbone only were used as a negative controls and Expamer-stained T cells (without dissociation) as a positive control (pos ctrl). Graphs show cumulative data from four independent experiments. Lines represent mean ± SD.

For standard T cell activation purposes, Expamers rely on proteinaceous Strep-Tactin multimer backbone molecules functionalized with Strep-tagged anti-CD3 and anti-CD28 Fab fragments as functional constituents that self-assemble in solution (Fig. 1A). Fab fragments are heterodimers comprising the heavy and the light chain of an antibody. Strep-tags are octameric peptide tags for highly specific but reversible interaction of desired ligands, such as antibody derived Fab fragments to the biotin-binding pocket of a Strep-Tactin molecule^31,32,34^. The generation of a multimeric Strep-Tactin backbone molecule by chemical polymerization enables Fab fragment complexation (Expamer self-assembly). Hence, the Strep-Tactin multimer backbone facilitates stable cell:Expamers interactions and transmission of specific signals through engagement of surface CD3ε and CD28 molecules (Fig. 1A). Upon the addition of D-biotin, Fab-functionalized Strep-Tactin multimers can be rapidly dissociated by displacing Strep-tags from Strep-Tactin and subsequent (monomeric) Fab fragment dissociation is facilitated by fast off-rates (e.g. the CD3 Fab fragment is based on clone OKT3 having an already low known affinity of 2–3 µM with fast on- and off-rates (1.97 × 10^5^ M^−1^ s^−1^ and 4.8 × 10^–1^ s^−1^))^35^. Thus, D-biotin enables timely-resolved dissociation of Fab fragments and Strep-Tactin multimer backbone resulting in immediate signal interruption and removal of individual Expamers components from the cell (Fig. 1A). The described Strep-tag:Strep-Tactin interaction has already led to several research and clinical applications^30,36–40^.

Average D-biotin binding capacity of a Strep-Tactin tetramer within Strep-Tactin multimer backbone was determined to be approximately 2.8 mol of D-biotin per 1 mol of tetramer. Considering the molecular weights of the Strep-Tactin tetramer (52,800 Da) and of Fab fragments (50,000 Da), approximately 1 g of Fab fragments saturates 0.75 g of Strep-Tactin multimer backbone, assuming that both Strep-tags moieties of the Twin-Strep-tag at the C-terminus of each Fab (heavy chain) contribute to the binding. This assumption was used as a framework for further titration experiments and the stoichiometry of each Fab fragment and the backbone was extensively tested in multiple culture systems (Supplementary Fig. S1). As an outcome, the final composition of Expamers was optimized to accommodate T cell activation in a variety of cell culture media (both in the presence and absence of serum and/or cytokines). Of note, media selection was done under D-biotin content consideration as Expamers tolerate up to 12 µM of D-biotin in culture without detrimental effects on their function.

For efficient T cell activation, it is important that Expamers maintain both their solubility as well as provide a sufficient binding area. The Strep-Tactin multimer backbone has a random coil-like spatial structure and an average hydrodynamic radius of over 100 nm as indicated by DLS (dynamic light scattering) measurements and visualized in electron microscopic images (Fig. 1B–D). This average particle size of approximately 200 nm in diameter (with an overall maximal size distribution range in manufacturing batches from > 50 to < 800 nm) has been demonstrated as optimal for productive T cell activation^20^ while maintaining solubility of the Expamers. The Strep-Tactin multimerization process has been optimized to reproducibly deliver multimer backbones in this desired size range (Fig. 1B).

As Expamers are formed by self-association of individual soluble components, we were interested in the underlying self-assembly kinetics. We measured dynamics of self-assembly as well as the speed of complete Expamer binding to a purified CD3^+^ T cell suspension by sequentially injecting individual Fab fragments followed by PE-labeled Strep-Tactin multimer backbone and continuously monitoring cells using flow cytometry (Fig. 1E). As anticipated, fully functionalized Expamers formed and attached to target cells almost instantaneously (Fig. 1E). Thus, anticipated rapid penetrance of Expamers does not require stress-inducing manipulations such as centrifugation to synchronize the activation signal across cell culture.

Next, we analyzed onset and termination rates of Expamer-mediated activation by online calcium flux measurements with or without timely controlled addition of D-biotin (Fig. 1F). Addition of Fab-loaded Expamers to T cell suspensions resulted in immediate calcium influx that was sustained over the entire follow-up time. Addition of D-biotin immediately abrogated calcium release with very fast turnaround time back to baseline level, indicating a prompt interruption of activation directly at the selected time point. Importantly, this action did not render T cells unresponsive, as they could be subsequently reactivated by ionomycin (Fig. 1F). Interestingly, D-biotin-mediated removal of Expamers is very efficient and after maintaining cells in culture minimal-to-no residual Expamers components are detectable (Fig. 1G).

Finally, Expamers lot-to-lot comparability based on an Expamer component matrix (anti-CD3 and anti-CD28 Fab fragments as well as Strep-Tactin multimer backbone) resulted in a very consistent data set on all parameters tested (Fig. 1B and data not shown). Overall, Expamers production process is very robust and guarantees the highest consistency and quality of the final reagents.

### Expamers are potent T cell stimuli

**Figure 2:**
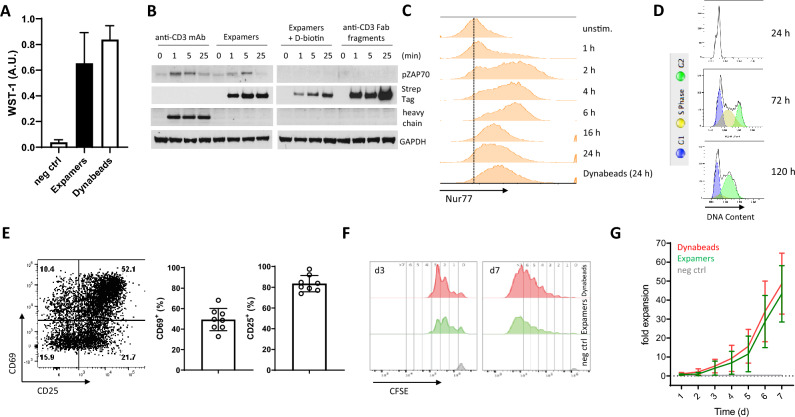
Expamers are a potent T cell activation reagent. (**A**) Graph shows changes in WST-1 reagent depicted as arbitrary units 48 h after activation with either Expamers or Dynabeads from four independent experiments. Bars represent mean ± SD. Difference between the two stimulation conditions was not significant (two-tailed paired Student’s t test). (**B**) Human primary T cells were incubated at 37 °C with OKT3 monoclonal anti-CD3 antibody (positive control), anti-CD3 Fab-loaded Expamers in the presence or absence of D-biotin or anti-CD3 Fab fragments only (negative control) for the indicated timepoints. Cells were lysed and cytoplasmic extracts were analyzed for ZAP70 kinase phosphorylation using SDS-PAGE and Western Blot. GAPDH was used as loading control and anti-Strep-Tag as well as anti-heavy chain secondary antibodies were used for reagent detection. One representative blot of three independent experiments is shown. Full-length blots/gels are presented in Supplementary Fig. S8. (**C**) Histograms from one representative experiment from four independent measurements shows changes in fluorescence intensity of tdTomato-Nur77 reporter in a Jurkat T cell-line over time upon activation with Expamers. For Dynabeads control, 24 h time point is shown. (**D**) Histograms from one of two independent experiments represent changes in DNA content over time upon Expamers activation. (**E**) CD69 and CD25 surface marker upregulation was detected 24 h after stimulation with Expamers using flow cytometry. T cells were pre-gated on live, single CD3-positive cells. One representative dot plot is shown. Graph summarize data from 8 independent measurements. Bars represent mean ± SD. (**F**) Histograms present cell cycle entry and proliferation that were assessed by measuring CFSE dilution of CFSE-labeled T cells by flow cytometry at day 3 and day 7 time point. Numbers on top of the histograms refer to the number of cell division within each generation. (**G**) T cell proliferation is represented by fold expansion graph that is a cumulative data from at least four experiments. Negative control is represented by unstimulated cells. Lines represent mean ± SD. Difference between two stimulation conditions was not significant (two-tailed paired Student’s t test).

To investigate Expamer-mediated T cell activation in vitro, we assessed general biologic responses upon stimulation including early-to-late changes in physiological properties. To this end, metabolic conversion of WST-1, microscopic analysis as well as assessment of activation markers (ZAP70, Nur77, CD69 and CD25) and cell cycle state were selected as read-outs (Fig. 2; Supplementary Figs. S2, S3). In certain experiments CD3/CD28 functionalized Dynabeads reagents were included as reference. Of importance, the selection of Dynabeads as benchmark reference was mandated by their broad use for T cell activation especially in clinically relevant settings and does not serve as direct qualitative comparison because of physico-chemical and biological differences of both reagents.

The WST-1 assay was used to indicate early (measured 48 h post start of activation) metabolic change in T cell function^41^ through reduction of colorimetric WST-1 reagent that occurs prior to expansion. As shown, Expamers potently induced a metabolic switch from oxidative phosphorylation towards oxidative glycolysis in T cells that was comparable to the known strong Dynabead-mediated early activation (Fig. 2A). In line with increased metabolic activity, Expamer-stimulated T cells rapidly formed clusters indicative of T cell active state (Supplementary Fig. S2). Mechanistically, Expamers triggered major phosphorylation cascades associated with TCR-mediated signaling (Fig. 2B,C). Upon stimulation with Expamers, ZAP70—a crucial kinase of proximal TCR-mediated signaling pathway—was phosphorylated within minutes but upon D-biotin addition the phosphorylation could be controlled (as indicated by detection of anti-Strep-Tag signal but no ZAP70 phosphorylation) (Fig. 2B). Concomitantly, strictly TCR-dependent transcription factor Nur77 was activated within the first few hours (Fig. 2C). Nur77 reached maximal expression levels at 6 h time point and was maintained for at least 24 h, which was in line with previously reported kinetics^42^. Furthermore, the early activation markers CD69 and CD25 were highly upregulated and readily detectable on the cell surface shortly after Expamer-mediated activation (Fig. 2E). Both CD69 and CD25 were also present on the cells surface for prolonged periods of time (Supplementary Fig. S3). These results confirmed that the activation signal was long-lasting, which is a prerequisite to cross activation threshold requirement and consequently to enter cell cycle and generate a proper T cell response (Fig. 2C–E). Increased DNA content in cells clearly indicated progression from G1 to G2 phase for the majority of the T cell population (Fig. 2D).

To measure subsequent T cell proliferation, cell numbers as well as CFSE dilution profiles were evaluated in extended periods of cell culture (Fig. 2F,G). T cells did dramatically increase in numbers once more indicating very efficient activation (Fig. 2G). Expamers were able to trigger rapid doubling rates (average of at least one division per 24 h) that translated into reaching up to 60-fold expansion within a week. Short (measured at day 3 post-stimulation) and long term (day 7) CFSE dilution measurements indicated that the majority of T cells underwent at least one division and thus confirmed cell number data (Fig. 2F). Notably, similar results were obtained in several different culture systems including serum-free media relevant in clinical settings (data not shown).

### T cells express beneficial and homogenous phenotypic and expression profiles upon Expamers stimulation

**Figure 3:**
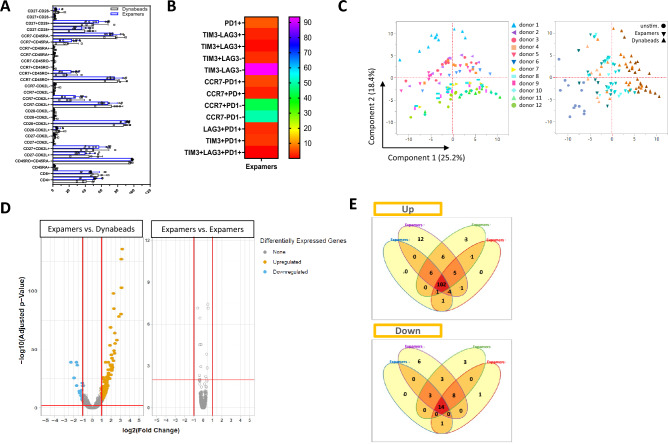
Expamers generate distinct but consistent T cell phenotypic and genetic profiles. (**A**,**B**) T cell phenotypic profiling was conducted by measuring surface expression of indicated surface markers by flow cytometry. Bar graph and Heat map present cumulative data from four donors. Error bars in (**A**) represent mean ± SD. (**C**) Analysis by PCA indicated that the Expamers titration did result in titration-dependent surface markers expression profiles for selected donors with the Dynabeads treatments distinctly different. Low Expamers and Dynabeads conditions resulted in more comparable T cell profiles. Greater difference was observed between donors (Component 2) than among conditions (Component 1). PCA analysis was based of T cell surface marker expression of 12 donors detected by flow cytometry (left panel, each donor indicated by different color and symbol shape). T cells were left unstimulated (grey shapes on right panel) or were stimulated for 24 h before data collection using either varying concentrations of Expamers (900%, 300%, 100%, 30%, 10% of standard concentration per 1 × 10^6^ T cells; blue gradient on right panel, darker color equals to higher Expamer dose) or different bead-to-cell ratios (3:1, 1:1, 1:3, 1:9, 1:18; orange-to-brown gradient on right panel, darker color equals higher Dynabeads content). (**D**) Depicted are volcano plots of the -log_10_ adjusted P value vs. log_2_ fold change with differentially expressed genes highlighted in color for one out of four representative Expamers condition. Differentially expressed genes were selected by imposing a log_2_FC cutoff of 1 and Benjamini–Hochberg adjusted FDR cutoff of 0.1. Similar results were achieved for other three Expamers formulations (not shown). (**E**) The Venn diagram was generated to show that the different individual Expamers conditions (as in **D**) yielded similar differentially expressed genes compared to Dynabeads stimulation independent of titration conditions for both upregulated (top) and downregulated (bottom) genes. Differentially expressed genes were selected by imposing a log_2_FC cutoff of 1 and Benjamini–Hochberg adjusted FDR cutoff of 0.1.

To study the impact of Expamer activation on T cell state, flow cytometric analysis of surface phenotypes of cells after primary activation (24 h time point) as well as broader mRNA analysis were performed (Fig. 3). Surface detection of markers used to identify different T cell subsets or cell differentiation states revealed phenotypic patterns associated with less differentiated T cells, characterized by high expression levels of CD62L, CD27, CD28, and CCR7. Of note, the fulminant proliferation and strong polyclonal in vivo activation may add a considerable bias towards T cell characteristics that will not reflect ex vivo phenotypes. With this in mind, a trend in CCR7 and CD27 expression patterns between Expamers and Dynabeads stimulated T cells was observed (Fig. 3A). Interestingly, analysis of three prominent negative-regulatory markers PD1, LAG3, and TIM3 associated with T cell exhaustion revealed that cells might be of high functional capacity, as there was overall only marginal surface expression visible. Most prominent was upregulation of PD1 but TIM3 and LAG3 were hardly detectable (Fig. 3B). Importantly, there were no T cells present expressing more than one negative regulator at the same time, which is considered to be more closely associated with exhaustion rather than transient expression of these markers during primary activation (Fig. 3B).

To compare impact of stimuli more globally, Principle Component Analysis (PCA) was performed on a selected T cell profile sample set 24 h after initial stimulation (Fig. 3C). Here, both Expamers and Dynabeads were titrated for activation. Content of Expamers per 1 × 10^6^ T cells was titrated from up to 900% and down to 10% of standard dose, whereas Dynabeads were varied in bead/cell ratio (Fig. 3C). Samples grouped by donor (Component 2) and by activation reagent (Component 1). Interestingly, although Dynabeads and Expamers generated distinct T cell phenotypes, lower amounts of both stimuli correlated more closely to each other (Fig. 3C, right panel, lighter colors). This analysis suggests that at that specific time-point the discriminants in cell phenotypic profiles are (1) cell origin (donor) and (2) type of the stimulus (Expamers vs. Dynabeads).

To further evaluate T cell profiles, in-depth mRNA expression analyses were performed 7 days after initial stimulation (Fig. 3D,E). As a means of increasing power of the experiment, Expamers were modified (four slightly different anti-CD3 and anti-CD28 Fab ratios that did not show any impact on T cell phenotype) and used to activate T cells from two donors in separate experimental arms. Dynabeads served as control stimulation reagent. Subsequently, whole mRNA from activated T cells stimulated either with different Expamers or Dynabeads was collected for in-depth mRNA analysis. QC evaluation of the obtained library passed all requirements (RIN, mapped read counts, expression profiling efficiency etc.). In total, we obtained > 25 × 10^6^ reads per sample with > 90% mapping efficiency with a uniform coverage of genes across all samples. All samples uniformly had ≥ 7000 genes accounting for 95% of the total mapped reads.

To investigate differences in the gene expression profiles of the T cells, several analyses were undertaken (Fig. 3D,E, Supplementary Fig. S4 and collapsed list of all differentially regulated genes in Supplementary Table S1). As displayed in volcano plots, Expamers induced a distinct gene upregulation profile compared to Dynabeads but maintained a consistent gene expression state across different batches (Fig. 3D). Approximately 150 different and significant gene signatures were upregulated in each Expamers sample as compared to Dynabeads reference (Fig. 3D,E). Within these 150 upregulated genes around 100 were shared by all other Expamers treatments. Only 15 genes were unique (Fig. 3E). In conclusion, Expamers stimulation leads to different mRNA expression profiles than Dynabeads activation but this profile is consistent within different Expamers compositions. These data were in line with the functional and phenotypic results described previously. Noteworthy, according to our analysis no specific pathway that would strongly differentiate one stimulus from the other has been identified. Nonetheless, elevated expression levels of genes related to T cell activation, T cell effector functions, and T cell differentiation was measured in Expamers-stimulated cells at that specific time-point (Supplementary Fig. S4; Supplementary Table S1). These differences are most likely resulting from different (potentially steadier) dynamics of activation upon using Expamers leading to an apparently stronger activation profile at the later time point of measurement.

### Tight control of duration of T cell activation and flexibility of Expamers

**Figure 4:**
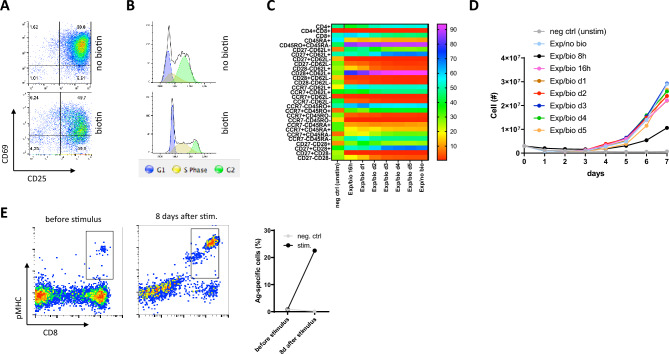
Expamers enable control over stimulation duration. (**A**,**B**) T cells were stimulated with Expamers. 48 h post-activation D-biotin was added or not to the T cell culture and T cell activation state (**A**) as well as cell cycle phase (**B**) was assessed by flow cytometry 24 h later. (**C**,**D**) T cells were stimulated as in (**A**) or left unstimulated. Subsequently, D-biotin was added to the culture at indicated time points. Surface marker expression (**C**) and proliferation (**D**) was measured by flow cytometry and cell counting, respectively. (**E**) Population of HLA-A*0201^+^ CMV seropositive donor was analyzed before and after 8 days antigen-specific stimulation using either pMHC- and CD28 Fab-loaded multimer backbone or Expamers (polyclonal stimulus—negative control). Events were pre-gated on single, living T cells.

Previous results demonstrated that Expamers are fully capable of inducing (1) fast and (2) robust T cell activation that will lead to proliferation and differentiation and is at least comparable to other more established activation reagents (Figs. 2, 3). Yet, what strongly differentiates Expamers from other T cell stimuli is the feature of reversibility. Precise control over the duration of T cell activation may enable regulation of the cellular activation state (Fig. 4A,B). Shorter stimulation signals can potentially generate T cells of less activated/exhausted phenotype that is favorable for long-lasting activity. Along this line, disruption of Expamers by addition of D-biotin at early time points of T cell culture may result in differences in the cellular phenotype at harvest (Fig. 4C). As shown, early addition of D-biotin may help maintain more naïve-like T cell profiles (higher content of CD45RA+ and lower of CD45RO+) (Fig. 4C). Importantly, early (> 16 h) signal interruption with D-biotin does not impede T cell expansion (Fig. 4D). Hence, Expamers are adequate reagents to control T cell stimulation duration while simultaneously providing efficient activation.

Furthermore, Expamers activation can be extended to more specific T cell subpopulations. One example of a relevant application is antigen-specific activation for directed expansion (Fig. 4E). Here, the polyclonal anti-CD3 Fab fragment was replaced by pMHC against a given TCR specificity. Upon the addition of MHC-modified Expamers to PBMC culture, only T cells with the respective antigen-specific TCR were able to expand in a span of 8 days (Fig. 4E). This application exemplifies the modular potential of Expamers.

### Clinical application: generation of CAR T cells using Expamers

**Figure 5:**
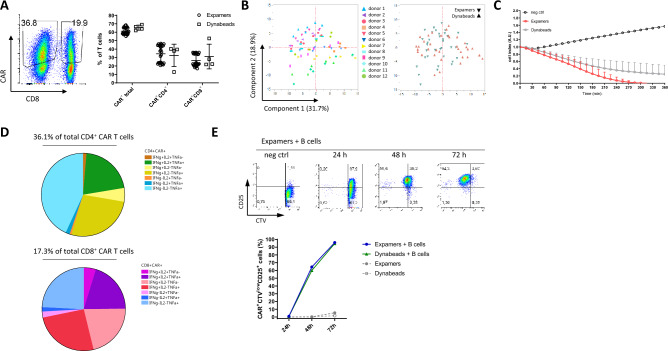
Expamers are suited for CAR T cell generation. (**A**) T cells stimulated either with Expamers or with Dynabeads were cultured for 7 days. On the last day, expression of CAR was assessed by flow cytometry. One representative dot plot is shown pre-gated on single, living CD3^+^ cells. Graph displays cumulative data from four different donors with multiple technical replicates each. Lines represent mean ± SD. Difference between two stimulation conditions was not significant (two-tailed paired Student’s t test). (**B**) PCA analysis of CAR T cells was performed on the same sample set indicated in Fig. 3C but after 7 days of culture. Left panel shows donor distribution (different color and shape for each donor), whereas right panel displays two activation reagents (blue—Expamers, orange—Dynabeads). (**C**) CAR T cells generated like in (**B**) were frozen and stored in liquid nitrogen for given time. Afterward, CAR T cells were thawed and rested for 24 h before adding to CD19-HEK target cells in a 5:1 E:T ratio. Graph depicts changes in impedance indicated as arbitrary units over time that is a read out for cytotoxic abilities of CAR T cells from three different donors. CD19-HEK cells co-cultured with T cells expressing an irrelevant CAR were used as negative control. Lines represent mean ± SD. (**D**) Pie charts show on-target cytokine production by CD4^+^ or CD8^+^ CAR T cells from (**C**) based of flow cytometry measurement of intracellular staining 24 h after co-culturing with target cells. Cells were pre-gated on live, single, CD3^+^CAR^+^ cells. Charts display mean values of cytokine secretion of CAR T cells manufactured from three healthy donors. (**E**) After removal from cryo-storage, CAR T cells were stained with CTV-dye and either left unstimulated or re-stimulated with CD19-expressing B cells from healthy donor. After 24, 48, and 72 h cells were analyzed for dilution of CTV dye (proliferation) and surface expression of CD25. Representative dot plots are shown. Cells were pre-gated on live, single, CD3^+^CAR^+^ cells. Graph depicts CAR T cell activation defined as frequency to CAR^+^CTV^low^CD25^+^.

**Figure 6:**
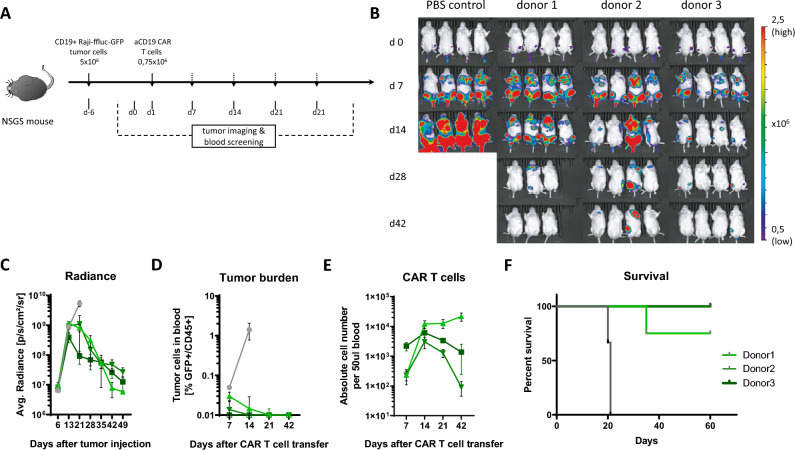
In vivo function of Expamer-stimulated anti-CD19 CAR T cells. (**A**) CD19^+^ Raji/ffluc/GFP tumor cells were engrafted for 7 days in NSGS mice, subsequently 0.75 × 10^6^ CAR T cells (bulk) were infused on day 1. (**B**) Tumor was imaged using IVIS bioluminescence at indicated time points. (**C**) Average radiance was quantified using Living image. (**D** and **E**) Absolute cell count of CAR T cells in blood was calculated using cell count and frequencies gathered from flow-cytometry analysis. Frequency of tumor cells in blood (CD45^+^/GFP^+^) was measured via flow-cytometry illustrated over time. Cells were pre-gated on living lymphocytes. (**F**) Survival analysis of each group is shown. PBS control is shown in grey. Statistical analysis: log-rank Mantel–Cox test; ***P ≤ 0.0007, n = 4 mice per group, total of 12 mice. Means ± SEM are plotted.

Expamers were designed to activate T cells for any given application. Currently, one of the most relevant clinical use of T cell stimuli is in engineered T cell manufacturing. To prove that Expamers are appropriate for the generation of transgenic T cells, we performed a series of experiments demonstrating transgene expression efficiency and downstream functionality of manufactured CAR T cells (Figs. 5, 6). To this end, we used an established anti-CD19 CAR transgene, which co-expresses the EGFRt safety tag^43^.

Cumulative transduction efficiencies revealed an average of 60 ± 10% total CAR T cells as measured by EGFRt staining (Fig. 5A). Similar transduction efficiencies were also observed for Dynabeads activated cells. Individual transduction frequencies/efficiencies for individual CD4^+^ or CD8^+^ T cells were found to be in average 65 ± 10% and 55 ± 10%, respectively, indicating overall similar activation capabilities of Expamers for CD4^+^ and CD8^+^ T cells (Fig. 5A). Notably, Expamers proved to be also compatible with electroporation and non-viral gene delivery, i.e. using the CRISPR/Cas9 system (Supplementary Fig. S5). The CAR T cells phenotype was also assessed using PCA analysis and appeared to be mostly overlapping with the Dynabeads reference (Fig. 5B). To this end, same samples analyzed in Fig. 3C were further cultured for 7 days and at harvest their surface protein expression (including CAR) was analyzed once again. These results exemplify that modulating the amount and/or type of anti-CD3/CD28-based activation reagent only may be insufficient to control T cell fate in prolonged cell cultures where T cells will integrate additional different signals from the environment.

To address whether the use of Expamers would result in an alteration/reduction in the promiscuity of the CAR-positive T cell population, the usage of the TCR Vβ repertoire was analyzed. Supplementary Fig. S6 shows almost identical patterns for the Vβ repertoire of CAR-positive CD4^+^ and CD8^+^ T cells between different activation conditions, implying no significant differences between the reagents in changing the relative distribution of T cell clones in the samples evaluated. In addition, the correlation analysis between activated-expanded cells and their unstimulated original repertoire before culture, for two independent experiments performed on 7 and 14 days after activation (5 and 12 days post transduction), also revealed no significant bias towards certain stimulation/activation reagent dependent Vβ usage.

The homogenous subset distribution within the CAR^+^ populations already observed in the phenotypic apperance also rendered manufactured cells equally functional, as measured by the antigen-specific killling of CD19-transfected HEK target cells by either Expamers or Dynabeads-stimulated T cells (effectors) (Fig. 5C). After initiation of effector:target (E:T) cells co-culture, CAR T cells excerted immediate effector functions and rapid killing of target cells that was observed and maintained for a follow up of about 24 h. Killing efficiency was associated with a on-target CAR-specific production of the pro-inflammatory cytokines IFNγ and TNFα as well as IL-2 (Fig. 5D; Supplementary Fig. S7). Many of the cytokine producing cells also exhibited polyfunctionality and secreted more than one of the cytokines as double- or triple-producers (Fig. 5D). The relatively low frequency of cytokine-producing cells was likely associated with an early re-stimulation time-point post-thaw (re-stimulation occurred already at 24 h post removal from frozen condition). As a positive control, non-specific PMA/ionomycin stimulation resulted in a profound production of cytokines (data not shown). These data indicate that Expamers render functional CAR^+^ cells capable of mounting antigen-specific functions. The high functional state was also in line with the very low amounts of combined expression of exhaustion makers during manufacturing (Fig. 3B) and secondary expression of these markers during 24 h in vitro killing was very little to none (data not shown).

To further assess the response of manufactured CAR T cells, we evaluated the kinetics of re-activation of cells retrieved from cryo-preservation. Re-stimulation of CAR T cells with CD19-expressing autologous B cells resulted in robust re-activation measured by CD25 expression and proliferation (CTV dye dilution). After 24 h, up-regulation of CD25 was already observed compared to thawed T cells that were left unstimulated (Fig. 5E, lower panel). However, T cells have not started to divide (no dilution of CTV dye). After 48 h, about 60% of CAR T cells went through an initial cell division cycle accompanied by continuous expression of CD25. 72 h after activation, all cells divided at least once (Fig. 5E). Cell re-activation kinetics was very robust regardless of the activation reagent used during manufacturing (Fig. 5E, lower panel).

Finally, we studied in vivo functionality of Expamer-activated CD19-specific CAR T cells in a well-established and clinically relevant mouse model (Fig. 6). Total of 5 × 10^5^ Raji-ffluc-GFP CD19^+^ tumor cells (i.v.). and only 7.5 × 10^5^ CAR^+^ T cells from three different donors were injected into mice (i.v.) on day 6 and day 1, respectively (Fig. 6A). Mice were bled weekly for blood analysis as well as weight and tumor burden were monitored to trace tumor growth or eradication (IVIS). In the control group consisting of mice treated with PBS, a rapid tumor progression was observed followed by high lethality (Fig. 2B). Mice treated with CAR T cells showed an expected increase of tumor radiance within the first 7 days after CAR T cell injection, however we observed a drastic reduction in tumor size between day 7 and 14 (Fig. 6B,C). After 42 days, CAR T cells cleared the tumor in most of the mice (Fig. 6B). Considering tumor radiance, CAR T cells from all donors performed comparable in respect to tumor elimination (Fig. 6C). Coinciding results were observed when analyzing expansion of transferred CAR T cells and tumor burden in blood with peak expansion of CAR T cells being detected at day 14 (Fig. 6D,E). These results translated into high survival rate of all animal groups (Fig. 6F).

In conclusion, Expamers are well suited for CAR T cell manufacturing as they can produce fully functional CAR^+^ cells.

## Discussion

Results presented in this manuscript describe a T cell activation technology called Expamers. Expamers deliver a unique type of reagent that provides support surface area for T cell receptors cross-linking and is still soluble. Additionally, due to implementation of reversibility, Expamers enable not only a precise control over initiation of T cell activation but also over signal termination. The latter property offers an important advantage over other conventional activation reagents available on the market. Although signal termination is possible with other stimuli types such as Dynabeads, it is technically more challenging and may lead to high cell losses and increased cell handling risks (especially at early time points post-activation). Therefore, Expamers optimally support temporal regulation of T cell activation signal and potentially allow more precise determination of the resulting cellular profile especially in advanced shorter manufacturing processes that may very well translate into promoting T cell phenotypes, which are beneficial for immunotherapies^44–46^. For example, this may relate to conservation and/or promotion of central memory and other less differentiated T cell phenotypes that were demonstrated to better support persistent responses in vivo^47^.

Expamers’ ease-of-use has additional advantages. Non-viral transduction methodologies require an electroporation step not always compatible with using solid (paramagnetic) particles, hence negatively impacting transfection efficiency and cell recovery. The proteinaceous origin of Expamers permits electroporation without the need of reagent removal. Therefore, neither efficiency nor cell numbers/viability are significantly affected by the presence of Expamers during gene editing procedures that helps enabling cutting-edge technologies like CRISPR/Cas9 amenable to T cell manufacturing^6,48^. Expamers are also suitable in large-scale production settings as they reduce overall and hands-on time of stimulation operation and have been introduced to clinical manufacturing just recently (NCT04231747).

Since the basis of Expamer technology is the Strep-tag:Strep-Tactin interaction, incorporating Strep-tag affinity tags into different molecules (e.g. antibodies against co-stimulatory receptors) can lead to completely new Expamer-like moieties. This modularity creates great opportunities to extend the application spectrum of Expamers. As exemplified in this manuscript, Expamers may include molecules that can stimulate particular T cell subpopulations.

All these features position Expamers as a suitable reagent for clinical applications. Importantly, T cells activated with Expamers should pose low-to-none safety risk in patients undergoing any form of immunotherapy. Firstly, all Expamer components are efficiently disrupted upon introduction of D-biotin and any remaining reagents can be promptly washed away from the cell culture. Secondly, single Fab fragments have fast dissociation kinetics from respective receptors and they will dissociate from the cell surface shortly after D-biotin addition. Moreover, their re-binding capacity as a monovalent molecule is very low (particularly at the used dilutions) and a single Fab fragment is incapable of receptor cross-linking. Thirdly, Strep-Tactin is already used in clinically relevant settings, where no adverse responses have been reported^38^. Furthermore, it was shown that doses up to 150 µg of streptavidin do not induce negative effects in patients^49^. The reported value is several folds higher than any anticipated Expamer burden after short-term cell cultures. Lastly, D-biotin is a naturally synthetized coenzyme in humans denominated as vitamin B_7_ (also known as vitamin H) and therefore not suspected to produce toxic effects at the concentration used. Although it has to be confirmed in clinical trials, we are confident that Expamers and/or Expamer components can be used in the clinically relevant T cell cultures. As of now, Expamers are produced under GMP guidelines and a Drug Master File (DMF; #019173) has been filed.

In conclusion, Expamers are cross-linking activation reagents that open new possibilities for T cell stimulation. Solubility, ease-of-use, temporal control over the activation signal, reversibility, modular nature, and predicted safety make Expamers an attractive alternative to other T cell activation reagents. Continuous improvements of T cell activation agents are important for developing immunotherapies and as a research tool for understanding the underlying mechanism of T cell activation.

## Materials and methods

### Blood samples

Fresh PBMCs were generated from either buffy coats by centrifugation over Biocoll separating solution or from Leukapheresis material. Buffy coats were obtained from autologous adult male or female blood donors at the Institute for Anesthesiology, German Heart Centre Munich (State of Bavaria and Technical University Munich). Written informed consent was obtained from the donors, and usage of the blood samples was approved according to national law by the local Institutional Review Board and the declaration of Helsinki and Istanbul (Ethics committee of the Faculty of Medicine, Technical University of Munich: 360/13 and 55/14). Leukapheresis products of healthy donors’ material were collected at CCC Cellex Collection Center Dresden under the ethical quote (Ethical committee of the Technical University Dresden: EK309072016).

### Expamers preparation

Strep-Tactin multimer backbone is generated by chemical coupling of differentially activated SAm2-tetramer (streptavidin mutein 2). Coupling is based on the reaction of reactive thiol groups of iminothiolane activated SAm2 with maleimide activated SAm2. A defined amount and ratio of differentially activated SAm2-tetramer is combined and allowed to react (couple) for a defined period. The coupling reaction is stopped by the addition of *N*-ethylmaleimide blocking available thiol functions. For removal of residual N-substituted iminothiolane, hydroxylamine is added. Quenching reagents and non-reacted SAm2-tetramers are removed using Size Exclusion Chromatography (SEC). Pooling of the target elution fractions is performed according to minimum absorption requirements of the main elution fraction. The obtained Strep-Tactin multimer bulk is 0.2 µm filtrated prior to storage.

Monomeric anti-CD3 and anti-CD28 Fab fragments originating from monoclonal antibodies were generated as described before^30^. Briefly, the chimeric heavy and the light chain of Fab fragments were expressed in the periplasm of E.coli K-12 allowing disulfide bond formation, protein folding and assembly of the Fab heterodimers. Fab fragments were purified by Strep-tag:Strep-Tactin affinity chromatography followed by Hydrophobic Interaction Chromatography (HIC) and stored in PBS pH 7.5.

Expamers were compounded in a proprietary buffer by mixing given amounts of anti-CD3 and anti-CD28 Fab fragments together with Strep-Tactin multimer backbone prior to use.

### Expamers characterization

Shape and size distribution of Strep-Tactin multimer backbone was determined using dynamic light scattering (DLS). DLS enables the measurement of the diffusion behavior of particles in solution that can be used to calculate the hydrodynamic radius by the Stokes–Einstein equation. The calculated hydrodynamic radius corresponds to the hydrodynamic radius of a solid sphere with the same diffusion behavior as the measured molecule. The DLS measurement was performed using DynaPro NanoStar detector (Wyatt). 2 mg/mL of Strep-Tactin multimer backbone solution was diluted 1:50 in formulation buffer. 10 µL of the dilution was loaded into a capped disposable microcuvette (Wyatt) and measured under specified settings (300 acquisitions, 25 °C, aqueous solvent). Data was collected using DYNAMICS Software (Wyatt).

Expamers shape and size was further visualized using negative-stain electron microscopy (EM) imaging. Slotted copper grids GD100/400 (Polaron) were coated with 80 nm of amorphous carbon prepared on a carbon evaporator. To freshly glow-discharged (Harrick Scientific Corporation) carbon-coated grids 3.5 µL of Expamers at a final concentration of 0.18 mg/mL were applied. After 30 s of incubation, excess sample was blotted away followed by three wash steps with deionized water before staining for 15 s with 2% uranyl acetate. EM screening was performed on a CM 200 microscope (Philips) equipped with a 4 k × 4 k TemCam-F416 (TVIPS) and on a Tecnai F20 (FEI) equipped with a 2 k × 2 k Eagle (FEI). Images were recorded at a nominal magnification of 66,000 or 50,000 fold, corresponding to 1.61 Å/px and 2.21 Å/px at the specimen level.

### T cell:Expamers interactions

To measure Expamers cell-binding kinetics, freshly isolated T cells were resuspended in FACS buffer (PBS from Thermo Fischer Scientific with 0.5% BSA from Carl Roth) and measured continuously using a CytoFLEX Flow Cytometer (Beckman Coulter). During the measurement pre-assembled anti-CD3 and anti-CD28 Fab fragments with fluorescent Strep-Tactin PE (IBA Lifesciences) were added to T cells. Increase in signal intensity was recorded.

Changes in intracellular Ca^2+^ were monitored using a flow cytometer LSRI analyzer (BD Biosciences). Cells were stained with Calcium Sensor Dye eFluor514 (eBiosciences) according to manufacturer’s instructions and illuminated with 325 nm laser line of a helium-cadmium laser. Fluorescence emissions from 390 to 420 nm and from 500 to 520 nm were detected simultaneously, and changes in the ratio of the two emission intensities were analyzed.

Residual content of individual Expamers components on T cell surfaces was measured using flow cytometric detection after 8 days of culture. For Fab fragment detection, T cells were stained with fluorescent Strep-Tactin PE (IBA Lifesciences) that should be able to bind to the Strep-tag of remaining Fabs. For detection of Strep-Tactin multimer backbone DyLight488 anti-streptavidin antibody (Vector Laboratories) was used.

### T cell stimulation and culture

T cells were positively enriched using LS columns and CD4/CD8 MicroBeads according to the manufacturer’s protocol (Miltenyi Biotec). Isolated T cells were cultured in serum-free or serum-containing media in the presence or absence of cytokines. Subsequently, T cells were stimulated with either Expamers or Dynabeads Magnetic Beads (Thermo Fisher Scientific) according to in-house S.O.P. or manufacturer’s recommendations, respectively. Activated T cells were kept in culture for at least 7 days unless indicated otherwise.

For antigen-specific activation, Strep-tagged pMHCs were produced as described (Knabel et al., 2002). To generate pMHC/CD28 Expamers, Streptagged pMHC (HLA-A*02:01/pp65495-503) and anti-CD28 Fab fragment were added to the Strep-Tactin multimer backbone and complexed for 15 min at RT. Subsequently, pMHC/CD28 Expamers were added to PBMCs of an HLA-A*02:01^+^ CMV seropositive donor. Cells were checked before and after 8 days in vitro cell culture for the presence of HLA-A*02:01/pp65495-503 specific T cells by flow cytometry staining with fluorescently labeled pMHC multimer and anti-CD8 (PE; clone HIT8a) antibody.

### In vitro characterization

To measure change in early metabolic state of T cells, WST-1 assay was used. The metabolic switch in activated T cells results in an increase of mitochondrial dehydrogenase activity, which in turn refers to the amount of formazan dye produced from WST-1 reagent. To assess T cell activation levels 100 µL cell suspension (~ 5 × 10^5^ cells) was transferred into a 96 well plate and 10 µL of WST-1 reagent (Roche) was added. Cells were incubated for 4 h at 37 °C and absorbance of formazan produced from WST-1 dye was measured at 450 nm in an ELISA Reader (Aglient Technologies).

For Western Blot analysis human primary T cells were serum-starved overnight in RPMI (Gibco) containing 2% FCS and activated with either monoclonal anti-CD3 antibodies (clone OKT3, Biolegend) according to manufacturer’s recommendations or with in-house reagents. Subsequently, cells lysed on ice using standard lysis buffer (150 mM NaCl, 0.5% NP40, 50 mM Hepes, pH7.6, 1 mM DTT, 1 mM Na_2_EDTA, 1 mM EGTA, 20 mM β-glycerolphosphate, Na_3_VO_4_, 0.4 mM PMSF, 1 mM NaF) supplemented with a protease inhibitor cocktail (Roche). Extracts were supplemented with 5 × Laemmli loading dye, incubated at 95 °C for 5 min, separated by SDS-PAGE, transferred onto nitrocellulose membranes and analyzed via immunoblot. Immunoblot antibodies against phosphorylated ZAP70 (Tyr493) and GAPDH (14C10) as well as HRP conjugated secondary antibodies were purchased from Cell Signaling. HRP-conjugated anti-Strep-Tag antibody was obtained from IBA.

Nur77 upregulation was measured using an in-house generated Jurkat T cell line that carried the tdTomato reporter gene downstream of Nur77 locus. Intensity of reporter gene upregulation was assessed by flow cytometry.

Cell cycle study was carried out as previously described^50^. Briefly, 1 × 10^6^ cultured T cells were fixed in 1 mL ice cold 70% ethanol for 30 min at − 20 °C, then washed and stained in 0.5 mL PI staining solution (PBS supplemented with 100 µg/mL RNase A and 50 µg/mL propidium iodide). Samples were kept in the dark for 1 h at room temperature prior to flow cytometric analysis.

T cells were stained with CFSE (Thermo Fisher Scientific) according to manufacturer’s protocol. Afterwards, the cells were stimulated with Expamers or Dynabeads (Thermo Fisher Scientific) for 7 days for T cell expansion according to in-house S.O.P. or manufacturer’s recommendation, respectively. Proliferation was assessed using flow cytometric analysis and live nucleated cells were enumerated using the cell analyzer NucleoCounter® NC-3000^TM^ (ChemoMetec) after staining with Solution 13 (ChemoMetec) according to the manufacturer’s protocol.

### T cell phenotyping

To determine T cell activation state, human T cells were stimulated as described above. After 24 h, T cells were stained with either antibodies against CD25 (BV650; clone BC96) and CD69 (APC-Fire750; clone FN50) (Biolegend). To characterize cell phenotype after activation and 7-day culture T cells were stained with the following antibodies: anti-CD45 (AF700; clone HI300), anti-CD3 (PE, FITC or PE-Cy7; clone OKT3), anti-CD8 (PE or BV510; clone HIT8a), anti-CD4 (BV421 or BV785; clone OKT4), anti-CD27 (BV785; clone 323), anti-CD45RO (BV510; clone UCHL1), anti-CD28 (APC-Fire750; clone CD28.2), anti-CD45RA (PE; clone HI100), anti-CCR7 (FITC; G043H7), anti-CD62L (BV650; clone DREG-56), anti-CD57 (AF647; clone HNK-1), anti-PD1 (PE; clone HE12.2H7), anti-TIM3 (PE-Cy7; clone F38-2E2) (all from Biolegend), and anti-LAG3 (eFluor450; clone 3DS223H; Thermo Fisher Scientific). For live/dead discrimination propidium iodide (Sigma) was used. Cell associated fluorescence was analyzed by flow cytometry using either CytoFLEX Flow Cytometer or CytoFLEX LX Flow Cytometer (Beckman Coulter), unless indicated otherwise.

RNASeq reads were mapped to the human genome (GRCh38) and aligned to the GENCODE release 24 gene model using the OSA aligner and Array Studio software (Omicsoft Corporation). RNAseq quality metrics were generated as part of the pipeline and evaluated for consistency across samples. Lists of differentially expressed genes were generated in R (version 3.4) using the DESeq2 package (version 1.16.1) by comparison of Expamer-treated samples to Dynabeads controls, using a model taking donor and treatment conditions into account. Prior to DESeq analysis, the gene set was filtered for protein-coding genes, excluding genes with zero counts across all samples. Differentially expressed genes were selected by imposing a log_2_FC cutoff of 1 and Benjamini–Hochberg adjusted FDR cutoff of 0.1.

### T cell in vitro function analysis

For in vitro killing assay, xCELLigence RTCA System (ACEA Biosciences Inc.) was used. To measure specific lysis, 2 × 10^4^ CD19 presenting Human Embryonic Kidney cells (HEK293-CD19^+^) were seeded into E-Plate 96 (ACEA Biosciences Inc.). After resting, CAR-positive cells were added in a 5:1 E:T ratio and incubated for indicated time while recording changes in impedance signal with RTCA Software Pro (ACEA Biosciences Inc.).

For investigation of on-target cytokine secretion CAR T cells were surface-stained for presence of CD3, CD4, CD8 (antibodies listed above) as well as CAR receptors using Erbitux and/or anti-CAR antibodies (Juno Therapeutics Inc.). Subsequently, an intracellular cytokine staining procedure was performed using Fixation/Permeabilization Solution Kit (BD Biosciences) in accordance to manufacturer’s manual. IL-2, IFNg, and TNFa were detected using anti-IL-2 (PE; clone MQ1.17H12; eBiosciences), anti-IFNg (FITC; clone 4S.B3; Biolegend) as well as anti-TNFa (APC; clone Mab11; eBiosciences) antibodies, respectively. For live/dead discrimination, Ethidium Monoazide Bromide (EMA) from Thermo Fisher Scientific was used.

To evaluate B cell clearance potential of CAR T cells, CTV dilution assay was used. 5 × 10^6^ CAR T cells were labeled with 1 µM CellTrace Violet (CTV) dye (Thermo Fisher Scientific) according to the manufacturer’s instructions. Labeled cells were co-cultured with 2.5 × 10^6^ CD19^+^ purified B cells from a healthy donor and proliferation/activation status of CAR T cells were monitored in a time course. At the indicated time points, co-cultured cells were stained with anti-CAR (Juno Therapeutics Inc.), anti-CD3, anti-CD19 (BV421, clone HIB19), anti-CD25 (all from BioLegend) antibodies and CTV dilution as well as CD25 expression of CAR^+^ cells were assessed by flow cytometry.

### In vivo tumor mouse model

NOD.Cg-Prkdcscid Il2rgtmWjlTg (CMV-IL3, CSF2, KITLG) 1Eav/MloySzJ (NSGS, The Jackson Laboratory) mice were bred in a pathogen free facility at the Technical University of Munich according to standard practices under an approved protocol. All experiments were performed in accordance with the guidelines of the Institutional Animal Care and Use Committee of the Regierung von Oberbayern. The animal experimental protocols were also approved by Institutional Animal Care and Use Committee of the Regierung von Oberbayern (ROB-55.2-2532.Vet_02-17-138 and Vet_02-18-162).

To evaluate CAR T cell function in vivo, 6–8-week-old male NSGS mice were selected and bred in-house. First, tumor cells were injected via tail vein with 5 × 10^5^ CD19 + Raji/ffluc cells. 7 days later, mice were additionally injected intravenously with PBS (control) or 0.75 × 10^6^ selected CAR T cells from three different healthy donors. Bioluminescence imaging was performed similar to previously described^51^. Briefly, mice received intraperitoneal injections of luciferin substrate (XenoLight d-Luciferin, Perkin Elmer) resuspended in PBS (15 μg/g body weight), anesthetized with isoflurane and imaged using an IVIS Lumina Imaging System (Perkin Elmer) 10 min after luciferin injection (in small binning mode and with an acquisition time up to 1 min to obtain unsaturated images). Luciferase activity was analyzed using Living Image Software (Perkin Elmer). All mice were monitored for survival, imaged, weight and bled once a week. Red blood cells were lysed using Tris-Buffered Ammonium Chloride lysis buffer (ACT). Lymphocytes were counted (Sysmex), stained with fluorochrome labeled antibodies (listed above) and analyzed for frequencies and phenotype by flow cytometry.

### Data analysis and statistics

Flow cytometric data was analyzed using FlowJo software (FlowJo, LLC). Graphs and statistical analysis were generated using GraphPad Prism software (GraphPad Software). RNA sequencing data was analyzed and visualized using TIBCO Spotfire software (TIBCO Software Inc.). Principle component analysis were performed on data set collected from T cell phenotypic analysis by flow cytometry (based of frequency gating and geoMFIs of each channel) using JMP software (SAS Institute Inc.).

### Supplementary information

Supplementary Information.

Supplementary Table S1.

## Data Availability

The authors declare that all data generated or analyzed for this study are available within the paper and its Supplementary Information. Additional raw data are available from the corresponding author upon reasonable request. Expamers are proprietary reagents of Bristol-Myers Squibb Company and information about precise formulation are considered trade secret. Specific information about Expamers can be made available upon reasonable request.
