# Supplementary material for: Expamers: a new technology to control T cell activation
Source: Sci Rep. 2020 Oct 20;10:17832. doi: 10.1038/s41598-020-74595-8 (PMC7575567; doi:10.1038/s41598-020-74595-8)
Supplement: Supplementary file 1 — Supplementary Information. [file 41598_2020_74595_MOESM1_ESM.pdf]

Supplementary Information 1

Expamers – A New Technology to Control T Cell Activation

Mateusz P. Poltorak[#*1], Patricia Graef[#1], Claudia Tschulik[1], Michaela Wagner[1], Vlad Cletiu[1], Stefan Dreher[1], Bojana Borjan[1], Simon P. Fraessle[1,2], Manuel Effenberger[1,2], Martin Turk[3], Dirk H. Busch[2], Juergen Plitzko[3], David Kugler[4], Seamus Ragan[4], Thomas Schmidt[1], Christian Stemberger[§1], Lothar Germeroth[§1]

Affiliations

[1] Juno Therapeutics GmbH, a Bristol-Myers Squibb Company, Grillparzerstr. 10, 81675 Munich, Germany

[2] Institute for Medical Microbiology Immunology and Hygiene, Technical University of Munich, Munich, Germany

[3] Max Planck Institute of Biochemistry, Am Klopferspitz 18, 82152 Martinsried, Germany

[4] Juno Therapeutics Inc., a Bristol-Myers Squibb Company, 400 Dexter Avenue North, Suite 1200, Seattle, WA 98109

[#] first author

[§] last author

* corresponding author

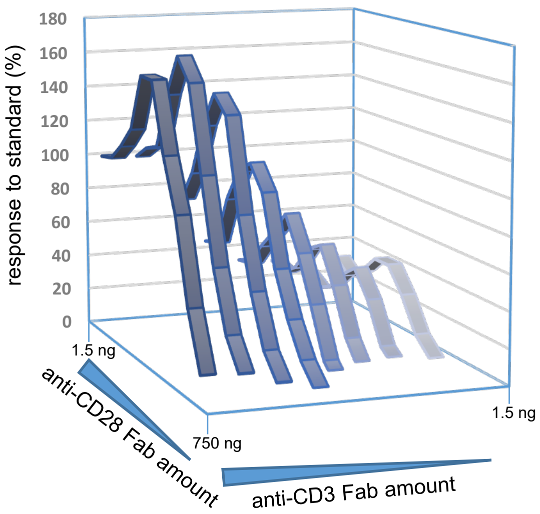

Supplementary Figure 1. anti-CD3 and anti-CD28 Fab fragment amount titration using WST-1 assay. Graph depicts changes in WST-1 reagent as % of standard (Expamers formulation) dependent on content of anti-CD3 and anti-CD28 Fab fragments (in the ranges from 1.5 to 750 ng) in Expamers formulation.

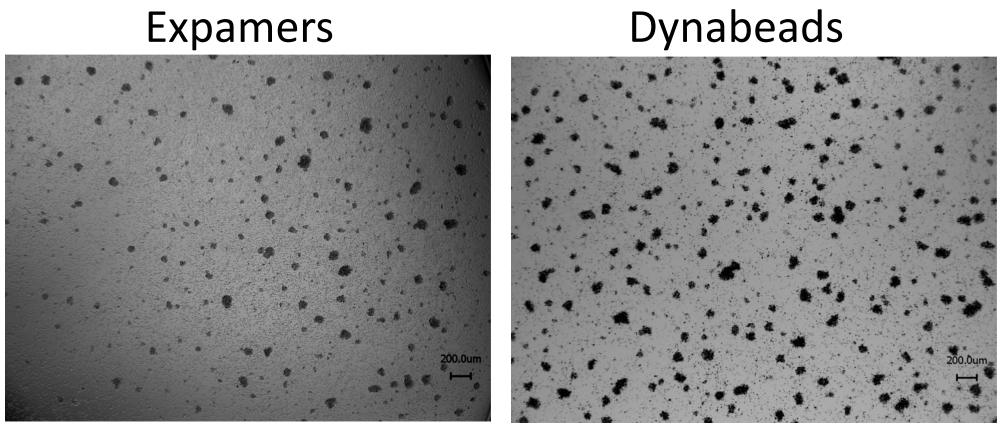

Supplementary Figure 2. Microscopic analysis visualizing the activation-induced cluster formation. Images show human primary T cells aggregation at day 3 post activation with either Expamers or Dynabeads. Black bars indicate a scale of 200 µm.

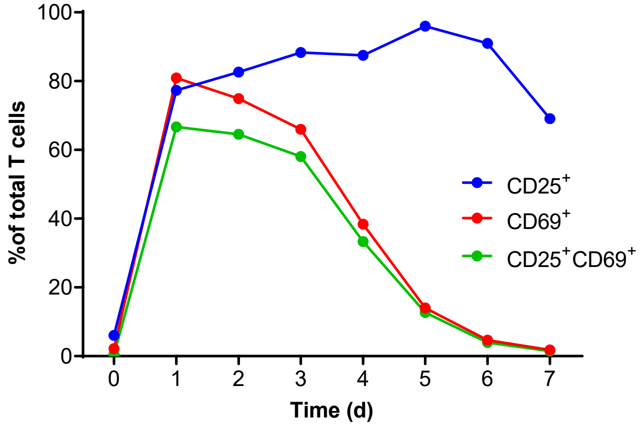

Supplementary Figure 3. Expression kinetics of CD69 and CD25 activation markers upon Expamers stimulation. CD69 and CD25 expression over time in human primary T cells was detected using flow cytometry. Graph presents changes in activation markers expression level during indicated time period. Cells were pre-gated of live, single $CD3^+$ lymphocytes.

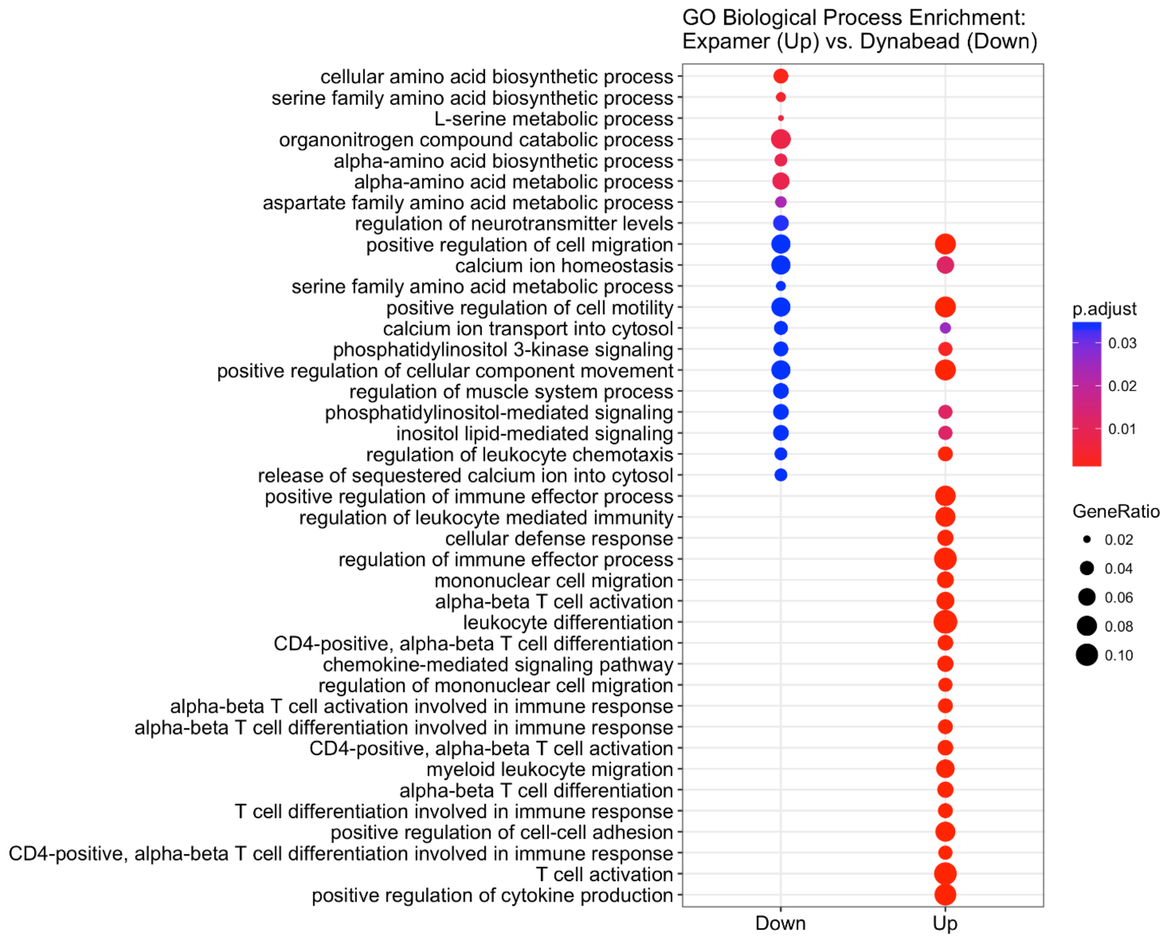

Supplementary Figure 4. Differential regulation of gene clusters between Expamers and Dynabeads. Differentially regulated genes (based of $\log_2$FC cutoff of 1) were tested for differential regulation in T cell activation-related pathways. Graph shows the top 20 enriched GO Biological Process terms with a Benjamini-Hochberg adjusted p-value ≤ 0.1 where points are colored by adjusted p-value and sized by the ratio of genes in the group (up or downregulated) relative to genes in the term (gene ratio).

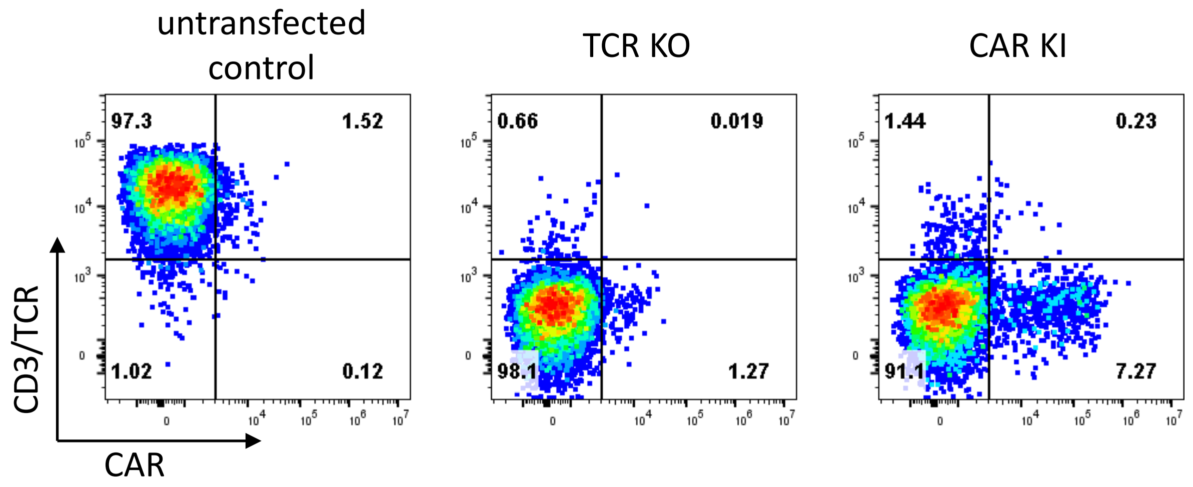

Supplementary Figure 5. Use of Expamers for electroporation and non-viral gene delivery. Human primary T cells were nucleofected using Lonza electroporator in the presence or absence of Cas9 protein loaded with gRNA against TRAC locus and/or CAR HDR DNA template. Gene editing efficiency was assessed using flow cytometry 5 days after electroporation. Dot plots from one representative experiment are shown.

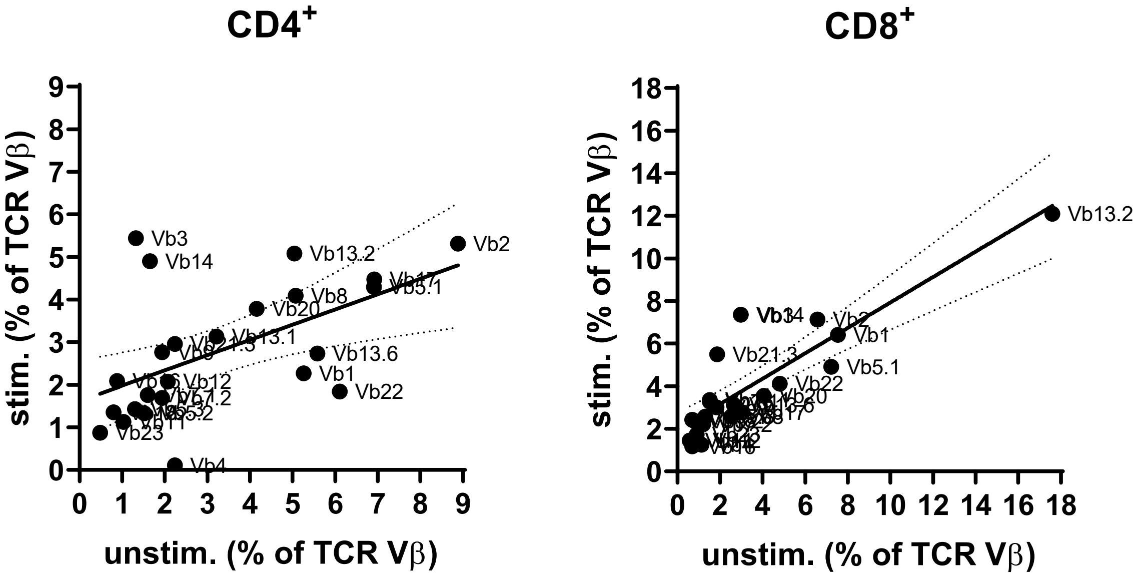

Supplementary Figure 6. The diversity assessment of the TCR Vβ repertoire upon 7-day stimulation with Expamers. Human primary T cells were tested for expression of different TCR Vβ chains prior to activation and 7 days after. Graphs depict comparative analysis of stimulated vs. unstimulated conditions for CD4 and CD8 T cells from one representative out of three independent experiments.

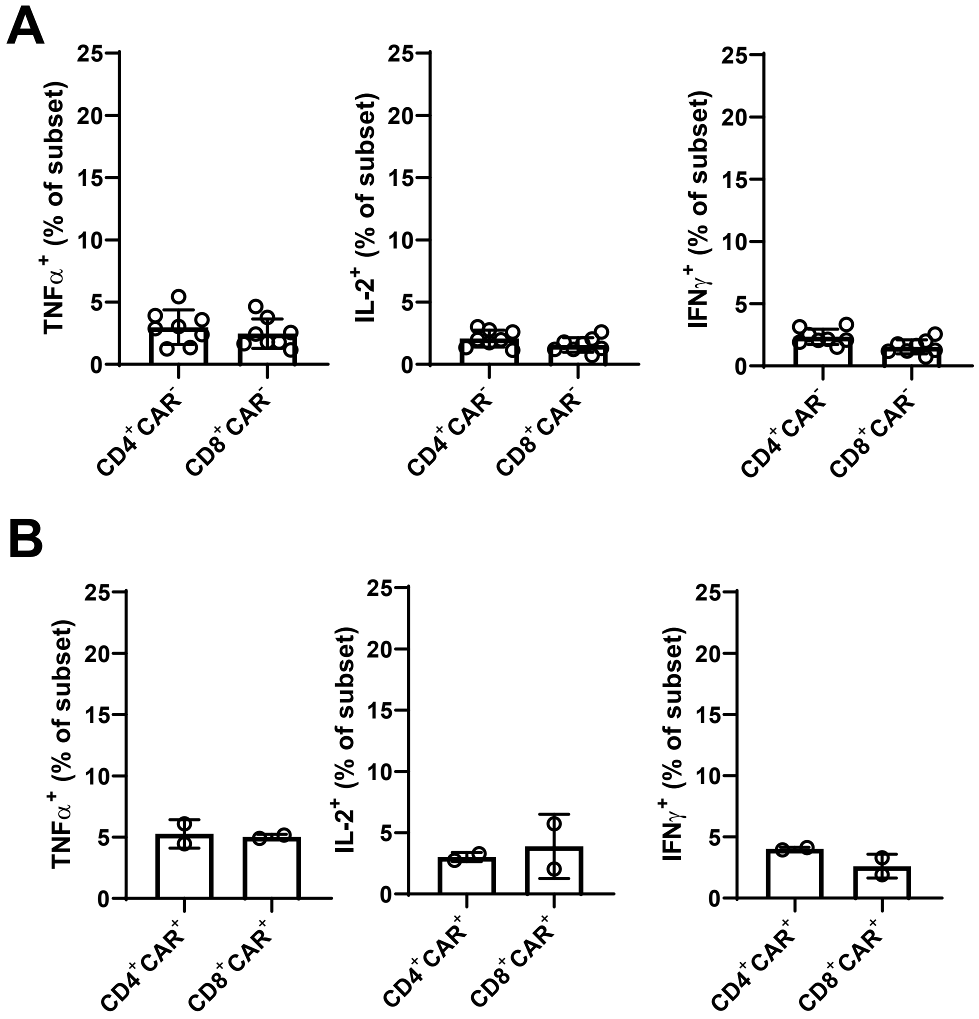

Supplementary Figure 7. Assessment of off-target killing of CAR T cells in co-culture system. (A) Graphs show CAR⁻ T cells co-cultured under similar conditions as CAR⁺ T cells from Figure 5D and tested for secretion of cytokines using intracelular cytokine staining. **(B)** Graphs show CAR⁺ T cells from Figure 5D co-cultured with CD19⁻ HEK cells and tested for cytokine production. All cells were pre-gated on live, single, $CD3^{+}CAR^{+/-}$ cells. Error bars represent mean ± SD.

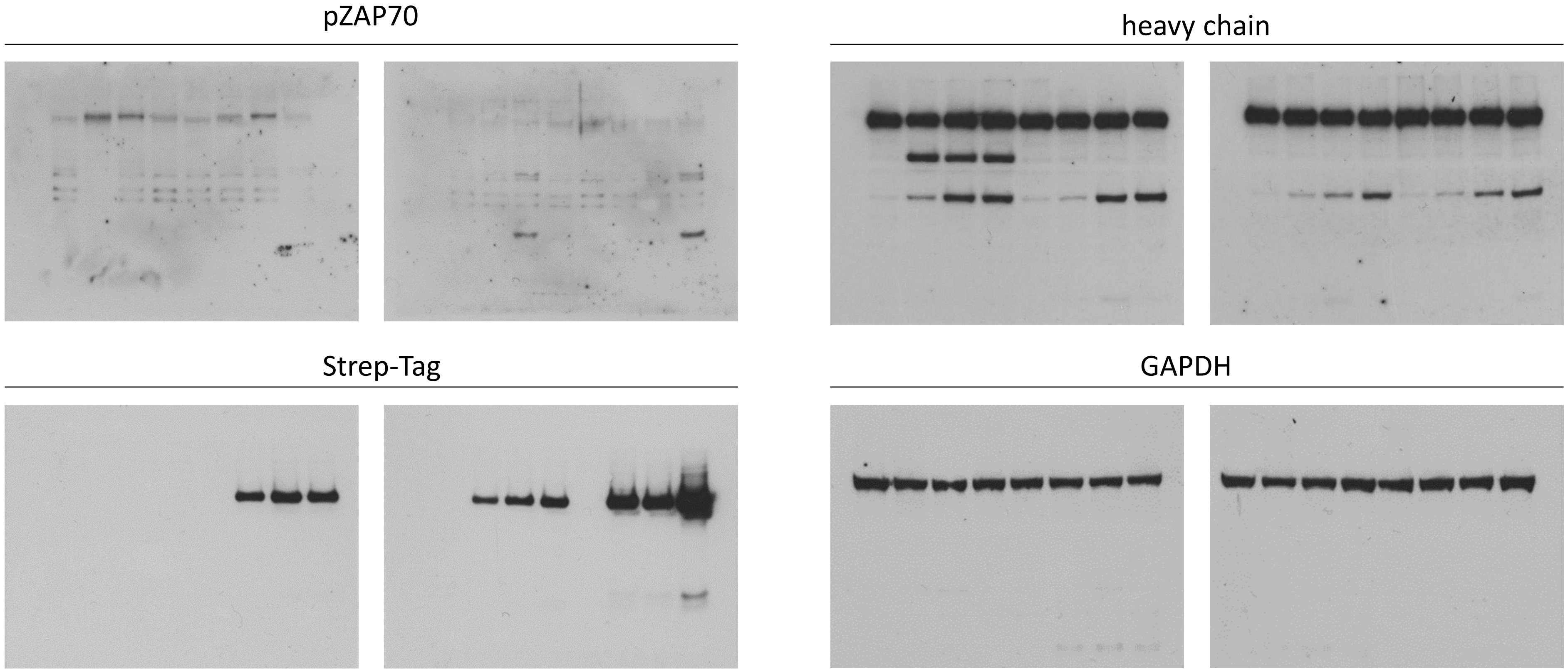

Supplementary Figure 8. Full-lenght blots of phosphorylation kinetics upon Expamers stimulation. Displayed are full images from the same representative experiment as in Figure 2B.

Supplementary Materials & Methods

WST-1 assay measurement, flow cytometry stainings as well as Western blot acquisition were performed as described in “Materials & Methods”.

Widefield microscopic images were acquired using Axio Vert.A1 microscope and Axiocam 105 color camera using ZENcore software (all from Zeiss) at 50x magnification. Full images are displayed.

TCR Vβ repertoire profiling was performed using Beta Mark TCR Vbeta Repertoire Kit (Beckman Coulter) according to manufacturer’s protocol. Cell associated fluorescence was analyzed by flow cytometry using CytoFLEX Flow Cytometer (Beckman Coulter).

Non-viral gene delivery was performed as previously described[6]. Briefly, T cells were activated for 2 days with Expamers in the presence of IL-2, IL-7 and IL-15. Afterward, cells were electroporated with Cas9 ribonucleoprotein loaded with gRNA targeting TRAC locus (Integrated DNA Technologies) and CAR HDR DNA template in Nucleofector Solution (Lonza) with a 4D Nucleofector X unit (Lonza). After electroporation, cells were further cultured in RPMI medium containing IL-2 before analysis at day 7.

Genes differentially expressed under the separate activation conditions were tested for enrichment in GO Biological Process terms using the R Bioconductor clusterProfiler package.

For Supplementary Table 1, all Expamers conditions were collapsed and treated as technical replicates for each donor. Differential expression analysis was performed in R using the DESeq2 package and yielded 500+ genes with $|\log_2 FC| \geq 1$ and Benjamini-Hochberg adjusted p-value ≤ 0.1.
