# Supplementary material for: Expamers: a new technology to control T cell activation
Source: Sci Rep. 2020 Oct 20;10:17832. doi: 10.1038/s41598-020-74595-8 (PMC7575567; doi:10.1038/s41598-020-74595-8)
Supplement: Supplementary file 2 — Supplementary Table S1. [file 41598_2020_74595_MOESM2_ESM.pdf]

GeneID	Dynabeads	baseMean	log2FoldChange	lfcSE	stat	pvalue	padj	GeneName	GeneDescription	EntrezID	neg.log10padj	Regulation.Direction	Expamers
ENSG00000121807.5	893,8381542	4326,266888	2,509195823	0,06830113	36,73725212	1,86E-295	3,06E-291	CCR2	chemokine (C-C motif) receptor 2	729230	290,5148703	Upregulated	5184,374072
ENSG00000111796.3	171,8606164	2280,431839	3,813804551	0,106153054	35,92741254	1,14E-282	9,38E-279	KLRB1	killer cell lectin-like receptor subfamily B, member 1	3820	278,027831	Upregulated	2807,574645
ENSG00000115956.9	281,7229522	3514,849467	3,664044697	0,119183846	30,74279619	1,53E-207	8,37E-204	PLEK	pleckstrin	5341	203,0773654	Upregulated	4323,131096
ENSG00000126822.16	636,59815	2533,943419	2,190856148	0,073869465	29,65848122	2,64E-193	1,08E-189	PLEKHG3	pleckstrin homology domain containing, family G (with RhoGef domain) member 3	26030	188,9649002	Upregulated	3008,279737
ENSG00000113088.5	408,0200475	2262,243984	2,631436561	0,089963665	29,24999289	4,49E-188	1,48E-184	GZMK	granzyme K (granzyme 3; tryptase II)	3003	183,8304948	Upregulated	2725,799968
ENSG00000100450.12	192,8465176	3224,353267	3,90556013	0,133689072	29,2137574	1,30E-187	3,56E-184	GZMH	granzyme H (cathepsin G-like 2, protein h-CCPX)	2999	183,4491206	Upregulated	3982,229954
ENSG00000150938.9	35,93199057	735,4794196	4,006341654	0,15907078	25,18590559	5,72E-140	1,34E-136	CRIM1	cysteine rich transmembrane BMP regulator 1 (chordin-like)	51232	135,8718591	Upregulated	910,3662768
ENSG00000172215.5	493,7070147	1913,700179	2,129504917	0,084771863	25,12042134	2,98E-139	6,12E-136	CXCR6	chemokine (C-X-C motif) receptor 6	10663	135,2133818	Upregulated	2268,69847
ENSG00000112486.14	10,21070449	647,1186758	4,727005345	0,190944262	24,7559434	2,68E-135	4,89E-132	CCR6	chemokine (C-C motif) receptor 6	1235	131,3107234	Upregulated	806,3456686
ENSG00000164398.12	2215,263336	965,1824918	-1,734807549	0,072028944	-24,08486719	3,60E-128	5,92E-125	ACSL6	acyl-CoA synthetase long-chain family member 6	23305	124,2273878	Downregulated	652,6622807
ENSG00000160791.13	929,285683	6825,531347	2,862764518	0,122809465	23,31061786	3,46E-120	5,17E-117	CCR5	chemokine (C-C motif) receptor 5 (gene/pseudogene)	1234	116,2862218	Upregulated	8299,592763
ENSG00000000971.15	48,37936082	453,6341886	3,157176902	0,143580461	21,98890357	3,68E-107	5,04E-104	CFH	complement factor H	3075	103,297496	Upregulated	554,9478955
ENSG00000135362.13	196,9161175	1084,127638	2,52480948	0,115034667	21,94824872	9,00E-107	1,14E-103	PRR5L	proline rich 5 like	79899	102,9435766	Upregulated	1305,930518
ENSG00000159640.15	1022,004168	286,1277581	-3,055122958	0,139889064	-21,83961259	9,76E-106	1,15E-102	ACE	angiotensin I converting enzyme	1636	101,9406581	Downregulated	102,1586554
ENSG00000275302.1	65,76178155	711,0610103	3,277045466	0,152863967	21,43765814	5,95E-102	6,53E-99	CCL4	chemokine (C-C motif) ligand 4	6351	98,1852048	Upregulated	872,3858175
ENSG00000180739.13	19,18159059	401,4110913	3,816831021	0,179454543	21,26906876	2,20E-100	2,26E-97	S1PR5	sphingosine-1-phosphate receptor 5	53637	96,64638102	Upregulated	496,9684665
ENSG00000130589.16	234,6179142	868,5955375	2,041444343	0,098235015	20,781229	6,40E-96	6,19E-93	HELZ2	helicase with zinc finger 2, transcriptional coactivator	85441	92,20816115	Upregulated	1027,089943
ENSG00000142634.12	2275,074177	6046,974125	1,569850162	0,077126701	20,35417222	4,26E-92	3,90E-89	EFHD2	EF-hand domain family, member D2	79180	88,40935054	Upregulated	6989,949112
ENSG00000155629.14	197,5429976	813,2722968	2,165881571	0,107025973	20,23697151	4,63E-91	4,01E-88	PIK3AP1	phosphoinositide-3-kinase adaptor protein 1	118788	87,39729876	Upregulated	967,2046217
ENSG00000175274.18	29,52369187	448,5981756	3,505694944	0,174097174	20,13642644	3,54E-90	2,91E-87	TP53I11	tumor protein p53 inducible protein 11	9537	86,53594675	Upregulated	553,3667965
ENSG00000232653.8	38,54302415	487,487071	3,317754058	0,167079496	19,85733817	9,52E-88	7,46E-85	GOLGA8N	golgin A8 family, member N	643699	84,12735259	Upregulated	599,7230827
ENSG00000104972.15	24,75380248	609,6701834	3,716697963	0,192081116	19,34962711	2,05E-83	1,54E-80	LILRB1	leukocyte immunoglobulin-like receptor, subfamily B (with TM and ITIM domains), member 1	10859	79,81387368	Upregulated	755,8992786
ENSG00000169252.5	125,0616775	815,0110583	2,64616814	0,13725546	19,27914667	8,04E-83	5,75E-80	ADRB2	adrenoceptor beta 2, surface	154	79,24040323	Upregulated	987,4984034
ENSG00000221869.4	23,14915747	317,9125408	3,394324753	0,17691636	19,18604226	4,84E-82	3,32E-79	CEBPD	CCAAT/enhancer binding protein (C/EBP), delta	1052	78,47913063	Upregulated	391,6033866
ENSG00000158321.15	181,852573	1181,831314	2,640777037	0,138161608	19,11368198	1,94E-81	1,28E-78	AUTS2	autism susceptibility candidate 2	26053	77,89342994	Upregulated	1431,826
ENSG00000162630.5	39,69035073	409,1161217	3,161784564	0,165884358	19,06017304	5,41E-81	3,42E-78	B3GALT2	UDP-Gal:betaGlcNAc beta 1,3-galactosyltransferase, polypeptide 2	8707	77,46569817	Upregulated	501,4725645
ENSG00000067141.16	106,9694261	493,569369	2,29434503	0,121334345	18,90927932	9,57E-80	5,83E-77	NEO1	neogenin 1	4756	76,23454265	Upregulated	590,2193547
ENSG00000139187.9	206,2173258	1161,365308	2,475413899	0,132171112	18,72885726	2,88E-78	1,69E-75	KLRG1	killer cell lectin-like receptor subfamily G, member 1	10219	74,77160375	Upregulated	1400,152304
ENSG00000163823.3	27,3354723	441,9044134	3,435432818	0,18583006	18,48695966	2,63E-76	1,49E-73	CCR1	chemokine (C-C motif) receptor 1	1230	72,82638008	Upregulated	545,5466487
ENSG00000168229.3	13,84280852	332,6251914	3,587543951	0,197542877	18,16083678	1,05E-73	5,78E-71	PTGDR	prostaglandin D2 receptor (DP)	5729	70,23814372	Upregulated	412,3207872
ENSG00000062524.15	31,80880381	446,4003891	3,283997271	0,184254538	17,82315547	4,67E-71	2,48E-68	LTK	leukocyte receptor tyrosine kinase	4058	67,60569997	Upregulated	550,0482854
ENSG00000168329.13	177,3403365	3772,068915	3,508300191	0,199123538	17,61871158	1,77E-69	9,10E-67	CX3CR1	chemokine (C-X3-C motif) receptor 1	1524	66,04108782	Upregulated	4670,75106
ENSG00000114166.7	1814,955274	3995,842006	1,301302814	0,074215175	17,53418784	7,86E-69	3,92E-66	KAT2B	K(lysine) acetyltransferase 2B	8850	65,4071769	Upregulated	4541,063689
ENSG00000185697.16	210,5152148	895,5107401	2,170288903	0,124857458	17,38213264	1,13E-67	5,45E-65	MYBL1	v-myb avian myeloblastosis viral oncogene homolog-like 1	4603	64,26350383	Upregulated	1066,759621
ENSG00000044115.20	624,7255533	1295,041779	1,206673072	0,070598862	17,09196198	1,70E-65	8,01E-63	CTNNA1	catenin (cadherin-associated protein), alpha 1, 102kDa	1495	62,09662581	Upregulated	1462,620835
ENSG00000101665.8	99,57382128	461,0032231	2,251737426	0,132540071	16,98910681	9,89E-65	4,52E-62	SMAD7	SMAD family member 7	4092	61,34506564	Upregulated	551,3605736
ENSG00000203747.9	100,0355403	794,0178358	2,820053573	0,166356329	16,9518863	1,86E-64	8,29E-62	FCGR3A	Fc fragment of IgG, low affinity IIIa, receptor (CD16a)	2214	61,08169655	Upregulated	967,5134097
ENSG00000148848.14	21,50560749	298,9810003	3,211315367	0,189607292	16,936666	2,41E-64	1,04E-61	ADAM12	ADAM metallopeptidase domain 12	8038	60,9808877	Upregulated	368,3498485
ENSG00000205336.11	319,6346957	1572,169813	2,201868547	0,132395029	16,63105146	4,15E-62	1,75E-59	ADGRG1	NA	9289	58,7566494	Upregulated	1885,303592
ENSG00000115607.9	72,48132001	348,8864443	2,301322985	0,140263448	16,40714683	1,70E-60	6,99E-58	IL18RAP	interleukin 18 receptor accessory protein	8807	57,15547432	Upregulated	417,9877254
ENSG00000135318.11	2242,098924	907,9591571	-1,868882519	0,114055683	-16,38570271	2,42E-60	9,71E-58	NT5E	5'-nucleotidase, ecto (CD73)	4907	57,01293338	Downregulated	574,4242153
ENSG00000089692.8	292,5810994	1001,516664	1,840470052	0,112883561	16,30414592	9,22E-60	3,61E-57	LAG3	lymphocyte-activation gene 3	3902	56,44231597	Upregulated	1178,750555
ENSG00000124256.14	172,162315	671,9577466	1,997205725	0,123136405	16,21945776	3,67E-59	1,41E-56	ZBP1	Z-DNA binding protein 1	81030	55,85218766	Upregulated	796,9066044
ENSG00000156475.18	78,55929652	517,4540429	2,53561766	0,158431797	16,00447454	1,19E-57	4,45E-55	PPP2R2B	protein phosphatase 2, regulatory subunit B, beta	5521	54,35211055	Upregulated	627,1777295
ENSG00000023902.13	631,3129548	1660,254044	1,520954289	0,098955696	15,37005296	2,60E-53	9,50E-51	PLEKHO1	pleckstrin homology domain containing, family O member 1	51177	50,02219858	Upregulated	1917,489316
ENSG00000198846.5	164,859202	720,1042527	2,083297063	0,135833615	15,33712445	4,32E-53	1,54E-50	TOX	thymocyte selection-associated high mobility group box	9760	49,81125362	Upregulated	858,9155154
ENSG00000134545.13	208,1376111	656,7688766	1,780399108	0,117265297	15,18265977	4,61E-52	1,61E-49	KLRC1	killer cell lectin-like receptor subfamily C, member 1	3821	48,7925528	Upregulated	768,926693
ENSG00000178573.6	1453,467011	4319,347906	1,717954684	0,114972617	14,94229432	1,75E-50	5,99E-48	MAF	v-maf avian musculoaponeurotic fibrosarcoma oncogene homolog	4094	47,22246264	Upregulated	5035,818129
ENSG00000007264.13	253,8523515	1380,427783	2,329134168	0,156560774	14,8768693	4,66E-50	1,56E-47	MATK	megakaryocyte-associated tyrosine kinase	4145	46,80589172	Upregulated	1662,071641
ENSG00000150687.11	36,83634611	324,359649	2,695594752	0,182672456	14,75643789	2,80E-49	9,20E-47	PRSS23	protease, serine, 23	11098	46,03621614	Upregulated	396,2404747
ENSG00000204161.13	759,2000133	1557,055324	1,176399523	0,079762232	14,74882911	3,13E-49	1,01E-46	C10orf128	chromosome 10 open reading frame 128	170371	45,99584499	Upregulated	1756,519152
ENSG00000178860.8	244,3867222	804,5771316	1,878507953	0,12837707	14,63273736	1,74E-48	5,49E-46	MSC	musculin	9242	45,26019727	Upregulated	944,6247339
ENSG00000211689.7	1026,79018	2383,26675	1,392111509	0,095383473	14,59489226	3,03E-48	9,39E-46	TRGC1	T cell receptor gamma constant 1	445347	45,02716394	Upregulated	2722,385893
ENSG00000144290.16	87,63221566	389,9198524	2,138434415	0,14804824	14,44417317	2,73E-47	8,31E-45	SLC4A10	solute carrier family 4, sodium bicarbonate transporter, member 10	57282	44,08041808	Upregulated	465,4917616
ENSG00000197872.11	38,69864404	260,5483004	2,488641152	0,172431166	14,43266442	3,22E-47	9,64E-45	FAM49A	family with sequence similarity 49, member A	81553	44,01587816	Upregulated	316,0107145
ENSG00000158470.5	886,8242783	1953,379552	1,27292246	0,088634212	14,36152509	9,02E-47	2,65E-44	B4GALT5	UDP-Gal:betaGlcNAc beta 1,4- galactosyltransferase, polypeptide 5	9334	43,57677351	Upregulated	2220,018371
ENSG00000196116.7	204,6126808	550,4457274	1,550240746	0,109467849	14,16160782	1,58E-45	4,57E-43	TDRD7	tudor domain containing 7	23424	42,34019927	Upregulated	636,903989
ENSG00000196586.13	38,18333821	299,1336706	2,680291553	0,190070935	14,10153298	3,72E-45	1,05E-42	MYO6	myosin VI	4646	41,97722912	Upregulated	364,3712538
ENSG00000080493.13	7,833100737	146,8140774	3,039180596	0,215563258	14,09878765	3,86E-45	1,08E-42	SLC4A4	solute carrier family 4 (sodium bicarbonate cotransporter), member 4	8671	41,96775804	Upregulated	181,5593216
ENSG00000182557.7	678,4730599	274,9904746	-1,806837126	0,128684507	-14,04082873	8,77E-45	2,40E-42	SPNS3	spinster homolog 3 (Drosophila)	201305	41,61913141	Downregulated	174,1198283
ENSG00000181045.14	381,8656661	941,2063769	1,428749212	0,102119445	13,9909614	1,77E-44	4,77E-42	SLC26A11	solute carrier family 26 (anion exchanger), member 11	284129	41,32123643	Upregulated	1081,041555
ENSG00000254087.7	114,7636219	411,3246803	1,887914881	0,135029702	13,98147854	2,02E-44	5,37E-42	LYN	v-yes-1 Yamaguchi sarcoma viral related oncogene homolog	4067	41,27040654	Upregulated	485,4649449
ENSG00000035664.11	344,5624604	94,9718487	-2,79753198	0,204082615	-13,70784072	9,11E-43	2,38E-40	DAPK2	death-associated protein kinase 2	23604	39,6235677	Downregulated	32,57419579
ENSG00000188389.10	32,43128334	206,5228992	2,437776193	0,182094154	13,38744896	7,16E-41	1,81E-38	PDCD1	programmed cell death 1	5133	37,74189977	Upregulated	250,0458032
ENSG00000105339.10	257,8500222	706,5710525	1,60134789	0,119843475	13,3619948	1,01E-40	2,51E-38	DENND3	DENN/MADD domain containing 3	22898	37,5998606	Upregulated	818,7513101
ENSG00000004399.12	83,82026865	319,208554	1,955609483	0,146616583	13,33825578	1,39E-40	3,40E-38	PLXND1	plexin D1	23129	37,46799151	Upregulated	378,0556254
ENSG00000152217.16	24,36475278	198,3618286	2,580874161	0,193912153	13,30950185	2,04E-40	4,93E-38	SETBP1	SET binding protein 1	26040	37,30711438	Upregulated	241,8610975
ENSG00000140678.16	782,3154065	207,9518535	-2,908610662	0,218588377	-13,30633723	2,13E-40	5,07E-38	ITGAX	integrin, alpha X (complement component 3 receptor 4 subunit)	3687	37,29506232	Downregulated	64,36096531
ENSG00000105374.9	1744,016211	12058,10833	2,455248486	0,18512992	13,26229971	3,83E-40	9,00E-38	NKG7	natural killer cell granule protein 7	4818	37,04582156	Upregulated	14636,63136
ENSG00000134539.16	596,7037332	4109,34087	2,408543141	0,182229363	13,21709684	6,99E-40	1,62E-37	KLRD1	killer cell lectin-like receptor subfamily D, member 1	3824	36,79060227	Upregulated	4987,500154
ENSG00000174032.16	1110,0724	583,2536921	-1,257594036	0,096806305	-12,99082779	1,38E-38	3,15E-36	SLC25A30	solute carrier family 25, member 30	253512	35,50156977	Downregulated	451,5490151
ENSG00000184613.10	10208,39796	6025,030298	-1,034430381	0,080104392	-12,91352898	3,78E-38	8,51E-36	NELL2	NEL-like 2 (chicken)	4753	35,07018788	Downregulated	4979,188384
ENSG00000162496.8	30,32087371	204,1573664	2,401934831	0,186163531	12,90228444	4,37E-38	9,71E-36	DHRS3	dehydrogenase/reductase (SDR family) member 3	9249	35,01268781	Upregulated	247,6164896
ENSG00000198133.8	250,7311527	536,8175431	1,227715273	0,096234085	12,75759283	2,83E-37	6,12E-35	TMEM229B	transmembrane protein 229B	161145	34,21321204	Upregulated	608,3391407
ENSG00000149294.16	27,1945343	193,7930987	2,469505918	0,194770055	12,67908417	7,72E-37	1,65E-34	NCAM1	neural cell adhesion molecule 1	4684	33,78259759	Upregulated	235,4427398
ENSG00000143185.3	30,8655433	184,4509401	2,331211247	0,184835509	12,61235604	1,81E-36	3,81E-34	XCL2	chemokine (C motif) ligand 2	6846	33,41946894	Upregulated	222,8472894
ENSG00000109861.15	4611,866899	8764,228807	1,051084423	0,083611361	12,57107187	3,05E-36	6,34E-34	CTSC	cathepsin C	1075	33,19783199	Upregulated	9802,319284
ENSG00000197943.9	170,2904759	472,8577443	1,532321512	0,122034388	12,55647315	3,66E-36	7,53E-34	PLCG2	phospholipase C, gamma 2 (phosphatidylinositol-specific)	5336	33,12314033	Upregulated	548,4995614
ENSG00000163508.12	304,9888245	2285,564516	2,413302774	0,192884905	12,51162069	6,45E-36	1,31E-33	EOMES	eomesodermin	8320	32,88284752	Upregulated	2780,708439
ENSG00000151208.16	37,03087097	186,8503002	2,175581822	0,174464226	12,47007405	1,09E-35	2,15E-33	DLG5	discs, large homolog 5 (Drosophila)	9231	32,66663571	Upregulated	224,3051575
ENSG00000067082.14	4113,656449	7607,959032	1,010054642	0,081570363	12,38261795	3,25E-35	6,36E-33	KLF6	Kruppel-like factor 6	1316	32,19684492	Upregulated	8481,534678
ENSG00000157404.15	18,55911106	141,3622804	2,45757568	0,199229341	12,33541037	5,84E-35	1,13E-32	KIT	v-kit Hardy-Zuckerman 4 feline sarcoma viral oncogene homolog	3815	31,94696234	Upregulated	172,0630727
ENSG00000170113.15	520,6482966	960,8835286	1,010328424	0,08241304	12,25932728	1,50E-34	2,86E-32	NIPA1	non imprinted in Prader-Willi/Angelman syndrome 1	123606	31,54305395	Upregulated	1070,942337
ENSG00000035720.7	696,0771489	304,9723728	-1,669810346	0,138343269	-12,07005124	1,52E-33	2,87E-31	STAP1	signal transducing adaptor family member 1	26228	30,54144944	Downregulated	207,1961787
ENSG00000116667.12	304,3714857	839,7549182	1,498316205	0,1243142	12,05265528	1,88E-33	3,51E-31	C1orf21	chromosome 1 open reading frame 21	81563	30,45467162	Upregulated	973,6007763
ENSG00000211695.2	42,40341734	236,4445618	2,200327016	0,183011641	12,02288013	2,69E-33	4,98E-31	TRGV9	T cell receptor gamma variable 9	NA	30,30285649	Upregulated	284,9548479
ENSG00000181847.11	722,2330105	1443,042569	1,101677551	0,091704499	12,01334243	3,02E-33	5,53E-31	TIGIT	T cell immunoreceptor with Ig and ITIM domains	201633	30,25758791	Upregulated	1623,244959
ENSG00000221866.9	19,20581368	141,5978427	2,397142572	0,201221376	11,91296184	1,01E-32	1,81E-30	PLXNA4	plexin A4	91584	29,74200785	Upregulated	172,19585
ENSG00000075234.16	126,5885126	503,9210785	1,854536099	0,156986852	11,81332115	3,33E-32	5,89E-30	TTC38	tetratricopeptide repeat domain 38	55020	29,22974688	Upregulated	598,25422
ENSG00000136603.13	126,9335167	341,1019011	1,513374083	0,12977786	11,66126555	2,01E-31	3,48E-29	SKIL	SKI-like oncogene	6498	28,45834549	Upregulated	394,6439973
ENSG00000106069.20	39,60299957	176,7806208	2,008297827	0,173223356	11,59368963	4,44E-31	7,45E-29	CHN2	chimerin 2	101928168;1124	28,12811851	Upregulated	211,0750261
ENSG00000167851.13	938,6265364	1934,075277	1,140588506	0,098763426	11,54869317	7,50E-31	1,23E-28	CD300A	CD300a molecule	11314	27,91040575	Upregulated	2182,937462
ENSG00000118402.5	84,20931836	252,9814039	1,64881731	0,142778296	11,54809492	7,55E-31	1,23E-28	ELOVL4	ELOVL fatty acid elongase 4	6785	27,91040575	Upregulated	295,1744252
ENSG00000117560.7	178,6242005	574,2546798	1,668306334	0,145140098	11,49445509	1,41E-30	2,27E-28	FASLG	Fas ligand (TNF superfamily, member 6)	356	27,64429836	Upregulated	673,1622996
ENSG00000211821.2	15,90917251	220,501424	2,534760769	0,22127831	11,45508013	2,22E-30	3,54E-28	TRDV2	T cell receptor delta variable 2	NA	27,45084468	Upregulated	271,6494868
ENSG00000133067.17	5,752054868	115,1967315	2,637852467	0,230418049	11,44811562	2,40E-30	3,80E-28	LGR6	leucine-rich repeat containing G protein-coupled receptor 6	59352	27,4201435	Upregulated	142,5579006
ENSG00000153814.11	192,3745172	465,53183	1,362309235	0,119048529	11,44331015	2,54E-30	3,98E-28	JAZF1	JAZF zinc finger 1	221895	27,40023272	Upregulated	533,8211582
ENSG00000079112.9	1,05997542	81,91716002	2,745014265	0,241016555	11,38931831	4,73E-30	7,33E-28	CDH17	cadherin 17, LI cadherin (liver-intestine)	1015	27,13463187	Upregulated	102,1314562
ENSG00000138166.5	108,6225173	305,8803409	1,568777942	0,137898181	11,37634976	5,48E-30	8,43E-28	DUSP5	dual specificity phosphatase 5	1847	27,07411232	Upregulated	355,1947968
ENSG00000189233.11	468,7887912	882,8931672	1,00504822	0,088860509	11,31040364	1,17E-29	1,78E-27	NUGGC	nuclear GTPase, germinal center associated	389643	26,75079055	Upregulated	986,4192612
ENSG00000164342.12	11,83957259	102,4016854	2,422763852	0,214491547	11,29538149	1,38E-29	2,09E-27	TLR3	toll-like receptor 3	7098	26,68048431	Upregulated	125,0422136
ENSG00000129667.12	555,0439109	1036,749494	1,013939998	0,089788624	11,29252185	1,43E-29	2,14E-27	RHBDF2	rhomboid 5 homolog 2 (Drosophila)	79651	26,67031592	Upregulated	1157,17589
ENSG00000135114.12	608,0338808	1527,049195	1,355307031	0,121029523	11,19815234	4,16E-29	6,11E-27	OASL	2'-5'-oligoadenylate synthetase-like	8638	26,21367151	Upregulated	1756,803024
ENSG00000080573.6	4,497554595	80,90391083	2,594014419	0,231690814	11,19601754	4,26E-29	6,21E-27	COL5A3	collagen, type V, alpha 3	50509	26,20706925	Upregulated	100,0054999
ENSG00000181104.6	1048,027893	3306,30181	1,561603198	0,141347202	11,04799509	2,24E-28	3,21E-26	F2R	coagulation factor II (thrombin) receptor	2149	25,49401656	Upregulated	3870,870289
ENSG00000095397.13	81,36485496	223,694411	1,535947318	0,139381686	11,01972122	3,07E-28	4,35E-26	DFNB31	deafness, autosomal recessive 31	25861	25,36119461	Upregulated	259,2768
ENSG00000162383.11	1,05997542	64,08162007	2,643417561	0,240984473	10,96924433	5,37E-28	7,55E-26	SLC1A7	solute carrier family 1 (glutamate transporter), member 7	6512	25,12194106	Upregulated	79,83703123
ENSG00000080546.13	560,7372382	1098,945118	1,091228858	0,100003515	10,911905	1,01E-27	1,39E-25	SESN1	sestrin 1	27244	24,85825294	Upregulated	1233,497088
ENSG00000272908.1	28,92543543	152,9743329	2,119373933	0,194597015	10,8910917	1,27E-27	1,73E-25	RP11-121A8.1	NA	NA	24,76250159	Upregulated	183,9865572
ENSG00000173706.12	299,3292615	637,0160076	1,188560109	0,109621546	10,84239502	2,17E-27	2,90E-25	HEG1	heart development protein with EGF-like domains 1	57493	24,53788929	Upregulated	721,4376941
ENSG00000136854.17	912,1029278	435,446379	-1,44470279	0,133847312	-10,79366309	3,69E-27	4,85E-25	STXBP1	syntaxin binding protein 1	6812	24,31401705	Downregulated	316,2822417
ENSG00000163421.8	25,34691826	129,7830825	2,086663446	0,194048896	10,75328688	5,72E-27	7,47E-25	PROK2	prokineticin 2	60675	24,1269622	Upregulated	155,8921236
ENSG00000148488.15	16,75039998	123,3126093	2,24896136	0,209877644	10,71558321	8,60E-27	1,11E-24	ST8SIA6	ST8 alpha-N-acetyl-neuraminide alpha-2,8-sialyltransferase 6	338596	23,95312438	Upregulated	149,9531617
ENSG00000173930.8	43,18151675	229,1913633	2,030948331	0,189661009	10,70830713	9,30E-27	1,20E-24	SLCO4C1	solute carrier organic anion transporter family, member 4C1	353189	23,92239123	Upregulated	275,6938249
ENSG00000092621.11	1001,326587	495,1156165	-1,319709318	0,123885879	-10,65262105	1,70E-26	2,16E-24	PHGDH	phosphoglycerate dehydrogenase	26227	23,66524667	Downregulated	368,562874
ENSG00000171813.13	37,56085868	160,5986893	1,941731256	0,182371833	10,64710062	1,80E-26	2,28E-24	PWWP2B	PWWP domain containing 2B	170394	23,64284607	Upregulated	191,358147
ENSG00000099204.18	9748,750474	5518,979102	-1,084424347	0,102313324	-10,59905305	3,01E-26	3,75E-24	ABLIM1	actin binding LIM protein 1	3983	23,42587612	Downregulated	4461,536259
ENSG00000089012.14	1603,051241	745,341586	-1,51772784	0,144428172	-10,50853041	7,89E-26	9,54E-24	SIRPG	signal-regulatory protein gamma	55423	23,02027379	Downregulated	530,9141721
ENSG00000125735.10	276,2241497	763,0539254	1,524950519	0,145259141	10,49813808	8,81E-26	1,06E-23	TNFSF14	tumor necrosis factor (ligand) superfamily, member 14	8740	22,97562818	Upregulated	884,7613693
ENSG00000115523.16	2840,890244	9862,659148	1,812632866	0,173242695	10,46296853	1,28E-25	1,50E-23	GNLY	granulysin	10578	22,82352435	Upregulated	11618,10137
ENSG00000108924.13	2,003235928	61,01713658	2,49734943	0,240226134	10,3958274	2,59E-25	3,00E-23	HLF	hepatic leukemia factor	3131	22,52282693	Upregulated	75,77061174
ENSG00000276070.4	19,63376836	121,2427704	2,156944326	0,207573806	10,39121633	2,72E-25	3,13E-23	CCL4L2	chemokine (C-C motif) ligand 4-like 2	388372;9560	22,50487167	Upregulated	146,6450209
ENSG00000100298.15	185,4604539	601,6366986	1,626457137	0,15697808	10,36104616	3,73E-25	4,20E-23	APOBEC3H	apolipoprotein B mRNA editing enzyme, catalytic polypeptide-like 3H	164668	22,37669242	Upregulated	705,6807597
ENSG00000173482.16	147,9003354	346,8973333	1,327612201	0,12888291	10,30091735	6,98E-25	7,76E-23	PTPRM	protein tyrosine phosphatase, receptor type, M	5797	22,11033963	Upregulated	396,6465828
ENSG00000161405.16	3037,01292	7124,879087	1,25344072	0,121904906	10,28211877	8,48E-25	9,37E-23	IKZF3	IKAROS family zinc finger 3 (Aiolos)	22806	22,02846405	Upregulated	8146,845629
ENSG00000243836.5	353,310198	158,5450964	-1,549187492	0,150679666	-10,28133078	8,55E-25	9,38E-23	WDR86-AS1	WDR86 antisense RNA 1	100131176	22,02781775	Downregulated	109,853821
ENSG00000101842.13	1490,576392	806,9182727	-1,175270451	0,114762696	-10,2408752	1,30E-24	1,41E-22	VSIG1	V-set and immunoglobulin domain containing 1	340547	21,85160505	Downregulated	636,0037429
ENSG00000049249.8	74,59172964	320,0553467	1,934168524	0,189353482	10,2145918	1,71E-24	1,82E-22	TNFRSF9	tumor necrosis factor receptor superfamily, member 9	3604	21,73982048	Upregulated	381,4212509
ENSG00000188011.5	2,64993855	70,31449416	2,455142002	0,240369121	10,21404909	1,72E-24	1,82E-22	RTP5	receptor (chemosensory) transporter protein 5 (putative)	285093	21,73982048	Upregulated	87,23063306
ENSG00000272282.1	20,99030166	154,0015162	2,163449369	0,212085337	10,20084368	1,97E-24	2,07E-22	RP11-222K16.2	NA	NA	21,68352176	Upregulated	187,2543198
ENSG00000170962.12	3,063211348	74,58857937	2,443237178	0,23973638	10,19134926	2,17E-24	2,27E-22	PDGFD	platelet derived growth factor D	80310	21,64385753	Upregulated	92,46992138
ENSG00000050730.15	9,953051577	81,79111497	2,269093084	0,22303127	10,17387868	2,59E-24	2,70E-22	TNIP3	TNFAIP3 interacting protein 3	79931	21,56862421	Upregulated	99,75063081
ENSG00000006468.13	19,94500812	113,2217135	2,076089874	0,204304594	10,16173858	2,94E-24	3,04E-22	ETV1	ets variant 1	2115	21,51724675	Upregulated	136,5408898
ENSG00000120833.13	125,9366693	378,1489899	1,556050729	0,153595716	10,13082116	4,03E-24	4,15E-22	SOCS2	suppressor of cytokine signaling 2	8835	21,38243403	Upregulated	441,2020701
ENSG00000168685.14	56983,29485	30724,37274	-1,166702227	0,115210831	-10,12667139	4,21E-24	4,30E-22	IL7R	interleukin 7 receptor	3575	21,36671102	Downregulated	24159,64221
ENSG00000111728.10	35,02763504	132,7270347	1,82307208	0,180601757	10,09443157	5,85E-24	5,90E-22	ST8SIA1	ST8 alpha-N-acetyl-neuraminide alpha-2,8-sialyltransferase 1	6489	21,22915014	Upregulated	157,1518846
ENSG00000270055.1	75,52030827	213,4658568	1,527414045	0,151834042	10,05976017	8,32E-24	8,34E-22	CTD-3092A11.2	NA	NA	21,07860335	Upregulated	247,952244
ENSG00000107679.14	256,0941579	596,4327905	1,285274922	0,127852166	10,05282087	8,93E-24	8,90E-22	PLEKHA1	pleckstrin homology domain containing, family A (phosphoinositide binding specific) member 1	59338	21,05064278	Upregulated	681,5174486
ENSG00000011600.11	9,189634043	105,0552219	2,294844455	0,228571287	10,03995072	1,02E-23	1,01E-21	TYROBP	TYRO protein tyrosine kinase binding protein	7305	20,99656745	Upregulated	129,0216188
ENSG00000137309.19	9690,125244	5557,0895	-1,058003776	0,105430164	-10,03511457	1,07E-23	1,05E-21	HMGA1	high mobility group AT-hook 1	3159	20,9804814	Downregulated	4523,830564
ENSG00000232810.3	116,7910809	456,9550012	1,795719876	0,180691891	9,938021403	2,84E-23	2,74E-21	TNF	tumor necrosis factor	7124	20,56292265	Upregulated	541,9959813
ENSG00000117461.14	209,5528719	573,6785777	1,422791605	0,143336657	9,926222882	3,20E-23	3,06E-21	PIK3R3	phosphoinositide-3-kinase, regulatory subunit 3 (gamma)	8503	20,51405653	Upregulated	664,7100042
ENSG00000171860.4	176,3339479	393,3376672	1,246238895	0,125605029	9,921886934	3,34E-23	3,18E-21	C3AR1	complement component 3a receptor 1	719	20,49770034	Upregulated	447,588597
ENSG00000180644.6	5337,040526	13761,60881	1,35141248	0,136362279	9,910456847	3,75E-23	3,54E-21	PRF1	perforin 1 (pore forming protein)	5551	20,45048847	Upregulated	15867,75089
ENSG00000183625.14	20,79577681	96,15837708	1,944308422	0,197363485	9,851409058	6,76E-23	6,32E-21	CCR3	chemokine (C-C motif) receptor 3	1232	20,19951928	Upregulated	114,9990271
ENSG00000115604.10	296,2609095	554,9366202	1,01492108	0,103279825	9,826905489	8,62E-23	8,01E-21	IL18R1	interleukin 18 receptor 1	8809	20,09621358	Upregulated	619,6055479
ENSG00000277632.1	33,92875465	225,6566565	2,144165016	0,218371191	9,818900573	9,34E-23	8,53E-21	CCL3	chemokine (C-C motif) ligand 3	6348	20,06901665	Upregulated	273,588632
ENSG00000148411.7	216,7590926	439,7779429	1,15254553	0,117392648	9,817868079	9,43E-23	8,57E-21	NACC2	NACC family member 2, BEN and BTB (POZ) domain containing	138151	20,06697533	Upregulated	495,5326555
ENSG00000104154.6	87,79737675	220,5274008	1,394906622	0,142120763	9,814938992	9,71E-23	8,77E-21	SLC30A4	solute carrier family 30 (zinc transporter), member 4	7782	20,0567538	Upregulated	253,7099068
ENSG00000175029.16	18,2236482	106,3657926	2,066944834	0,210995398	9,796160757	1,17E-22	1,05E-20	CTBP2	C-terminal binding protein 2	1488	19,97835122	Upregulated	128,4013287
ENSG00000159618.15	847,638952	1750,50134	1,105654129	0,112942116	9,789564476	1,25E-22	1,12E-20	ADGRG5	NA	221188	19,95237734	Upregulated	1976,216937
ENSG00000139679.15	3681,886027	7685,384911	1,163189154	0,118956058	9,778309528	1,40E-22	1,24E-20	LPAR6	lysophosphatidic acid receptor 6	10161	19,90641807	Upregulated	8686,259632
ENSG00000100628.11	1849,511689	1112,603522	-1,023217812	0,105468072	-9,701683091	2,97E-22	2,61E-20	ASB2	ankyrin repeat and SOCS box containing 2	51676	19,58360789	Downregulated	928,3764808
ENSG00000171451.13	8,465121479	69,0579245	2,179792418	0,225932762	9,647969583	5,01E-22	4,36E-20	DSEL	dermatan sulfate epimerase-like	92126	19,36017657	Upregulated	84,20612525
ENSG00000204131.8	312,1994457	934,3166674	1,495610432	0,155603005	9,611706636	7,14E-22	6,18E-20	NHSL2	NHS-like 2	340527	19,20920808	Upregulated	1089,845973
ENSG00000102962.4	23,57197148	129,9440658	2,060070336	0,214616598	9,598839773	8,08E-22	6,96E-20	CCL22	chemokine (C-C motif) ligand 22	6367	19,15724373	Upregulated	156,5370894
ENSG00000188001.9	496,5375363	261,9491292	-1,203873063	0,125502047	-9,592457588	8,60E-22	7,37E-20	TPRG1	tumor protein p63 regulated 1	285386	19,13263197	Downregulated	203,3020274
ENSG00000025708.13	322,9115143	638,8715292	1,104250402	0,115533052	9,557874455	1,20E-21	1,02E-19	TYMP	thymidine phosphorylase	1890	18,98954003	Upregulated	717,8615329
ENSG00000108798.8	795,0835577	1501,919757	1,005032022	0,105185183	9,554882127	1,24E-21	1,05E-19	ABI3	ABI family, member 3	51225	18,97923228	Upregulated	1678,628806

ENSG00000182985.16	78,62756525	235,4003198	1,626978217	0,17044715	9,545353029	1,36E-21	1,14E-19	CADM1	cell adhesion molecule 1	23705	18,94151831	Upregulated	274,5935084
ENSG00000000938.12	31,45865907	499,3161672	2,261430514	0,237769063	9,511037662	1,89E-21	1,58E-19	FGR	feline Gardner-Rasheed sarcoma viral oncogene homolog	2268	18,80242008	Upregulated	616,2805442
ENSG00000085563.14	169,4103434	357,4612952	1,160055321	0,122013036	9,507634226	1,95E-21	1,62E-19	ABCB1	ATP-binding cassette, sub-family B (MDR/TAP), member 1	5243	18,79041118	Upregulated	404,4740332
ENSG00000164949.7	6,087517724	81,47906266	2,245449816	0,236335646	9,501105127	2,08E-21	1,72E-19	GEM	GTP binding protein overexpressed in skeletal muscle	2669	18,76535686	Upregulated	100,3269489
ENSG00000147883.10	3,063211348	115,6734601	2,310712709	0,244718418	9,442332657	3,65E-21	2,97E-19	CDKN2B	cyclin-dependent kinase inhibitor 2B (p15, inhibits CDK4)	1030	18,52745585	Upregulated	143,8260223
ENSG00000243772.7	15,17511873	90,909007	2,064558737	0,218695534	9,440333336	3,72E-21	3,01E-19	KIR2DL3	killer cell immunoglobulin-like receptor, two domains, long cytoplasmic tail, 3	3804	18,52131266	Upregulated	109,8424791
ENSG00000170837.2	23,7958601	101,1415389	1,85740788	0,19699749	9,428586546	4,16E-21	3,35E-19	GPR27	G protein-coupled receptor 27	2850	18,47478705	Upregulated	120,4779586
ENSG00000073350.13	360,1603931	863,8098397	1,285537339	0,136742155	9,401177968	5,40E-21	4,33E-19	LLGL2	lethal giant larvae homolog 2 (Drosophila)	3993	18,36360441	Upregulated	989,7222013
ENSG00000110987.8	570,6946904	268,39379	-1,443908943	0,153642091	-9,39787352	5,57E-21	4,45E-19	BCL7A	B-cell CLL/lymphoma 7A	605	18,35207907	Downregulated	192,8185649
ENSG00000221957.8	16,94492484	96,61730341	2,005395516	0,21383881	9,378070883	6,72E-21	5,34E-19	KIR2DS4	killer cell immunoglobulin-like receptor, two domains, short cytoplasmic tail, 4	3809	18,2725476	Upregulated	116,5353981
ENSG00000130830.14	515,0915067	296,6470691	-1,039659075	0,110945812	-9,370872639	7,19E-21	5,66E-19	MPP1	membrane protein, palmitoylated 1, 55kDa	4354	18,24709118	Downregulated	242,0359597
ENSG00000187210.12	187,8865039	594,0553582	1,55655728	0,166119626	9,370098626	7,25E-21	5,68E-19	GCNT1	glucosaminyl (N-acetyl) transferase 1, core 2	2650	18,2459792	Upregulated	695,5975718
ENSG00000204472.12	571,8530384	259,9905366	-1,506341179	0,160787024	-9,368549405	7,35E-21	5,73E-19	AIF1	allograft inflammatory factor 1	199	18,24166825	Downregulated	182,0249112
ENSG00000182463.15	537,4192592	247,0626874	-1,444414325	0,154224561	-9,365656883	7,56E-21	5,86E-19	TSHZ2	teashirt zinc finger homeobox 2	128553	18,23182343	Downregulated	174,4735445
ENSG00000136161.12	3337,396976	6398,060609	1,048102951	0,111985213	9,35929772	8,03E-21	6,20E-19	RCBTB2	regulator of chromosome condensation (RCC1) and BTB (POZ) domain containing protein 2	1102	18,20772175	Upregulated	7163,226517
ENSG00000159733.13	473,2900062	943,2454068	1,079702573	0,115672369	9,334144223	1,02E-20	7,83E-19	ZFYVE28	zinc finger, FYVE domain containing 28	57732	18,1065087	Upregulated	1060,734257
ENSG00000143365.16	130,7263813	1172,556888	2,137568799	0,229183564	9,326885217	1,09E-20	8,34E-19	RORC	RAR-related orphan receptor C	6097	18,07878798	Upregulated	1433,014515
ENSG00000128604.18	136,6435972	352,8902447	1,393205167	0,151584588	9,190942067	3,89E-20	2,92E-18	IRF5	interferon regulatory factor 5	3663	17,53391747	Upregulated	406,9519066
ENSG00000122547.10	339,2295591	164,0712618	-1,319543623	0,144227946	-9,14901486	5,75E-20	4,26E-18	EEPD1	endonuclease/exonuclease/phosphatase family domain containing 1	80820	17,37091139	Downregulated	120,2816875
ENSG00000106701.11	94,74080383	230,1943709	1,341006695	0,146970287	9,124338812	7,22E-20	5,25E-18	FSD1L	fibronectin type III and SPRY domain containing 1-like	83856	17,27960571	Upregulated	264,0577627
ENSG00000138378.17	1127,970126	2173,601376	1,037945303	0,113997999	9,104943155	8,63E-20	6,23E-18	STAT4	signal transducer and activator of transcription 4	6775	17,20575246	Upregulated	2435,009188
ENSG00000049768.14	41,86388842	271,5938262	1,930301515	0,213882364	9,025061619	1,80E-19	1,28E-17	FOXP3	forkhead box P3	50943	16,89320641	Upregulated	329,0263106
ENSG00000189430.12	7,599670913	66,16120205	2,081954618	0,23118626	9,005529215	2,15E-19	1,52E-17	NCR1	natural cytotoxicity triggering receptor 1	9437	16,81768824	Upregulated	80,80158484
ENSG00000235531.9	80,95158216	229,4588341	1,450668292	0,16134457	8,991119394	2,45E-19	1,72E-17	MSC-AS1	NA	100132891	16,76442437	Upregulated	266,5856471
ENSG00000139193.3	5588,113762	3109,184426	-1,138328647	0,126961311	-8,965949054	3,08E-19	2,14E-17	CD27	CD27 molecule	939	16,6687841	Downregulated	2489,452092
ENSG00000171956.6	174,5641418	52,24655124	-2,149898773	0,240072987	-8,955188178	3,39E-19	2,33E-17	FOXB1	forkhead box B1	27023	16,63188447	Downregulated	21,66715359
ENSG00000069667.15	1980,78562	4263,811199	1,182669219	0,132950692	8,895547674	5,81E-19	3,95E-17	RORA	RAR-related orphan receptor A	6095	16,40328779	Upregulated	4834,567594
ENSG00000115363.13	79,36161903	18,2389403	-2,219312394	0,249536866	-8,893725513	5,91E-19	4,00E-17	EVA1A	eva-1 homolog A (C. elegans)	84141	16,39795303	Downregulated	2,958270614
ENSG00000197057.8	13,21078778	80,51938996	1,981596973	0,223335833	8,872722964	7,14E-19	4,79E-17	DTHD1	death domain containing 1	401124	16,31948408	Upregulated	97,34654051
ENSG00000227028.6	232,0942317	70,11417841	-2,14156121	0,241467517	-8,868941212	7,38E-19	4,94E-17	SLC8A1-AS1	SLC8A1 antisense RNA 1	100128590	16,30650299	Downregulated	29,61916508
ENSG00000139289.13	411,710139	856,0446718	1,137436917	0,128914425	8,823193487	1,11E-18	7,38E-17	PHLDA1	pleckstrin homology-like domain, family A, member 1	22822	16,13207411	Upregulated	967,1283051
ENSG00000116661.9	2,533223638	53,81821346	2,159833891	0,244847988	8,821121654	1,13E-18	7,49E-17	FBXO2	F-box protein 2	26232	16,1257842	Upregulated	66,63946091
ENSG00000073282.12	531,4925021	229,7637812	-1,520926876	0,173244425	-8,779081228	1,65E-18	1,08E-16	TP63	tumor protein p63	8626	15,96828947	Downregulated	154,331601
ENSG00000205730.6	51,42789029	163,5845969	1,550161865	0,17701908	8,757032663	2,00E-18	1,29E-16	ITPRIPL2	inositol 1,4,5-trisphosphate receptor interacting protein-like 2	162073	15,88972293	Upregulated	191,6237736
ENSG00000031081.10	36,42307331	184,917747	1,885653988	0,21533268	8,756933623	2,01E-18	1,29E-16	ARHGAP31	Rho GTPase activating protein 31	57514	15,88972293	Upregulated	222,0414155
ENSG00000244165.1	229,2013221	478,8900425	1,096544959	0,126048418	8,699394837	3,34E-18	2,11E-16	P2RY11	purinergic receptor P2Y, G-protein coupled, 11	5032	15,6755578	Upregulated	541,3122226
ENSG00000232434.2	126,7683556	282,5312456	1,218045408	0,140264465	8,683920116	3,82E-18	2,40E-16	C9orf172	chromosome 9 open reading frame 172	389813	15,61971872	Upregulated	321,4719681
ENSG00000157107.13	9,758526723	59,69995634	1,9461817	0,224361563	8,67430978	4,16E-18	2,60E-16	FCHO2	FCH domain only 2	115548	15,58468012	Upregulated	72,18531374
ENSG00000114739.13	638,9610719	364,72408	-1,041755193	0,120409006	-8,651804617	5,07E-18	3,16E-16	ACVR2B	activin A receptor, type IIB	93	15,50055674	Downregulated	296,1648321
ENSG00000239713.7	2329,667272	4431,846688	1,007411458	0,116675428	8,634306972	5,91E-18	3,67E-16	APOBEC3G	apolipoprotein B mRNA editing enzyme, catalytic polypeptide-like 3G	60489	15,43566186	Upregulated	4957,391542
ENSG00000136002.16	184,3615735	82,53793824	-1,473991627	0,171266518	-8,60642025	7,54E-18	4,64E-16	ARHGEF4	Rho guanine nucleotide exchange factor (GEF) 4	50649	15,33315616	Downregulated	57,08202942
ENSG00000198752.10	38,68396216	148,8257899	1,665312572	0,194109989	8,579221399	9,55E-18	5,84E-16	CDC42BPB	CDC42 binding protein kinase beta (DMPK-like)	9578	15,23355637	Upregulated	176,3612468
ENSG00000111537.4	143,9613921	434,7043122	1,484894502	0,173111579	8,577672935	9,68E-18	5,90E-16	IFNG	interferon, gamma	3458	15,22932254	Upregulated	507,3900422
ENSG00000138795.9	11892,43096	6752,571516	-1,124687188	0,131579124	-8,54761115	1,26E-17	7,57E-16	LEF1	lymphoid enhancer-binding factor 1	51176	15,1208447	Downregulated	5467,606654
ENSG00000006756.15	61,8720246	162,0090141	1,393861907	0,163429314	8,528836529	1,48E-17	8,87E-16	ARSD	arylsulfatase D	414	15,05188398	Upregulated	187,0432614
ENSG00000174600.13	4,162091738	69,12545147	2,063336409	0,242751253	8,499797158	1,90E-17	1,13E-15	CMKLR1	chemokine-like receptor 1	1240	14,94622009	Upregulated	85,3662914
ENSG00000186594.12	168,2380536	370,1823982	1,198933454	0,14115356	8,493823678	2,00E-17	1,18E-15	MIR22HG	MIR22 host gene (non-protein coding)	84981	14,92857497	Upregulated	420,6684843
ENSG00000232022.7	4,536459565	47,19214692	2,023449268	0,239675468	8,442454652	3,11E-17	1,81E-15	FAAHP1	NA	NA	14,74173606	Upregulated	57,85606876
ENSG00000099282.9	11,64504774	90,32676453	1,94734941	0,230969345	8,431202883	3,42E-17	1,99E-15	TSPAN15	tetraspanin 15	23555	14,70148218	Upregulated	109,9971937
ENSG00000152689.17	267,9043667	560,3732121	1,118286836	0,133274094	8,390879321	4,83E-17	2,78E-15	RASGRP3	RAS guanyl releasing protein 3 (calcium and DAG-regulated)	25780	14,55521695	Upregulated	633,4904235
ENSG00000073861.2	877,0312471	1945,347196	1,126691084	0,134391246	8,383664278	5,13E-17	2,94E-15	TBX21	T-box 21	30009	14,5316092	Upregulated	2212,426184
ENSG00000167633.17	12,39378339	80,69159201	1,917226765	0,230098808	8,33218904	7,94E-17	4,52E-15	KIR3DL1	killer cell immunoglobulin-like receptor, three domains, long cytoplasmic tail, 1	3811	14,3451771	Upregulated	97,76604417
ENSG00000101400.5	227,4557391	448,9195361	1,060464139	0,12733594	8,328081899	8,22E-17	4,66E-15	SNTA1	syntrophin, alpha 1	6640	14,33161036	Upregulated	504,2854853
ENSG00000185215.8	4,419744653	52,80477811	2,019059041	0,242503546	8,325894911	8,37E-17	4,73E-15	TNFAIP2	tumor necrosis factor, alpha-induced protein 2	7127	14,32508542	Upregulated	64,90103647
ENSG00000136193.16	105,2333843	229,4509849	1,180742588	0,142270259	8,299293182	1,05E-16	5,90E-15	SCRN1	secernin 1	9805	14,22918779	Upregulated	260,5053851
ENSG00000160856.20	22,34683497	99,15595184	1,756065072	0,212831562	8,250961741	1,57E-16	8,79E-15	FCRL3	Fc receptor-like 3	115352	14,05598935	Upregulated	118,3582311
ENSG00000119917.13	20,69374378	88,77813906	1,7570968	0,213189653	8,241942192	1,69E-16	9,42E-15	IFIT3	interferon-induced protein with tetratricopeptide repeats 3	3437	14,02616925	Upregulated	105,7992379
ENSG00000078900.14	25,38582323	100,0863949	1,664760528	0,202152059	8,23518955	1,79E-16	9,93E-15	TP73	tumor protein p73	7161	14,00312711	Upregulated	118,7615378
ENSG00000140443.13	625,6152269	313,8114277	-1,26179171	0,153521974	-8,218964843	2,05E-16	1,13E-14	IGF1R	insulin-like growth factor 1 receptor	3480	13,9457836	Downregulated	235,860478
ENSG00000112561.17	189,8559755	357,6509931	1,007399563	0,122672721	8,212091091	2,17E-16	1,19E-14	TFEB	transcription factor EB	7942	13,92525526	Upregulated	399,5997475
ENSG00000215246.5	138,9235685	57,05863574	-1,595226206	0,195589436	-8,155993696	3,46E-16	1,88E-14	RP11-43F13.3	NA	100506688	13,72585132	Downregulated	36,59240256
ENSG00000225720.6	101,6937721	266,3401551	1,358465499	0,166575581	8,155249931	3,48E-16	1,89E-14	RP4-742C19.12	NA	NA	13,7246094	Upregulated	307,5017508
ENSG00000136960.12	318,7883275	118,6371244	-1,737146443	0,21315959	-8,149511102	3,65E-16	1,97E-14	ENPP2	ectonucleotide pyrophosphatase/phosphodiesterase 2	5168	13,70541999	Downregulated	68,59932367
ENSG00000171316.11	1413,647444	770,693058	-1,176021756	0,144593498	-8,133296239	4,18E-16	2,24E-14	CHD7	chromodomain helicase DNA binding protein 7	55636	13,65008592	Downregulated	609,9544615
ENSG00000145284.11	38,44099112	160,3920705	1,649411791	0,204135482	8,079985792	6,48E-16	3,45E-14	SCD5	stearoyl-CoA desaturase 5	79966	13,46244252	Upregulated	190,8798404
ENSG00000154127.9	2023,545803	1174,486198	-1,08311475	0,13419315	-8,071311749	6,95E-16	3,69E-14	UBASH3B	ubiquitin associated and SH3 domain containing B	84959	13,43297089	Downregulated	962,2212964
ENSG00000205809.9	19,06487568	104,0395175	1,793323147	0,22269506	8,052819617	8,09E-16	4,27E-14	KLRC2	killer cell lectin-like receptor subfamily C, member 2	3822	13,37007074	Upregulated	125,2831779
ENSG00000167912.5	28,33231966	107,4029221	1,656177093	0,205674095	8,052434087	8,12E-16	4,27E-14	RP11-25K19.1	NA	NA	13,37007074	Upregulated	127,1705727
ENSG00000052126.14	121,847987	307,1271027	1,244449979	0,154565111	8,051299368	8,19E-16	4,29E-14	PLEKHA5	pleckstrin homology domain containing, family A member 5	54477	13,36742862	Upregulated	353,4468816
ENSG00000273184.1	0,982165478	48,91974663	2,006141021	0,250212806	8,017739188	1,08E-15	5,62E-14	RP11-212P7.3	NA	NA	13,24994464	Upregulated	60,90414192
ENSG00000142156.14	30,33555559	323,2017298	1,934621887	0,241435447	8,012998569	1,12E-15	5,83E-14	COL6A1	collagen, type VI, alpha 1	1291	13,23456951	Upregulated	396,4182734
ENSG00000113269.13	55,79918876	145,8850422	1,362531578	0,170582415	7,987526627	1,38E-15	7,05E-14	RNF130	ring finger protein 130	55819	13,15154339	Upregulated	168,4065056
ENSG00000206561.12	86,98551303	192,8729192	1,191248382	0,149638313	7,960851435	1,71E-15	8,70E-14	COLQ	collagen-like tail subunit (single strand of homotrimer) of asymmetric acetylcholinesterase	8292	13,06045045	Upregulated	219,3447707
ENSG00000186350.9	36,51556513	120,8707702	1,600309277	0,201157967	7,955485442	1,78E-15	9,06E-14	RXRA	retinoid X receptor, alpha	6256	13,04296282	Upregulated	141,9595715
ENSG00000116396.13	252,6228144	121,1167856	-1,324773751	0,166659524	-7,948983177	1,88E-15	9,49E-14	KCNC4	potassium voltage-gated channel, Shaw-related subfamily, member 4	3749	13,02283443	Downregulated	88,24027844
ENSG00000163827.12	80,96112337	24,31024294	-1,942620101	0,245385486	-7,916605546	2,44E-15	1,22E-13	LRRC2	leucine rich repeat containing 2	79442	12,91486412	Downregulated	10,14752283
ENSG00000064652.10	47,35829037	129,8038337	1,415544819	0,180457403	7,844204752	4,36E-15	2,13E-13	SNX24	sorting nexin 24	28966	12,67232356	Upregulated	150,4152195
ENSG00000169258.6	29,88337782	100,1568696	1,569121874	0,200169668	7,838959262	4,54E-15	2,21E-13	GPRIN1	G protein regulated inducer of neurite outgrowth 1	114787	12,65546479	Upregulated	117,7252426
ENSG00000109956.12	5,970802812	222,3739552	1,957070916	0,250270104	7,819834982	5,29E-15	2,55E-13	B3GAT1	beta-1,3-glucuronyltransferase 1 (glucuronosyltransferase P)	27087	12,59324614	Upregulated	276,4747432
ENSG00000114013.15	107,6014469	293,3596703	1,328040371	0,170192311	7,803174894	6,04E-15	2,89E-13	CD86	CD86 molecule	942	12,53836852	Upregulated	339,7992262
ENSG00000162692.10	10,85740711	69,60505643	1,826621132	0,235299856	7,762950483	8,30E-15	3,94E-13	VCAM1	vascular cell adhesion molecule 1	7412	12,40401043	Upregulated	84,29196876
ENSG00000116690.12	248,8894175	71,31523297	-1,918063185	0,248382182	-7,722225363	1,14E-14	5,40E-13	PRG4	proteoglycan 4	10216	12,26735897	Downregulated	26,92168684
ENSG00000066468.20	5,183162187	55,54953427	1,872728954	0,242674792	7,717031253	1,19E-14	5,61E-13	FGFR2	fibroblast growth factor receptor 2	2263	12,25090828	Upregulated	68,14112729
ENSG00000228956.8	576,1993736	331,5584934	-1,021619283	0,13247855	-7,711582617	1,24E-14	5,84E-13	SATB1-AS1	NA	NA	12,23393342	Downregulated	270,3982734
ENSG00000179630.10	39,78284255	106,111956	1,371717552	0,178929916	7,666228105	1,77E-14	8,21E-13	LACC1	laccase (multicopper oxidoreductase) domain containing 1	144811	12,08582788	Upregulated	122,6942344
ENSG00000118156.12	16,49274707	65,17051343	1,638704226	0,214115428	7,653368271	1,96E-14	9,02E-13	ZNF541	zinc finger protein 541	84215	12,04478195	Upregulated	77,33995502
ENSG00000155966.13	183,2480113	81,75327952	-1,436178032	0,189736718	-7,569320523	3,75E-14	1,69E-12	AFF2	AF4/FMR2 family, member 2	2334	11,77193846	Downregulated	56,37959658
ENSG00000142192.20	1876,358999	1003,038357	-1,186366869	0,157164653	-7,548560371	4,40E-14	1,97E-12	APP	amyloid beta (A4) precursor protein	351	11,70500584	Downregulated	784,7081972
ENSG00000008256.15	32,55753946	92,13648327	1,41431202	0,187407503	7,546720382	4,46E-14	2,00E-12	CYTH3	cytohesin 3	9265	11,70005385	Upregulated	107,0312192
ENSG00000227191.7	780,8002928	1432,680979	1,02029424	0,13531297	7,540254542	4,69E-14	2,09E-12	TRGC2	T cell receptor gamma constant 2	NA	11,6796895	Upregulated	1595,651151
ENSG00000163818.16	82,26921049	202,950119	1,302744337	0,172790375	7,539449658	4,72E-14	2,10E-12	LZTFL1	leucine zipper transcription factor-like 1	54585	11,6781844	Upregulated	233,1203461
ENSG00000255441.1	2,042140898	36,51298283	1,872069802	0,248652183	7,528869372	5,12E-14	2,26E-12	CTD-2616J11.2	NA	NA	11,64531638	Upregulated	45,13069332
ENSG00000134321.11	56,79089545	156,1537384	1,382597145	0,184622089	7,488795917	6,95E-14	3,06E-12	RSAD2	radical S-adenosyl methionine domain containing 2	91543	11,5147217	Upregulated	180,9944492
ENSG00000041353.9	28,86230737	94,33065149	1,499335304	0,200736696	7,469163998	8,07E-14	3,53E-12	RAB27B	RAB27B, member RAS oncogene family	5874	11,45216944	Upregulated	110,6977375
ENSG00000176595.3	213,0726616	95,7523997	-1,47175062	0,197129343	-7,465913475	8,27E-14	3,61E-12	KBTBD11	kelch repeat and BTB (POZ) domain containing 11	9920	11,44259833	Downregulated	66,42233423
ENSG00000054793.13	33,85094471	126,645768	1,5674672	0,210229967	7,455964626	8,92E-14	3,87E-12	ATP9A	ATPase, class II, type 9A	10079	11,41209952	Upregulated	149,8444738
ENSG00000164283.12	9,680716782	60,11668147	1,741404588	0,233987442	7,442299342	9,89E-14	4,27E-12	ESM1	endothelial cell-specific molecule 1	11082	11,36940622	Upregulated	72,72567264
ENSG00000198910.12	27,21875739	109,9586181	1,584639109	0,212960477	7,441000938	9,99E-14	4,30E-12	L1CAM	L1 cell adhesion molecule	3897	11,36627508	Upregulated	130,6435833
ENSG00000130396.20	523,7856575	1017,92159	1,040842465	0,139960563	7,436683898	1,03E-13	4,42E-12	MLLT4	myeloid/lymphoid or mixed-lineage leukemia (trithorax homolog, Drosophila); translocated to, 4	4301	11,35435244	Upregulated	1141,455573
ENSG00000169413.2	212,9515461	93,97193146	-1,483565367	0,199935381	-7,420224282	1,17E-13	5,00E-12	RNASE6	ribonuclease, RNase A family, k6	6039	11,30145077	Downregulated	64,2270278
ENSG00000040608.13	22,81369462	75,00868152	1,517023427	0,206347312	7,35179639	1,96E-13	8,18E-12	RTN4R	reticulon 4 receptor	65078	11,0869982	Upregulated	88,05742875
ENSG00000120278.14	17,92709032	92,75414359	1,650210639	0,224832434	7,339735681	2,14E-13	8,89E-12	PLEKHG1	pleckstrin homology domain containing, family G (with RhoGef domain) member 1	57480	11,05113567	Upregulated	111,4609069
ENSG00000172348.14	81,90952455	260,7468122	1,384384954	0,189014225	7,324236857	2,40E-13	9,88E-12	RCAN2	regulator of calcineurin 2	10231	11,00526179	Upregulated	305,4561341
ENSG00000122877.13	30,35977868	95,82492127	1,458056587	0,199613431	7,304401199	2,79E-13	1,13E-11	EGR2	early growth response 2	1959	10,94543648	Upregulated	112,1912069
ENSG00000248334.6	31,09897312	104,598124	1,509762398	0,207206633	7,286264799	3,19E-13	1,28E-11	WHAMMP2	WAS protein homolog associated with actin, golgi membranes and microtubules pseudogene 2	NA	10,89121085	Upregulated	122,9729117
ENSG00000125355.15	3,476484145	46,13746724	1,797549503	0,246861491	7,281611619	3,30E-13	1,33E-11	TMEM255A	transmembrane protein 255A	55026	10,8772864	Upregulated	56,80271302
ENSG00000257924.1	48,99669968	12,25268902	-1,796721238	0,247235119	-7,267257368	3,67E-13	1,47E-11	RP11-493L12.5	NA	NA	10,83217105	Downregulated	3,066686361
ENSG00000117281.15	39,36956975	128,8867348	1,431604325	0,197193754	7,259886777	3,87E-13	1,55E-11	CD160	CD160 molecule	11126	10,81060828	Upregulated	151,2660261
ENSG00000103888.15	106,9547442	39,24422986	-1,650490723	0,228460172	-7,224413377	5,03E-13	2,00E-11	CEMIP	cell migration inducing protein, hyaluronan binding	57214	10,69908661	Downregulated	22,31660127
ENSG00000143167.11	642,8178047	305,889218	-1,395300427	0,193537632	-7,209452806	5,62E-13	2,22E-11	GPA33	glycoprotein A33 (transmembrane)	10223	10,65446315	Downregulated	221,6570714
ENSG00000167768.4	44,36774829	133,8407132	1,393281235	0,193525489	7,199471463	6,04E-13	2,37E-11	KRT1	keratin 1	3848	10,62473045	Upregulated	156,2089544
ENSG00000125384.6	672,2460442	2135,880688	1,492542135	0,20827785	7,166110735	7,72E-13	2,99E-11	PTGER2	prostaglandin E receptor 2 (subtype EP2), 53kDa	5732	10,52489279	Upregulated	2501,789349
ENSG00000112149.9	85,73101276	214,1944289	1,238113474	0,173039945	7,155073202	8,36E-13	3,23E-11	CD83	CD83 molecule	9308	10,49094332	Upregulated	246,3102829
ENSG00000111879.18	182,9998996	90,98894515	-1,235552334	0,172895961	-7,146218626	8,92E-13	3,43E-11	FAM184A	family with sequence similarity 184, member A	79632	10,46496117	Downregulated	67,98620655
ENSG00000196569.11	11,50410974	58,34602542	1,661892385	0,234016521	7,101602832	1,23E-12	4,66E-11	LAMA2	laminin, alpha 2	3908	10,33134898	Upregulated	70,05650434
ENSG00000134030.13	20,30469407	67,37018406	1,481718201	0,209080514	7,086830691	1,37E-12	5,15E-11	CTIF	CBP80/20-dependent translation initiation factor	9811	10,28794966	Upregulated	79,13655656
ENSG00000185261.13	67,01628182	145,3542265	1,128339775	0,159425439	7,077539078	1,47E-12	5,47E-11	KIAA0825	NA	285600	10,26178638	Upregulated	164,9387127
ENSG00000267519.3	110,5332614	216,1053734	1,02752118	0,145537442	7,060184411	1,66E-12	6,16E-11	CTD-3252C9.4	NA	284454	10,2104252	Upregulated	242,4984014
ENSG00000214944.9	1,021070449	32,78093502	1,770562648	0,250890368	7,057116856	1,70E-12	6,28E-11	ARHGEF28	Rho guanine nucleotide exchange factor (GEF) 28	64283	10,20181673	Upregulated	40,72090116
ENSG00000117643.14	179,5770023	443,8553076	1,190003977	0,169155579	7,034967345	1,99E-12	7,33E-11	MAN1C1	mannosidase, alpha, class 1C, member 1	57134	10,1346704	Upregulated	509,9248839
ENSG00000149557.12	4,123186767	36,70582861	1,725796282	0,245813044	7,0207677	2,21E-12	8,07E-11	FEZ1	fasciculation and elongation protein zeta 1 (zygin I)	9638	10,09339065	Upregulated	44,85148907
ENSG00000138119.16	244,6832801	109,4170816	-1,406739588	0,200772606	-7,006631126	2,44E-12	8,84E-11	MYOF	myoferlin	26509	10,05333091	Downregulated	75,60053195
ENSG00000105371.8	211,6728227	76,85648897	-1,687028837	0,241135106	-6,996197542	2,63E-12	9,51E-11	ICAM4	intercellular adhesion molecule 4 (Landsteiner-Wiener blood group)	3386	10,02193818	Downregulated	43,15240553
ENSG00000233093.5	85,2546119	192,5719278	1,179417032	0,169072311	6,975814243	3,04E-12	1,09E-10	LINC00892	long intergenic non-protein coding RNA 892	100128420	9,963622142	Upregulated	219,4012567
ENSG00000163701.18	9,267443984	52,02508679	1,651261211	0,236825094	6,972492595	3,11E-12	1,11E-10	IL17RE	interleukin 17 receptor E	132014	9,954305407	Upregulated	62,71449749
ENSG00000237541.3	157,7073083	403,2915005	1,212467146	0,174590492	6,944634445	3,79E-12	1,35E-10	HLA-DQA2	major histocompatibility complex, class II, DQ alpha 2	3117;3118	9,87125998	Upregulated	464,6875485
ENSG00000185189.15	107,7475255	220,9027408	1,111245052	0,16093279	6,905025718	5,02E-12	1,75E-10	NRBP2	nuclear receptor binding protein 2	340371	9,757174209	Upregulated	249,1915446
ENSG00000100453.12	611,2622532	2953,530342	1,542123168	0,223532686	6,898871003	5,24E-12	1,82E-10	GZMB	granzyme B (granzyme 2, cytotoxic T-lymphocyte-associated serine esterase 1)	3002	9,739272227	Upregulated	3539,097364
ENSG00000245954.6	651,2080365	379,0396432	-1,038135591	0,150658313	-6,890662525	5,55E-12	1,92E-10	RP11-18H21.1	NA	100996286	9,716941627	Downregulated	310,9975448
ENSG00000051128.18	5,027542305	37,36332807	1,679238077	0,243866736	6,885884089	5,74E-12	1,98E-10	HOMER3	homer homolog 3 (Drosophila)	9454	9,70326844	Upregulated	45,44727452
ENSG00000147459.17	44,00806235	130,952716	1,382038911	0,200938581	6,877917141	6,07E-12	2,09E-10	DOCK5	dedicator of cytokinesis 5	80005	9,679883084	Upregulated	152,6888794
ENSG00000176485.10	34,84779206	116,3350269	1,460697352	0,213534601	6,840565151	7,89E-12	2,69E-10	PLA2G16	phospholipase A2, group XVI	11145	9,569959538	Upregulated	136,7068356
ENSG00000104361.9	17,21725963	61,64832207	1,511115029	0,220940718	6,839459215	7,95E-12	2,71E-10	NIPAL2	NIPA-like domain containing 2	79815	9,567506864	Upregulated	72,75608767
ENSG00000136856.17	104,9515083	204,4653789	1,007704708	0,14765822	6,824575731	8,82E-12	2,99E-10	SLC2A8	solute carrier family 2 (facilitated glucose transporter), member 8	29988	9,524231239	Upregulated	229,3438465
ENSG00000177191.2	53,54784113	120,9037313	1,171225844	0,1716685	6,822601941	8,94E-12	3,03E-10	B3GNT8	UDP-GlcNAc:betaGal beta-1,3-N-acetylglucosaminyltransferase 8	374907	9,519155817	Upregulated	137,7427039
ENSG00000189350.12	287,7913875	630,8120689	1,034690862	0,151732738	6,819166869	9,16E-12	3,09E-10	FAM179A	family with sequence similarity 179, member A	165186	9,509662701	Upregulated	716,5672393
ENSG00000021762.19	73,05021269	422,0148286	1,597574243	0,234625617	6,809035881	9,83E-12	3,30E-10	OSBPL5	oxysterol binding protein-like 5	114879	9,480841217	Upregulated	509,2559826
ENSG00000243244.5	35,32419292	106,3661869	1,400603574	0,206017257	6,798476938	1,06E-11	3,54E-10	STON1	stonin 1	11037	9,450766329	Upregulated	124,1266854
ENSG00000244693.1	6,889840229	65,55512037	1,68651473	0,248746194	6,780062447	1,20E-11	3,98E-10	CTAGE8	CTAGE family, member 8	100142659	9,400438474	Upregulated	80,2214404
ENSG00000163359.15	538,0035739	212,3733749	-1,5196367	0,224441822	-6,770737669	1,28E-11	4,21E-10	COL6A3	collagen, type VI, alpha 3	1293	9,37521485	Downregulated	130,9658252
ENSG00000184731.5	115,4734526	48,23631927	-1,450171774	0,214843461	-6,749899513	1,48E-11	4,82E-10	FAM110C	family with sequence similarity 110, member C	642273	9,317070398	Downregulated	31,42703594
ENSG00000226979.8	178,8818535	379,8806361	1,051875778	0,156038951	6,741110272	1,57E-11	5,11E-10	LTA	lymphotoxin alpha	4049	9,291850153	Upregulated	430,1303318
ENSG00000071246.10	30,82663833	104,7247354	1,450890115	0,215237031	6,74089449	1,57E-11	5,11E-10	VASH1	vasohibin 1	22846	9,291850153	Upregulated	123,1992597
ENSG00000143153.12	17,35819764	63,61804694	1,490036055	0,221596903	6,724083385	1,77E-11	5,67E-10	ATP1B1	ATPase, Na+/K+ transporting, beta 1 polypeptide	481	9,246764706	Upregulated	75,18300927
ENSG00000157985.18	17,10054472	67,54031767	1,530320025	0,227696536	6,720875298	1,81E-11	5,76E-10	AGAP1	ArfGAP with GTPase domain, ankyrin repeat and PH domain 1	116987	9,239731961	Upregulated	80,1502609
ENSG00000167094.15	97,33715553	365,2348315	1,386712589	0,206569516	6,713055309	1,91E-11	6,05E-10	TTC16	tetratricopeptide repeat domain 16	158248	9,218114663	Upregulated	432,2092505
ENSG00000166681.13	461,4651155	243,0572436	-1,230551823	0,183330798	-6,712193688	1,92E-11	6,08E-10	NGFRAP1	nerve growth factor receptor (TNFRSF16) associated protein 1	27018	9,216386909	Downregulated	188,4552756
ENSG00000256039.1	2713,772524	1508,073365	-1,08968553	0,162416946	-6,70918619	1,96E-11	6,19E-10	RP11-291B21.2	NA	NA	9,208270976	Downregulated	1206,648575
ENSG00000128656.13	17,12476781	61,78955101	1,464079078	0,218349355	6,705213668	2,01E-11	6,35E-10	CHN1	chimerin 1	1123	9,197286911	Upregulated	72,9557468
ENSG00000174807.3	424,5803232	234,0129872	-1,059167484	0,158361053	-6,68830791	2,26E-11	7,09E-10	CD248	CD248 molecule, endosialin	57124	9,149560166	Downregulated	186,3711532
ENSG00000106089.11	64,92055407	133,0169499	1,066689897	0,159709411	6,678942007	2,41E-11	7,53E-10	STX1A	syntaxin 1A (brain)	6804	9,123444607	Upregulated	150,0410489
ENSG00000134326.11	89,32421182	175,7120981	1,023675388	0,153368741	6,674602542	2,48E-11	7,74E-10	CMPK2	cytidine monophosphate (UMP-CMP) kinase 2, mitochondrial	129607	9,111415571	Upregulated	197,3090697
ENSG00000223509.8	73,55597731	166,060258	1,151489797	0,172567201	6,672703679	2,51E-11	7,82E-10	RP11-632K20.7	NA	NA	9,106616787	Upregulated	189,1863282
ENSG00000138311.15	8,776361245	46,12551946	1,589421721	0,238241883	6,671462216	2,53E-11	7,87E-10	ZNF365	zinc finger protein 365	22891	9,103763684	Upregulated	55,46280902
ENSG00000147889.16	83,32918591	275,2706201	1,421179415	0,214172053	6,635690298	3,23E-11	9,93E-10	CDKN2A	cyclin-dependent kinase inhibitor 2A	1029	9,003089429	Upregulated	323,2559786
ENSG00000169047.5	281,6113779	149,0851017	-1,134938786	0,171037759	-6,635603706	3,23E-11	9,93E-10	IRS1	insulin receptor substrate 1	3667	9,003089429	Downregulated	115,9535326
ENSG00000277443.1	1,512153188	27,40784563	1,655915122	0,2510039	6,597168885	4,19E-11	1,27E-09	MARCKS	myristoylated alanine-rich protein kinase C substrate	4082	8,896386391	Upregulated	33,88176875
ENSG00000128683.13	105,3552399	27,19419058	-1,596260775	0,242124155	-6,592736593	4,32E-11	1,30E-09	GAD1	glutamate decarboxylase 1 (brain, 67kDa)	2571	8,885804665	Downregulated	7,653928243
ENSG00000276231.4	176,6840927	68,47856562	-1,518287459	0,230418731	-6,589253632	4,42E-11	1,33E-09	PIK3R6	phosphoinositide-3-kinase, regulatory subunit 6	146850	8,876409627	Downregulated	41,42718386
ENSG00000169554.16	360,8364595	821,906446	1,071378266	0,16303212	6,571577829	4,98E-11	1,49E-09	ZEB2	zinc finger E-box binding homeobox 2	9839	8,827941282	Upregulated	937,1739426

ENSG00000125657.4	30,56898541	98,13481288	1,409943075	0,214776606	6,564695772	5,21E-11	1,55E-09	TNFSF9	tumor necrosis factor (ligand) superfamily, member 9	8744	8,810745132	Upregulated	115,0262697
ENSG00000205683.11	12,62721322	52,63781113	1,517772727	0,231205562	6,564603	5,22E-11	1,55E-09	DPF3	D4, zinc and double PHD fingers, family 3	8110	8,810745132	Upregulated	62,64046061
ENSG00000272763.1	1,05997542	27,67381195	1,64827677	0,251776635	6,546583514	5,89E-11	1,74E-09	RP11-357H14.17	NA	NA	8,760640082	Upregulated	34,32727109
ENSG00000184792.15	203,135032	111,7820739	-1,034854485	0,158981868	-6,509261088	7,55E-11	2,21E-09	OSBP2	oxysterol binding protein 2	23762	8,65632548	Downregulated	88,94383437
ENSG00000137628.16	473,4111217	951,193349	1,01523942	0,156004436	6,507759956	7,63E-11	2,22E-09	DDX60	DEAD (Asp-Glu-Ala-Asp) box polypeptide 60	55601	8,652757158	Upregulated	1070,638906
ENSG00000048052.21	4,692079448	45,6785557	1,619024594	0,24947637	6,489691176	8,60E-11	2,48E-09	HDAC9	histone deacetylase 9	9734	8,605200187	Upregulated	55,92517476
ENSG00000196407.11	55,29342414	128,4706981	1,18238223	0,182312197	6,485480671	8,84E-11	2,54E-09	THEM5	thioesterase superfamily member 5	284486	8,594588339	Upregulated	146,7650166
ENSG00000158292.6	2,611033579	29,91214873	1,607140009	0,250397435	6,418356511	1,38E-10	3,87E-09	GPR153	G protein-coupled receptor 153	387509	8,412682321	Upregulated	36,73742752
ENSG00000010278.11	327,3650233	168,1143729	-1,208963839	0,189038574	-6,395328802	1,60E-10	4,46E-09	CD9	CD9 molecule	928	8,350805786	Downregulated	128,3017104
ENSG00000034053.14	84,4522894	182,6457828	1,093601029	0,171008867	6,394996032	1,61E-10	4,46E-09	APBA2	amyloid beta (A4) precursor protein-binding, family A, member 2	321	8,350594173	Upregulated	207,1941561
ENSG00000104312.7	50,03245201	124,9193182	1,200450837	0,188129248	6,380989941	1,76E-10	4,86E-09	RIPK2	receptor-interacting serine-threonine kinase 2	8767	8,313022333	Upregulated	143,6410347
ENSG00000171954.12	18,87035082	66,89970432	1,406106246	0,220578137	6,374640137	1,83E-10	5,04E-09	CYP4F22	cytochrome P450, family 4, subfamily F, polypeptide 22	126410	8,297849072	Upregulated	78,90704269
ENSG00000076356.6	123,5054787	51,21763525	-1,421220582	0,22349591	-6,359045141	2,03E-10	5,56E-09	PLXNA2	plexin A2	5362	8,255240206	Downregulated	33,14567439
ENSG00000150722.10	24,0777361	5,320981706	-1,342883284	0,212446391	-6,321045409	2,60E-10	7,09E-09	PPP1R1C	protein phosphatase 1, regulatory (inhibitor) subunit 1C	151242	8,149565634	Downregulated	0,631793107
ENSG00000007237.18	21,8946572	71,02702327	1,382164505	0,218863716	6,315183384	2,70E-10	7,34E-09	GAS7	growth arrest-specific 7	8522	8,134533854	Upregulated	83,31011479
ENSG00000152192.7	109,2839018	39,25092945	-1,539620019	0,243991513	-6,310137591	2,79E-10	7,55E-09	POU4F1	POU class 4 homeobox 1	5457	8,121802288	Downregulated	21,74268636
ENSG00000173040.12	18,84612773	60,35514691	1,396785562	0,221542095	6,304831415	2,89E-10	7,80E-09	EVC2	Ellis van Creveld syndrome 2	132884	8,107632993	Upregulated	70,7324017
ENSG00000171101.13	136,1084688	580,3736705	1,462433724	0,232559325	6,288432964	3,21E-10	8,59E-09	SIGLEC17P	sialic acid binding Ig-like lectin 17, pseudogene	284367	8,065974611	Upregulated	691,4399709
ENSG00000064989.12	38,23178439	11,29230196	-1,578562097	0,251880283	-6,267112605	3,68E-10	9,82E-09	CALCRL	calcitonin receptor-like	10203	8,007850942	Downregulated	4,55743135
ENSG00000184979.9	88,41985628	188,4977963	1,080308522	0,172549273	6,260869748	3,83E-10	1,02E-08	USP18	ubiquitin specific peptidase 18	11274	7,992564558	Upregulated	213,5172814
ENSG00000145990.10	42,45700419	108,3763018	1,241631803	0,198492225	6,255317059	3,97E-10	1,05E-08	GFOD1	glucose-fructose oxidoreductase domain containing 1	54438	7,977806401	Upregulated	124,8561261
ENSG00000165409.15	217,7214355	95,99720272	-1,379381648	0,221099342	-6,238741523	4,41E-10	1,16E-08	TSHR	thyroid stimulating hormone receptor	7253	7,934529356	Downregulated	65,56614453
ENSG00000131459.12	321,7964718	155,0065476	-1,1837249	0,189838661	-6,235425894	4,51E-10	1,18E-08	GFPT2	glutamine-fructose-6-phosphate transaminase 2	9945	7,92671766	Downregulated	113,3090665
ENSG00000166532.15	3,593199057	27,49848966	1,548003473	0,248340401	6,233393626	4,56E-10	1,20E-08	RIMKLB	ribosomal modification protein rimK-like family member B	57494	7,921773283	Upregulated	33,47481231
ENSG00000166068.12	2,11995084	26,93538811	1,564564482	0,251266802	6,22670591	4,76E-10	1,25E-08	SPRED1	sprouty-related, EVH1 domain containing 1	161742	7,9046168	Upregulated	33,13924742
ENSG00000172031.6	8,698551303	39,30559253	1,479227136	0,237609355	6,225458308	4,80E-10	1,25E-08	EPHX4	epoxide hydrolase 4	253152	7,901850221	Upregulated	46,95735284
ENSG00000104951.15	4,988637334	39,13127831	1,546088855	0,248404801	6,224069928	4,84E-10	1,26E-08	IL4I1	interleukin 4 induced 1	259307	7,8986933	Upregulated	47,66693856
ENSG00000125498.19	7,599670913	40,07356355	1,50742687	0,242716965	6,210636627	5,28E-10	1,36E-08	KIR2DL1	killer cell immunoglobulin-like receptor, two domains, long cytoplasmic tail, 1	3802	7,864953862	Upregulated	48,19203671
ENSG00000188305.5	14,51373423	53,35140794	1,416900204	0,228189025	6,209326686	5,32E-10	1,37E-08	C19orf35	chromosome 19 open reading frame 35	374872	7,86201594	Upregulated	63,06082637
ENSG00000164136.16	31,53646901	77,87262188	1,209486277	0,194883259	6,206209215	5,43E-10	1,40E-08	IL15	interleukin 15	3600	7,854084466	Upregulated	89,4566601
ENSG00000100154.14	69,17513763	22,34175839	-1,557644974	0,251929396	-6,182863142	6,29E-10	1,61E-08	TTC28	tetratricopeptide repeat domain 28	23331	7,793781963	Downregulated	10,63341357
ENSG00000203710.10	532,424001	280,1821048	-1,172398688	0,189852752	-6,1753052	6,60E-10	1,68E-08	CR1	complement component (3b/4b) receptor 1 (Knops blood group)	1378	7,774340101	Downregulated	217,1216307
ENSG00000277089.4	3,515389116	30,6699522	1,537485767	0,249227259	6,169011251	6,87E-10	1,74E-08	AC069363.1	NA	NA	7,759058945	Upregulated	37,45859297
ENSG00000196460.12	68,76186483	24,85643964	-1,508508334	0,24495579	-6,15828812	7,35E-10	1,86E-08	RFX8	RFX family member 8, lacking RFX DNA binding domain	731220	7,731637554	Downregulated	13,88008334
ENSG00000164512.17	337,0993269	172,6081437	-1,17232235	0,190636218	-6,149525856	7,77E-10	1,95E-08	ANKRD55	ankyrin repeat domain 55	79722	7,708960262	Downregulated	131,4853479
ENSG00000176658.16	68,34859203	152,6772922	1,133217487	0,184778286	6,132849866	8,63E-10	2,16E-08	MYO1D	myosin ID	4642	7,665347876	Upregulated	173,7594672
ENSG00000121297.6	17,96599529	61,77249708	1,383440632	0,226250065	6,114652984	9,68E-10	2,40E-08	TSHZ3	teashirt zinc finger homeobox 3	57616	7,619016267	Upregulated	72,72412252
ENSG00000175779.1	31,84770878	78,62169513	1,207168032	0,198210539	6,090332228	1,13E-09	2,80E-08	C15orf53	chromosome 15 open reading frame 53	400359	7,553566657	Upregulated	90,31519172
ENSG00000157833.12	13,68718864	46,59404446	1,374259904	0,226206308	6,075250134	1,24E-09	3,05E-08	GAREML	GRB2 associated, regulator of MAPK1-like	150946	7,515311033	Upregulated	54,82075842
ENSG00000226423.1	139,162139	73,58666095	-1,101538739	0,181345564	-6,074252451	1,25E-09	3,07E-08	AC093642.4	NA	NA	7,513261645	Downregulated	57,19279145
ENSG00000147650.11	44,88819479	102,8113173	1,1429992	0,18833786	6,068876428	1,29E-09	3,15E-08	LRP12	low density lipoprotein receptor-related protein 12	29967	7,50107267	Upregulated	117,2920979
ENSG00000219481.10	42,33074806	103,0529035	1,181868977	0,195353181	6,049909044	1,45E-09	3,53E-08	NBPF1	neuroblastoma breakpoint family, member 1	NA	7,452682393	Upregulated	118,2334424
ENSG00000250303.3	33,26737015	82,40965467	1,200500155	0,198623514	6,044098863	1,50E-09	3,65E-08	RP11-356J5.12	NA	283140	7,437668703	Upregulated	94,6952258
ENSG00000108797.11	144,5545079	421,508249	1,218527556	0,201682206	6,041819855	1,52E-09	3,70E-08	CNTNAP1	contactin associated protein 1	8506	7,432172801	Upregulated	490,7466843
ENSG00000113758.13	483,8714181	251,302274	-1,124023893	0,186357374	-6,031550407	1,62E-09	3,92E-08	DBN1	drebrin 1	1627	7,407101156	Downregulated	193,159988
ENSG00000186648.14	11,27067991	42,82488189	1,404849469	0,233492535	6,016678311	1,78E-09	4,28E-08	LRRC16B	leucine rich repeat containing 16B	90668	7,36844402	Upregulated	50,71343238
ENSG00000274767.1	7,716385825	45,22523186	1,465026034	0,244050617	6,002959764	1,94E-09	4,64E-08	AC131056.3	NA	NA	7,333595999	Upregulated	54,60244337
ENSG00000072840.12	32,72784122	85,75946973	1,253017699	0,209046036	5,993979701	2,05E-09	4,89E-08	EVC	Ellis van Creveld syndrome	2121	7,310846276	Upregulated	99,01737685
ENSG00000105492.15	13,49266378	50,32611246	1,383652153	0,230862171	5,993412208	2,05E-09	4,90E-08	SIGLEC6	sialic acid binding Ig-like lectin 6	946	7,309959846	Upregulated	59,53447463
ENSG00000187037.8	0,491082739	33,33770495	1,500256951	0,252160596	5,949608994	2,69E-09	6,33E-08	GPR141	G protein-coupled receptor 141	353345	7,198339223	Upregulated	41,54936051
ENSG00000185340.15	29,96118776	92,7679024	1,305973434	0,219756449	5,942821888	2,80E-09	6,57E-08	GAS2L1	growth arrest-specific 2 like 1	10634	7,182351008	Upregulated	108,4695811
ENSG00000156381.8	62,75215705	128,8717156	1,046506778	0,17651104	5,928846039	3,05E-09	7,13E-08	ANKRD9	ankyrin repeat domain 9	122416	7,147058701	Upregulated	145,4016052
ENSG00000204577.11	50,75696457	120,4125609	1,138332581	0,192474153	5,914210106	3,33E-09	7,74E-08	LILRB3	leukocyte immunoglobulin-like receptor, subfamily B (with TM and ITIM domains), member 3	102725035;10288;11025	7,111473774	Upregulated	137,82646
ENSG00000105974.11	4,988637334	34,17977745	1,468929837	0,248565834	5,909620868	3,43E-09	7,92E-08	CAV1	caveolin 1, caveolae protein, 22kDa	857	7,101204471	Upregulated	41,47756248
ENSG00000255571.6	25,30801328	69,64426872	1,240074984	0,209968622	5,906001434	3,51E-09	8,07E-08	MIR9-3HG	NA	254559	7,092883559	Upregulated	80,72833258
ENSG00000134215.15	60,81204918	153,0820697	1,128546611	0,191471598	5,894067963	3,77E-09	8,63E-08	VAV3	vav 3 guanine nucleotide exchange factor	10451	7,063898063	Upregulated	176,1495749
ENSG00000198574.5	17,34351576	58,89683689	1,356035506	0,230808373	5,875157342	4,22E-09	9,64E-08	SH2D1B	SH2 domain containing 1B	117157	7,016055162	Upregulated	69,28516717
ENSG00000188822.7	27,8896831	86,03619688	1,314920974	0,223867197	5,873665232	4,26E-09	9,70E-08	CNR2	cannabinoid receptor 2 (macrophage)	1269	7,013346801	Upregulated	100,5728253
ENSG00000181577.15	146,5482026	77,96880677	-1,05407307	0,180069063	-5,853715527	4,81E-09	1,09E-07	C6orf223	chromosome 6 open reading frame 223	221416	6,96174178	Downregulated	60,82395781
ENSG00000147234.10	13,35172578	85,14874205	1,420112158	0,243731659	5,826539569	5,66E-09	1,27E-07	FRMPD3	FERM and PDZ domain containing 3	84443	6,895076445	Upregulated	103,0979961
ENSG00000185090.14	28,44903457	74,21002698	1,217249561	0,209174957	5,819289162	5,91E-09	1,32E-07	MANEAL	mannosidase, endo-alpha-like	149175	6,878007107	Upregulated	85,65027509
ENSG00000145649.7	4666,399826	11721,19159	1,136757488	0,19562517	5,810895849	6,21E-09	1,38E-07	GZMA	granzyme A (granzyme 1, cytotoxic T-lymphocyte-associated serine esterase 3)	3001	6,858576104	Upregulated	13484,88953
ENSG00000245848.2	3,967566885	28,37145578	1,448351228	0,249995556	5,793507899	6,89E-09	1,52E-07	CEBPA	CCAAT/enhancer binding protein (C/EBP), alpha	1050	6,817043737	Upregulated	34,472428
ENSG00000172828.12	160,0313252	58,61677199	-1,425320153	0,246053809	-5,792717295	6,93E-09	1,53E-07	CES3	carboxylesterase 3	23491	6,816148839	Downregulated	33,26313369
ENSG00000173110.7	4,614269507	28,17441473	1,434195911	0,247597281	5,792454194	6,94E-09	1,53E-07	HSPA6	heat shock 70kDa protein 6 (HSP70B')	3310	6,81606556	Upregulated	34,06445104
ENSG00000189420.8	18,48130112	54,61481079	1,288644274	0,222596044	5,78916072	7,07E-09	1,55E-07	ZFP92	ZFP92 zinc finger protein	139735	6,808709882	Upregulated	63,64818821
ENSG00000240403.5	32,09582048	90,75105484	1,308842803	0,226393766	5,781266966	7,41E-09	1,62E-07	KIR3DL2	killer cell immunoglobulin-like receptor, three domains, long cytoplasmic tail, 2	3812	6,790051413	Upregulated	105,4148634
ENSG00000196781.13	16,51697016	54,04633281	1,310972394	0,228531521	5,736505801	9,66E-09	2,08E-07	TLE1	transducin-like enhancer of split 1 (E(sp1) homolog, Drosophila)	7088	6,681210744	Upregulated	63,42867348
ENSG00000135503.12	36,09715167	80,35431897	1,099082316	0,192326068	5,714681972	1,10E-08	2,36E-07	ACVR1B	activin A receptor, type IB	91	6,627647512	Upregulated	91,4186108
ENSG00000261801.5	54,36484551	108,2505001	1,018313228	0,178207319	5,71420542	1,10E-08	2,36E-07	LOXL1-AS1	LOXL1 antisense RNA 1	100287616	6,626996406	Upregulated	121,7219137
ENSG00000164484.11	81,19455319	182,6131924	1,103959324	0,193270894	5,71197918	1,12E-08	2,38E-07	TMEM200A	transmembrane protein 200A	114801	6,622811519	Upregulated	207,9678522
ENSG00000142173.14	238,0804565	820,3107671	1,338533651	0,234370344	5,711190362	1,12E-08	2,39E-07	COL6A2	collagen, type VI, alpha 2	1292	6,621555212	Upregulated	965,8683448
ENSG00000159784.17	20,91249172	77,62828071	1,312759321	0,230938789	5,684447073	1,31E-08	2,78E-07	FAM131B	family with sequence similarity 131, member B	9715	6,556253095	Upregulated	91,80722795
ENSG00000135525.18	95,23702724	44,3483347	-1,205170049	0,212179938	-5,679943458	1,35E-08	2,84E-07	MAP7	microtubule-associated protein 7	9053	6,546487297	Downregulated	31,62616156
ENSG00000196782.12	112,4975924	47,65844695	-1,317491907	0,232673071	-5,662416798	1,49E-08	3,12E-07	MAML3	mastermind-like 3 (Drosophila)	55534	6,506482044	Downregulated	31,4486606
ENSG00000123178.14	67,57049262	136,2651086	1,005426604	0,177679179	5,658663029	1,53E-08	3,18E-07	SPRYD7	SPRY domain containing 7	57213	6,497532461	Upregulated	153,4387626
ENSG00000072694.19	4,162091738	29,40078621	1,400746826	0,249877329	5,605737946	2,07E-08	4,23E-07	FCGR2B	Fc fragment of IgG, low affinity IIb, receptor (CD32)	2213	6,374015525	Upregulated	35,71045983
ENSG00000104970.10	20,57702887	203,7021476	1,403437087	0,250604021	5,600217748	2,14E-08	4,35E-07	KIR3DX1	killer cell immunoglobulin-like receptor, three domains, X1	NA	6,361253814	Upregulated	249,4834272
ENSG00000118200.14	31,02116318	95,08224169	1,25494552	0,224654597	5,586111028	2,32E-08	4,70E-07	CAMSAP2	calmodulin regulated spectrin-associated protein family, member 2	23271	6,327560498	Upregulated	111,0975113
ENSG00000073331.17	290,621169	164,2931463	-1,013496073	0,181655164	-5,579230732	2,42E-08	4,88E-07	ALPK1	alpha-kinase 1	80216	6,311975252	Downregulated	132,7111407
ENSG00000143195.12	57,233532	22,46249108	-1,356259296	0,243111268	-5,578759499	2,42E-08	4,88E-07	ILDR2	immunoglobulin-like domain containing receptor 2	387597	6,311331403	Downregulated	13,76973085
ENSG00000136573.12	57,74369717	146,3072231	1,109914014	0,199122479	5,574026697	2,49E-08	5,00E-07	BLK	B lymphoid tyrosine kinase	640	6,301115263	Upregulated	168,4481045
ENSG00000091844.7	10,70178723	37,81305934	1,304195508	0,234447789	5,562839877	2,65E-08	5,31E-07	RGS17	regulator of G-protein signaling 17	26575	6,274826036	Upregulated	44,59087736
ENSG00000147119.3	570,2102285	324,0910727	-1,004634809	0,182500092	-5,504845496	3,69E-08	7,29E-07	CHST7	carbohydrate (N-acetylglucosamine 6-O) sulfotransferase 7	56548	6,137447714	Downregulated	262,5612837
ENSG00000107551.20	16,33712719	47,14955082	1,223089637	0,22459465	5,445764791	5,16E-08	1,00E-06	RASSF4	Ras association (RalGDS/AF-6) domain family member 4	83937	5,998748451	Upregulated	54,85265672
ENSG00000160318.6	18,26255317	73,78346132	1,287182167	0,236416673	5,444549039	5,19E-08	1,00E-06	CLDND2	claudin domain containing 2	125875	5,998049985	Upregulated	87,66368836
ENSG00000136231.13	31,65318392	8,522387065	-1,261596237	0,231718729	-5,444515597	5,19E-08	1,00E-06	IGF2BP3	insulin-like growth factor 2 mRNA binding protein 3	10643	5,998049985	Downregulated	2,73968785
ENSG00000276141.4	13,05516789	59,36319295	1,328401811	0,24410667	5,441890678	5,27E-08	1,02E-06	WHAMMP3	WAS protein homolog associated with actin, golgi membranes and microtubules pseudogene 3	NA	5,993035726	Upregulated	70,94019921
ENSG00000170476.15	168,389273	77,94415364	-1,245071259	0,22881858	-5,44130314	5,29E-08	1,02E-06	MZB1	marginal zone B and B1 cell-specific protein	51237	5,992461217	Downregulated	55,33287381
ENSG00000277586.1	2,042140898	26,19113945	1,371678067	0,252200409	5,438841553	5,36E-08	1,03E-06	NEFL	neurofilament, light polypeptide	4747	5,987981611	Upregulated	32,22838909
ENSG00000171617.13	78,41321785	178,1584466	1,041903761	0,191702317	5,435008713	5,48E-08	1,05E-06	ENC1	ectodermal-neural cortex 1 (with BTB domain)	8507	5,97965429	Upregulated	203,0947538
ENSG00000054654.15	2633,338468	6073,751713	1,097711504	0,202073267	5,432245055	5,56E-08	1,06E-06	SYNE2	spectrin repeat containing, nuclear envelope 2	23224	5,97342935	Upregulated	6933,855025
ENSG00000150045.11	7,108588174	30,62753516	1,321810489	0,244220464	5,412365812	6,22E-08	1,18E-06	KLRF1	killer cell lectin-like receptor subfamily F, member 1	51348	5,927632134	Upregulated	36,50727191
ENSG00000177311.10	1146,418403	2597,368278	1,01392271	0,187342603	5,412131016	6,23E-08	1,18E-06	ZBTB38	zinc finger and BTB domain containing 38	253461	5,927563718	Upregulated	2960,105746
ENSG00000150681.9	3,709913969	25,56147357	1,358694958	0,251190395	5,409024323	6,34E-08	1,20E-06	RGS18	regulator of G-protein signaling 18	64407	5,921029661	Upregulated	31,02436347
ENSG00000163629.12	112,298667	500,8205143	1,317891168	0,245295281	5,372672325	7,76E-08	1,45E-06	PTPN13	protein tyrosine phosphatase, non-receptor type 13 (APO-1/CD95 (Fas)-associated phosphatase)	5783	5,840107739	Upregulated	597,9509761
ENSG00000129595.12	12,17503545	40,52453709	1,253135605	0,233891226	5,357770901	8,43E-08	1,56E-06	EPB41L4A	erythrocyte membrane protein band 4.1 like 4A	64097	5,807681322	Upregulated	47,6119125
ENSG00000143797.11	33,92875465	87,61810126	1,161530038	0,21749705	5,340440432	9,27E-08	1,69E-06	MBOAT2	membrane bound O-acyltransferase domain containing 2	129642	5,771433395	Upregulated	101,0404379
ENSG00000068137.14	23,83476507	70,34742103	1,210672489	0,227466859	5,322412656	1,02E-07	1,85E-06	PLEKHH3	pleckstrin homology domain containing, family H (with MyTH4 domain) member 3	79990	5,733105512	Upregulated	81,97558501
ENSG00000198756.10	95,15921729	317,140137	1,112570732	0,20924909	5,316968069	1,06E-07	1,90E-06	COLGALT2	collagen beta(1-O)galactosyltransferase 2	23127	5,721061691	Upregulated	372,6353669
ENSG00000182621.16	16,5947801	62,22451762	1,261911124	0,237947497	5,303317501	1,14E-07	2,03E-06	PLCB1	phospholipase C, beta 1 (phosphoinositide-specific)	23236	5,691849778	Upregulated	73,631952
ENSG00000142512.14	27,72452201	73,1921959	1,151250203	0,217820716	5,285310903	1,25E-07	2,23E-06	SIGLEC10	sialic acid binding Ig-like lectin 10	89790	5,652352523	Upregulated	84,55911437
ENSG00000165025.14	13,00158104	58,90165039	1,288774531	0,245278451	5,254332488	1,49E-07	2,61E-06	SYK	spleen tyrosine kinase	6850	5,583915531	Upregulated	70,37666773
ENSG00000228695.9	10,56084923	40,18852331	1,287631065	0,245370693	5,247697064	1,54E-07	2,69E-06	CES1P1	carboxylesterase 1 pseudogene 1	51716	5,569459431	Upregulated	47,59544183
ENSG00000273445.1	16,63368507	45,69450629	1,176032951	0,224126448	5,247185058	1,54E-07	2,70E-06	RP11-1399P15.1	NA	NA	5,568714629	Upregulated	52,95971159
ENSG00000165349.11	41,96592145	12,86590795	-1,277257333	0,243851524	-5,237848481	1,62E-07	2,82E-06	SLC7A3	solute carrier family 7 (cationic amino acid transporter, y+ system), member 3	84889	5,549950528	Downregulated	5,590904574
ENSG00000281179.1	590,6764233	325,8106469	-1,001523271	0,191265452	-5,236299912	1,64E-07	2,84E-06	RP11-294C11.4	NA	NA	5,546765874	Downregulated	259,5942028
ENSG00000017427.15	64,84274413	25,7670575	-1,297412724	0,248564152	-5,219629281	1,79E-07	3,09E-06	IGF1	insulin-like growth factor 1 (somatomedin C)	3479	5,50989948	Downregulated	15,99813585
ENSG00000175556.16	1,551058159	20,3908602	1,31533923	0,252229321	5,214854583	1,84E-07	3,17E-06	LONRF3	LON peptidase N-terminal domain and ring finger 3	79836	5,499617873	Upregulated	25,10081071
ENSG00000130649.9	6,539695493	27,8533129	1,277859409	0,245550486	5,204059782	1,95E-07	3,34E-06	CYP2E1	cytochrome P450, family 2, subfamily E, polypeptide 1	1571	5,47571269	Upregulated	33,18171725
ENSG00000249978.1	16,33712719	47,08920043	1,185781718	0,228165195	5,197031563	2,02E-07	3,47E-06	TRGV7	T cell receptor gamma variable 7 (pseudogene)	NA	5,460193857	Upregulated	54,77721874
ENSG00000205133.11	10,62397729	39,23198603	1,243404155	0,240447085	5,171217417	2,33E-07	3,93E-06	TRIQK	triple QxxK/R motif containing	286144	5,405885118	Upregulated	46,38398821
ENSG00000178053.17	10,36632437	38,46391293	1,240512288	0,240810259	5,151409631	2,59E-07	4,34E-06	MLF1	myeloid leukemia factor 1	4291	5,362592296	Upregulated	45,48831007
ENSG00000240350.2	17,70834237	48,87966192	1,159189725	0,22515716	5,148358275	2,63E-07	4,39E-06	AC017002.1	NA	NA	5,357193706	Upregulated	56,6724918
ENSG00000230537.1	22,9693145	58,46367203	1,131793233	0,220286514	5,137823517	2,78E-07	4,63E-06	RP11-305L7.1	NA	100507103	5,334694805	Upregulated	67,33726141
ENSG00000144843.11	74,82515946	168,5630855	1,037467271	0,202772283	5,116415586	3,11E-07	5,16E-06	ADPRH	ADP-ribosylarginine hydrolase	141	5,287522353	Upregulated	191,997567
ENSG00000085741.12	0	15,67002743	1,277910954	0,249949061	5,112685549	3,18E-07	5,26E-06	WNT11	wingless-type MMTV integration site family, member 11	7481	5,279378082	Upregulated	19,58753428
ENSG00000256667.6	67,74079438	472,6769298	1,272066493	0,249487317	5,09872209	3,42E-07	5,63E-06	KLRAP1	killer cell lectin-like receptor subfamily A pseudogene 1	NA	5,249484497	Upregulated	573,9109637
ENSG00000105246.5	12,33065533	70,7431931	1,260308974	0,247750764	5,087003388	3,64E-07	5,96E-06	EBI3	Epstein-Barr virus induced 3	10148	5,224799301	Upregulated	85,34632755
ENSG00000092445.11	18,75363591	55,28474513	1,179230195	0,232097697	5,080749223	3,76E-07	6,15E-06	TYRO3	TYRO3 protein tyrosine kinase	7301	5,211355776	Upregulated	64,41752243
ENSG00000137959.15	16,51697016	50,29995344	1,192525675	0,236060085	5,051788728	4,38E-07	7,08E-06	IFI44L	interferon-induced protein 44-like	10964	5,15003956	Upregulated	58,74569926
ENSG00000196735.11	965,2859021	1806,321691	1,00488402	0,199392761	5,039721672	4,66E-07	7,51E-06	HLA-DQA1	major histocompatibility complex, class II, DQ alpha 1	3117	5,124331661	Upregulated	2016,580638
ENSG00000143545.8	59,36302406	26,81082267	-1,177442266	0,23563574	-4,996874687	5,83E-07	9,25E-06	RAB13	RAB13, member RAS oncogene family	5872	5,033824713	Downregulated	18,67277232
ENSG00000140450.8	52,81378735	18,40355094	-1,258065434	0,252067767	-4,990980983	6,01E-07	9,51E-06	ARRDC4	arrestin domain containing 4	91947	5,021820146	Downregulated	9,800991834
ENSG00000188372.14	66,05833943	22,64081551	-1,251346353	0,250918248	-4,987067948	6,13E-07	9,69E-06	ZP3	zona pellucida glycoprotein 3 (sperm receptor)	7784	5,013859375	Downregulated	11,78643453
ENSG00000090382.6	9,719621753	41,83591603	1,218098767	0,244410595	4,983821446	6,23E-07	9,83E-06	LYZ	lysozyme	4069	5,007400155	Upregulated	49,8649896
ENSG00000117594.9	8,698551303	32,24167829	1,208358125	0,242670418	4,979420783	6,38E-07	1,00E-05	HSD11B1	hydroxysteroid (11-beta) dehydrogenase 1	3290	4,999598741	Upregulated	38,12746004
ENSG00000156234.7	1,05997542	34,38213842	1,246207979	0,250784997	4,969228597	6,72E-07	1,05E-05	CXCL13	chemokine (C-X-C motif) ligand 13	10563	4,97716527	Upregulated	42,71267916
ENSG00000186891.13	53,25128324	154,1151944	1,107132364	0,222878788	4,967419171	6,78E-07	1,06E-05	TNFRSF18	tumor necrosis factor receptor superfamily, member 18	8784	4,973940895	Upregulated	179,3311722
ENSG00000278058.1	22,32261188	52,22596132	1,07569374	0,217375284	4,948555867	7,48E-07	1,17E-05	RP11-70D24.2	NA	NA	4,932610365	Upregulated	59,70179868
ENSG00000180875.4	14,43592429	45,86060813	1,187464462	0,240110108	4,945499672	7,59E-07	1,18E-05	GREM2	gremlin 2, DAN family BMP antagonist	64388	4,926989049	Upregulated	53,71677909
ENSG00000131203.12	0	17,97144889	1,231319801	0,249019017	4,94468181	7,63E-07	1,19E-05	IDO1	indoleamine 2,3-dioxygenase 1	3620	4,926028313	Upregulated	22,46431112
ENSG00000135643.4	91,99837346	50,63184941	-1,001146744	0,202594369	-4,94163165	7,75E-07	1,20E-05	KCNMB4	potassium large conductance calcium-activated channel, subfamily M, beta member 4	27345	4,92005093	Downregulated	40,29021839
ENSG00000187240.13	40,42954517	105,6106356	1,077233271	0,218016913	4,941053681	7,77E-07	1,20E-05	DYNC2H1	dynein, cytoplasmic 2, heavy chain 1	79659	4,919172828	Upregulated	121,9059082
ENSG00000067840.12	8,168563593	29,42621835	1,19971065	0,243531045	4,926315044	8,38E-07	1,29E-05	PDZD4	PDZ domain containing 4	57595	4,888426756	Upregulated	34,74063204
ENSG00000188266.13	67,84282741	31,62051004	-1,136572487	0,231225019	-4,915439037	8,86E-07	1,36E-05	HYKK	hydroxylysine kinase	123688	4,866727805	Downregulated	22,5649307
ENSG00000259244.1	0	13,30378064	1,220087958	0,249080665	4,898364789	9,66E-07	1,48E-05	RP11-182J1.12	NA	NA	4,830956909	Upregulated	16,6297258
ENSG00000165424.6	16,27399913	50,39995251	1,178731334	0,242075959	4,869262273	1,12E-06	1,70E-05	ZCCHC24	zinc finger, CCHC domain containing 24	219654	4,770222369	Upregulated	58,93144086
ENSG00000254760.1	3,515389116	22,84140191	1,226440458	0,251883853	4,86907138	1,12E-06	1,70E-05	CTD-2616J11.3	NA	NA	4,770222369	Upregulated	27,6729051
ENSG00000104375.15	16,41493713	46,09575847	1,130924414	0,232271213	4,868982248	1,12E-06	1,70E-05	STK3	serine/threonine kinase 3	6788	4,770222369	Upregulated	53,51596381
ENSG00000144893.12	85,75523585	38,14458472	-1,169815459	0,240604276	-4,861989481	1,16E-06	1,75E-05	MED12L	mediator complex subunit 12-like	116931	4,757359547	Downregulated	26,24192194
ENSG00000179841.8	56,05684167	122,7614949	1,005843737	0,206884754	4,861855305	1,16E-06	1,75E-05	AKAP5	A kinase (PRKA) anchor protein 5	9495	4,757359547	Upregulated	139,4376582
ENSG00000142794.18	25,64347614	60,30909557	1,046145111	0,215247441	4,860197667	1,17E-06	1,76E-05	NBPF3	neuroblastoma breakpoint family, member 3	84224	4,754119262	Upregulated	68,97550042
ENSG00000137441.7	998,4358574	3200,605817	1,115492775	0,230266952	4,84434594	1,27E-06	1,89E-05	FGFBP2	fibroblast growth factor binding protein 2	83888	4,723450982	Upregulated	3751,148307
ENSG00000237248.4	7,872005708	30,79417777	1,193772307	0,247453532	4,824228204	1,41E-06	2,07E-05	LINC00987	long intergenic non-protein coding RNA 987	100499405	4,683341958	Upregulated	36,52472078
ENSG00000163464.7	36,40839143	82,87741848	1,032434399	0,21505339	4,800828285	1,58E-06	2,32E-05	CXCR1	chemokine (C-X-C motif) receptor 1	3577	4,635193217	Upregulated	94,49467524
ENSG00000165178.9	7,264208056	34,52155722	1,194311491	0,249630072	4,78432539	1,72E-06	2,49E-05	NCF1C	neutrophil cytosolic factor 1C pseudogene	654817	4,603694286	Upregulated	41,33589452
ENSG00000182636.5	70,42009669	24,67073271	-1,205488795	0,25199947	-4,78369576	1,72E-06	2,50E-05	NDN	necdin, melanoma antigen (MAGE) family member	4692	4,602716267	Downregulated	13,23339171
ENSG00000184545.10	4,006471855	23,82716104	1,198463066	0,252023005	4,7553717	1,98E-06	2,85E-05	DUSP8	dual specificity phosphatase 8	1850	4,54508894	Upregulated	28,78233334
ENSG00000126460.10	63,393719	28,2349694	-1,136317939	0,239138152	-4,751721674	2,02E-06	2,90E-05	PRRG2	proline rich Gla (G-carboxyglutamic acid) 2	5639	4,537624518	Downregulated	19,44528199
ENSG00000236790.5	6,048612754	26,41841225	1,183536092	0,24975563	4,738776432	2,15E-06	3,07E-05	LINC00299	long intergenic non-protein coding RNA 299	NA	4,51287554	Upregulated	31,51086212
ENSG00000162415.6	6,656410405	28,05318723	1,175839913	0,248383803	4,73396373	2,20E-06	3,14E-05	ZSWIM5	zinc finger, SWIM-type containing 5	57643	4,503696762	Upregulated	33,40238143
ENSG00000103175.10	0	18,79540792	1,163892689	0,247710051	4,698609057	2,62E-06	3,68E-05	WFDC1	WAP four-disulfide core domain 1	58189	4,434243122	Upregulated	23,4942599
ENSG00000149506.10	41,6062355	13,32440826	-1,138527792	0,242412047	-4,696663418	2,64E-06	3,71E-05	ZP1	zona pellucida glycoprotein 1 (sperm receptor)	22917	4,431147576	Downregulated	6,253951453
ENSG00000137801.10	14,51373423	42,49212379	1,120056386	0,2386748	4,692813759	2,69E-06	3,76E-05	THBS1	thrombospondin 1	7057	4,424517271	Upregulated	49,48672118
ENSG00000163106.10	6,695315376	24,82449167	1,157363911	0,247059782	4,684550049	2,81E-06	3,91E-05	HPGDS	hematopoietic prostaglandin D synthase	27306	4,408086892	Upregulated	29,35678574
ENSG00000211696.2	21,58341744	68,57925156	1,107310914	0,236736775	4,677392913	2,91E-06	4,04E-05	TRGV8	T cell receptor gamma variable 8	NA	4,393657918	Upregulated	80,32821009
ENSG00000092051.16	2,003235928	20,9767163	1,170888748	0,251589689	4,653961579	3,26E-06	4,48E-05	JPH4	junctophilin 4	84502	4,348547066	Upregulated	25,72008639
ENSG00000140104.13	9,189634043	29,59309368	1,125182048	0,242544577	4,639073204	3,50E-06	4,79E-05	C14orf79	chromosome 14 open reading frame 79	122616	4,320115406	Upregulated	34,69395859
ENSG00000135678.11	64,22540527	165,3280211	1,005083558	0,217487934	4,621330214	3,81E-06	5,18E-05	CPM	carboxypeptidase M	1368	4,285778692	Upregulated	190,603675

ENSG00000244649.4	2,611033579	27,2543743	1,156011958	0,251532766	4,595870257	4,31E-06	5,75E-05	CTD-2377D24.6	NA	NA	4,240426317	Upregulated	33,41520948
ENSG00000109906.13	44,53805006	126,1142862	1,009792833	0,220390046	4,581844113	4,61E-06	6,11E-05	ZBTB16	zinc finger and BTB domain containing 16	7704	4,214058177	Upregulated	146,5083452
ENSG00000128596.16	19,73580139	52,41333442	1,06150592	0,231799589	4,579412435	4,66E-06	6,17E-05	CCDC136	coiled-coil domain containing 136	64753	4,209707569	Upregulated	60,58271768
ENSG00000198478.7	43,93025241	18,09312092	-1,14835404	0,251068244	-4,573872109	4,79E-06	6,32E-05	SH3BGRL2	SH3 domain binding glutamate-rich protein like 2	83699	4,199257913	Downregulated	11,63383805
ENSG00000117586.10	20,73264875	149,7807396	1,152170582	0,25227857	4,56705689	4,95E-06	6,52E-05	TNFSF4	tumor necrosis factor (ligand) superfamily, member 4	7292	4,185829987	Upregulated	182,0427623
ENSG00000152213.3	57,21885012	25,97025047	-1,11501941	0,24431541	-4,563852153	5,02E-06	6,60E-05	ARL11	ADP-ribosylation factor-like 11	115761	4,180584654	Downregulated	18,15810056
ENSG00000169432.14	40,68719808	14,22397092	-1,123774986	0,247654219	-4,537677527	5,69E-06	7,41E-05	SCN9A	sodium channel, voltage-gated, type IX, alpha subunit	6335	4,130352465	Downregulated	7,608164135
ENSG00000131398.13	96,25809769	43,27593682	-1,079832208	0,238832657	-4,521292113	6,15E-06	7,94E-05	KCNC3	potassium voltage-gated channel, Shaw-related subfamily, member 3	3748	4,100099416	Downregulated	30,0303966
ENSG00000156463.17	109,9937325	59,55552682	-1,029346205	0,227980129	-4,515069837	6,33E-06	8,16E-05	SH3RF2	SH3 domain containing ring finger 2	153769	4,088361974	Downregulated	46,9459754
ENSG00000140009.18	3,141021289	23,17025072	1,137202714	0,252007095	4,512582134	6,40E-06	8,23E-05	ESR2	estrogen receptor 2 (ER beta)	2100	4,084624382	Upregulated	28,17755808
ENSG00000205213.13	2,64993855	18,34448329	1,132533764	0,252026038	4,493717292	7,00E-06	8,92E-05	LGR4	leucine-rich repeat containing G protein-coupled receptor 4	55366	4,049773842	Upregulated	22,26811948
ENSG00000224596.7	67,51690577	33,93706738	-1,014007292	0,225848469	-4,489768275	7,13E-06	9,08E-05	ZMIZ1-AS1	ZMIZ1 antisense RNA 1	NA	4,042055645	Downregulated	25,54210778
ENSG00000198088.10	62,76169826	29,99523908	-1,062309964	0,236949842	-4,483269354	7,35E-06	9,32E-05	NUP62CL	nucleoporin 62kDa C-terminal like	54830	4,030491991	Downregulated	21,80362428
ENSG00000262874.1	0	11,52566376	1,102713055	0,246666474	4,470461825	7,81E-06	9,85E-05	C19orf84	chromosome 19 open reading frame 84	147646	4,006453543	Upregulated	14,4070797
ENSG00000198734.10	1199,372388	682,9648881	-1,023215255	0,229670721	-4,455140166	8,38E-06	0,000105346	F5	coagulation factor V (proaccelerin, labile factor)	2153	3,977383556	Downregulated	553,863013
ENSG00000105370.7	2,081045869	17,7301776	1,118855178	0,251698058	4,445227696	8,78E-06	0,000109902	LIM2	lens intrinsic membrane protein 2, 19kDa	3982	3,958993558	Upregulated	21,64246053
ENSG00000211829.7	284,4595017	1876,945329	1,113396695	0,251769711	4,422282144	9,77E-06	0,00012078	TRDC	T cell receptor delta constant	NA	3,918004902	Upregulated	2275,066786
ENSG00000090104.11	128,2320626	375,6424737	1,048569793	0,237792634	4,409597448	1,04E-05	0,000127405	RGS1	regulator of G-protein signaling 1	5996	3,894813902	Upregulated	437,4950764
ENSG00000173809.15	57,24307322	24,60456857	-1,096091127	0,248836941	-4,404856937	1,06E-05	0,000129882	TDRD12	tudor domain containing 12	91646	3,886452551	Downregulated	16,4449424
ENSG00000119147.9	23,78117822	8,498899502	-1,096526973	0,248940318	-4,404778552	1,06E-05	0,000129882	C2orf40	chromosome 2 open reading frame 40	84417	3,886452551	Downregulated	4,678329824
ENSG00000185745.9	9,072919131	29,50776648	1,078953501	0,245088639	4,402299134	1,07E-05	0,000131082	IFIT1	interferon-induced protein with tetratricopeptide repeats 1	3434	3,882457209	Upregulated	34,61647831
ENSG00000152670.18	45,87036027	21,01900557	-1,075859751	0,244491992	-4,400388509	1,08E-05	0,000131849	DDX4	DEAD (Asp-Glu-Ala-Asp) box polypeptide 4	54514	3,879922939	Downregulated	14,8061669
ENSG00000107317.11	23,96102119	64,21036843	1,028352818	0,234300812	4,389027962	1,14E-05	0,000138006	PTGDS	prostaglandin D2 synthase 21kDa (brain)	5730	3,860101557	Upregulated	74,27270524
ENSG00000224863.2	0	11,41185219	1,076450119	0,245870613	4,378116223	1,20E-05	0,000144459	LINC01398	NA	101929651	3,8402541	Upregulated	14,26481523
ENSG00000108702.3	12,54940328	61,11436502	1,095845004	0,250516863	4,374336294	1,22E-05	0,00014677	CCL1	chemokine (C-C motif) ligand 1	6346	3,833363396	Upregulated	73,25560546
ENSG00000041982.15	4,045376826	20,12153665	1,09945792	0,252101081	4,361178921	1,29E-05	0,000155198	TNC	tenascin C	3371	3,809112516	Upregulated	24,1405766
ENSG00000114455.13	44,81038485	21,27379984	-1,046058863	0,240607528	-4,347573295	1,38E-05	0,000163591	HHLA2	HERV-H LTR-associating 2	11148	3,786240225	Downregulated	15,38965358
ENSG00000274536.4	67,63362068	28,62665432	-1,087087619	0,250839564	-4,33379648	1,47E-05	0,00017355	RP6-159A1.4	NA	NA	3,760574657	Downregulated	18,87491274
ENSG00000260027.4	8,737456274	26,68581292	1,057012429	0,244640701	4,320672824	1,56E-05	0,000182623	HOXB7	homeobox B7	3217	3,73844338	Upregulated	31,17290208
ENSG00000214279.12	15,19934183	47,83102062	1,057530919	0,244778378	4,320360837	1,56E-05	0,000182751	SCART1	scavenger receptor protein family member	619207	3,738139194	Upregulated	55,98894031
ENSG00000169122.11	23,31945924	54,83834415	1,001555262	0,233157102	4,295624076	1,74E-05	0,000201725	FAM110B	family with sequence similarity 110, member B	90362	3,695239713	Upregulated	62,71806538
ENSG00000008441.16	0	10,95280836	1,053550109	0,245277008	4,295348016	1,74E-05	0,000201748	NFIX	nuclear factor I/X (CCAAT-binding transcription factor)	4784	3,695191465	Upregulated	13,69101045
ENSG00000240204.2	3,593199057	20,03709649	1,082042464	0,252260499	4,289385258	1,79E-05	0,000206659	SMKR1	small lysine-rich protein 1	100287482	3,684745141	Upregulated	24,14807085
ENSG00000248871.1	1,05997542	22,36327187	1,059853233	0,247559959	4,281198126	1,86E-05	0,000213812	TNFSF12-TNFSF13	TNFSF12-TNFSF13 readthrough	407977	3,669967468	Upregulated	27,68909598
ENSG00000225632.1	83,04216924	39,72463506	-1,035048169	0,242199893	-4,273528588	1,92E-05	0,000220773	RP5-997D24.3	NA	NA	3,656053669	Downregulated	28,89525151
ENSG00000179934.6	836,1964901	477,2098493	-1,015279014	0,237686171	-4,271510664	1,94E-05	0,000222384	CCR8	chemokine (C-C motif) receptor 8	1237	3,652896697	Downregulated	387,4631891
ENSG00000167037.18	3,102116318	19,56312341	1,073419619	0,251999809	4,259604893	2,05E-05	0,000233428	SGSM1	small G protein signaling modulator 1	129049	3,631847501	Upregulated	23,67837518
ENSG00000135253.13	2,081045869	16,9971888	1,061288272	0,250871157	4,230411686	2,33E-05	0,000263331	KCP	kielin/chordin-like protein	375616	3,579498717	Upregulated	20,72622453
ENSG00000228140.1	0,52998771	10,92349458	1,045404476	0,247619416	4,22181948	2,42E-05	0,000271709	RP3-467K16.4	NA	NA	3,565895206	Upregulated	13,52187129
ENSG00000114529.12	53,52361803	22,93295886	-1,062211948	0,251618527	-4,221517242	2,43E-05	0,000271889	C3orf52	chromosome 3 open reading frame 52	79669	3,5656087	Downregulated	15,28529407
ENSG00000162373.12	60,39877638	27,14114652	-1,043726286	0,248906995	-4,193238063	2,75E-05	0,000303571	BEND5	BEN domain containing 5	79656	3,517739565	Downregulated	18,82673905
ENSG00000163814.7	31,80880381	11,3297961	-1,015832659	0,242620927	-4,18691277	2,83E-05	0,000311525	CDCP1	CUB domain containing protein 1	64866	3,50650656	Downregulated	6,210044169
ENSG00000258818.3	15,01949885	45,89103677	1,031978361	0,247096412	4,176419846	2,96E-05	0,000324009	RNASE4	ribonuclease, RNase A family, 4	6038	3,489443422	Upregulated	53,60892125
ENSG00000260828.1	12,62721322	38,6946199	1,018142973	0,244105025	4,170921818	3,03E-05	0,000329579	HMGB3P32	high mobility group box 3 pseudogene 32	NA	3,482040234	Upregulated	45,21147157
ENSG00000121594.11	8,737456274	40,57786936	1,04447641	0,251003463	4,161203177	3,17E-05	0,000341893	CD80	CD80 molecule	941	3,466109817	Upregulated	48,53797263
ENSG00000160062.14	4,162091738	18,26895337	1,04776226	0,252126935	4,155693473	3,24E-05	0,000348636	ZBTB8A	zinc finger and BTB domain containing 8A	653121	3,4576282	Upregulated	21,79566877
ENSG00000120708.16	0,982165478	15,32046099	1,033439328	0,249473388	4,142483229	3,44E-05	0,00036647	TGFBI	transforming growth factor, beta-induced, 68kDa	7045	3,435961732	Upregulated	18,90503486
ENSG00000057704.10	6,734220346	28,40320762	1,04150572	0,25148812	4,141371456	3,45E-05	0,000367822	TMCC3	transmembrane and coiled-coil domain family 3	57458	3,434362489	Upregulated	33,82045444
ENSG00000148053.15	13,50734566	74,49153404	1,043024288	0,251986576	4,139205764	3,49E-05	0,000370543	NTRK2	neurotrophic tyrosine kinase, receptor, type 2	4915	3,43116164	Upregulated	89,73758113
ENSG00000019485.12	22,11340515	70,05660381	1,002472278	0,243189464	4,1221863	3,75E-05	0,000396456	PRDM11	PR domain containing 11	56981	3,401804486	Upregulated	82,04240347
ENSG00000269930.1	4,575364536	30,40316622	1,032556245	0,252131605	4,095306667	4,22E-05	0,000440295	RP11-932O9.9	NA	NA	3,356256626	Upregulated	36,86011664
ENSG00000267452.1	3,632104028	27,21499689	1,026837039	0,251953262	4,075506032	4,59E-05	0,000474372	RP11-1018N14.5	NA	NA	3,323881129	Upregulated	33,11072011
ENSG00000166825.13	7,599670913	30,21710095	1,017988717	0,249903246	4,073531388	4,63E-05	0,000477813	ANPEP	alanyl (membrane) aminopeptidase	290	3,320741634	Upregulated	35,87145845
ENSG00000169245.5	6,048612754	31,06381083	1,019823251	0,252116549	4,045046851	5,23E-05	0,000535099	CXCL10	chemokine (C-X-C motif) ligand 10	3627	3,271566133	Upregulated	37,31761036
ENSG00000189221.9	55,74560191	23,63134648	-1,016482035	0,252122792	-4,031694337	5,54E-05	0,000562934	MAOA	monoamine oxidase A	4128	3,249542436	Downregulated	15,60278262
ENSG00000074590.13	1,512153188	13,37181018	1,003512597	0,250089744	4,012609956	6,01E-05	0,000605221	NUAK1	NUAK family, SNF1-like kinase, 1	9891	3,218086149	Upregulated	16,33672443
ENSG00000128040.10	32,57222134	12,82947477	-1,003381249	0,251283005	-3,993032679	6,52E-05	0,000652212	SPINK2	serine peptidase inhibitor, Kazal type 2 (acrosin-trypsin inhibitor)	6691	3,185611344	Downregulated	7,893788122

Supplementary Table 1. List of differentially regulated genes between Expamers and Dynabeads. All Expamers conditions were collapsed and treated as technical replicates for each donor. Differential expression analysis was performed in R using the DESeq2 package and yielded 500+ genes with $|log_2FC| \geq 1$ and Benjamini-Hochberg adjusted p-value ≤ 0.1.
